# Design, Synthesis, and Biological Evaluation of BODIPY-Caged Resiquimod as a Dual-Acting Phototherapeutic

**DOI:** 10.1021/acs.jmedchem.4c02606

**Published:** 2025-02-17

**Authors:** Eslam Roshdy, Haruto Taniguchi, Yoki Nakamura, Haruko Takahashi, Yutaka Kikuchi, Ismail Celik, Elsayed S. I. Mohammed, Yasuhiro Ishihara, Norimitsu Morioka, Manabu Abe

**Affiliations:** † Department of Chemistry, Graduate School of Advanced Science and Engineering, Hiroshima University, Higashi-Hiroshima City, Hiroshima 739-8526, Japan; ‡ Medicinal Chemistry Department, Faculty of Pharmacy, Minia University, Minia 61519, Egypt; § Department of Pharmacology, Graduate School of Biomedical & Health Sciences, Hiroshima University, Kasumi 1-2-3, Minami-ku, Hiroshima 734-8553, Japan; ∥ Graduate School of Integrated Sciences for Life, Hiroshima University, 1-3-1 Kagamiyama, Higashi-Hiroshima, Hiroshima 739-8526, Japan; ⊥ Department of Pharmaceutical Chemistry, Faculty of Pharmacy, Erciyes University, 38039 Kayseri, Turkey; # Avian Research Center, King Faisal University, Al Hofuf, Al-Ahsa 31982, Saudi Arabia; ¶ Department of Histology, Faculty of Veterinary Medicine, South Valley University, Qena 83523, Egypt; ∇ Program of Biomedical Science, Graduate School of Integrated Sciences for Life, Hiroshima University, 1-7-1, Kagamiyama, Higashi-Hiroshima, Hiroshima 739-8521, Japan

## Abstract

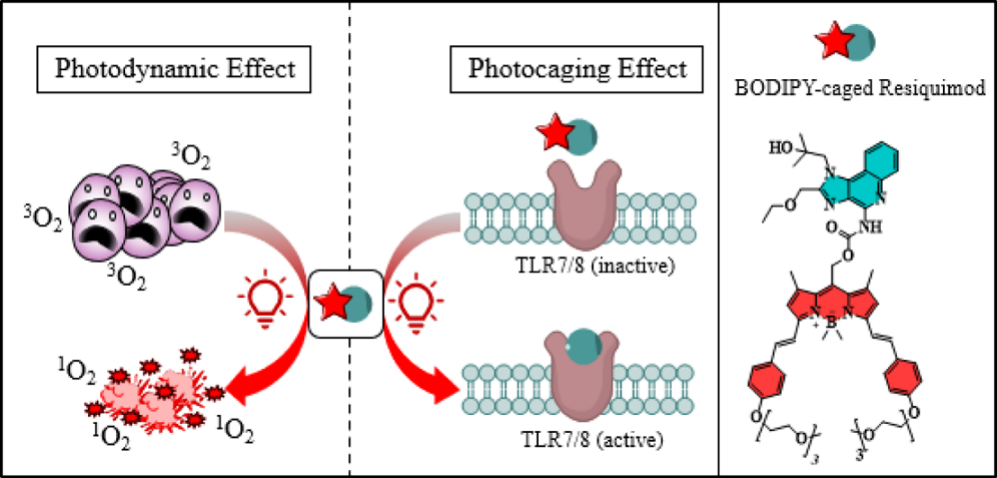

Resiquimod, an imidazoquinoline scaffold, exhibits potent immunotherapeutic activity but is associated with off-target effects, limiting its clinical utility. To address this limitation, we developed a novel BODIPY-caged resiquimod that is responsive to red light, combining photocaging and photodynamic therapy functionalities. Molecular docking studies guided identification of the optimal caging site for resiquimod, effectively masking its immune activity. BODIPY-caged resiquimod remained inactive under dark conditions, effectively masking resiquimod’s immunostimulatory effects. However, red light irradiation precisely uncaged resiquimod, inducing robust immune activation, even in the presence of *N*-acetyl cysteine as an antioxidant. Notably, the attachment of resiquimod to BODIPY reduced the dark toxicity typically associated with BODIPY as a photosensitizer. In 3D spheroid models of HeLa and A549 cells, BODIPY-caged resiquimod demonstrated spatiotemporal control over cytotoxicity, significantly enhancing cell death only upon irradiation. This dual-function therapeutic approach highlights a “win–win” strategy: precise, red-light-mediated control of immune activation and photodynamic efficacy with reduced collateral toxicity.

## 1 Introduction

Immunotherapy has revolutionized cancer treatment, including vaccines, monoclonal antibodies, adoptive cell therapies, and immune checkpoint inhibitors (ICIs).^1^ Unlike traditional therapies, immunotherapy can produce widespread antitumor effects, including the targeting of abscopal tumors that are difficult to treat with conventional methods.^2^ Moreover, it promotes robust immunological memory, enabling the immune system to combat rechallenged tumors effectively.^3^ Among the various immunotherapeutic approaches, activating toll-like receptors (TLRs) has emerged as a promising strategy due to their pivotal role in bridging innate and adaptive immune responses.^1^

TLRs are a family of transmembrane receptors expressed by various immune (e.g., macrophages, dendritic cells, lymphocytes) and nonimmune (e.g., epithelial cells, fibroblasts) cells.^4,5^ Of the 10 TLRs expressed in humans, 6 are found on cell surfaces (TLR1, 2, 4, 5, 6, and 10), and 4 are localized to endosomes (TLR3, 7, 8, and 9).^4−6^ The former recognizes proteins and lipids, whereas the latter engages nucleic acids.^7^ TLR7/8 is overexpressed in several cancers, including pancreatic, lung, and esophageal cancers, making them attractive targets for anticancer therapies.^1^ The antitumor effects of TLRs are mediated by the secretion of pro-inflammatory cytokines and the induction of tumor cell death, whereas their pro-tumor effects include facilitating cancer cell proliferation, survival, metastasis, and immunosuppression.^7,8^

Resiquimod is a potent TLR7/8 agonist with significant antiviral and anticancer activities.^9^ Resiquimod agonistic activity on TLR7/8 is portrayed by releasing interferon-alpha (IFN-α) and other cytokines.^9,10^ Despite its therapeutic potential, the clinical application of resiquimod has been limited by severe side effects, including poor tolerability and systemic cytokine release, which result in flu-like symptoms and other adverse reactions.^11−13^ Previous efforts to mitigate these issues have included the incorporation of resiquimod into nanoparticles to enhance its uptake by antigen-presenting cells and improve its therapeutic index.^14,15^ However, these strategies do not fully address the need for precise spatiotemporal control of resiquimod’s activity to minimize off-target effects.

Photopharmacology, particularly photocaging techniques, offers a novel solution to these challenges. Photocaging involves the covalent attachment of a photolabile protecting group (PPG) to a drug, rendering it inactive until exposure to light of a specific wavelength releases the active compound.^16,17^ This approach allows for the precise control of drug activation, minimizing systemic toxicity and enhancing therapeutic efficacy.^18^*o*-Nitroaryl PPGs are the most common PPG class, which uncages the substrate through intramolecular rearrangement reactions upon exposure to UV light.^19,20^ Photo-S_N_1 PPGs are another class that release the cargo via direct bond dissociation. Numerous PPGs, such as coumarins,^21,22^ quinolines,^23^ and BODIPYs,^24,25^ belong to this class. However, traditional UV-responsive PPGs, such as *o*-nitroaryl groups, suffer from poor tissue penetration and high phototoxicity. More recently, visible-light-activatable BODIPYs have emerged as promising alternatives due to their superior photophysical properties and compatibility with the phototherapeutic window.^26^

**Figure 1:**
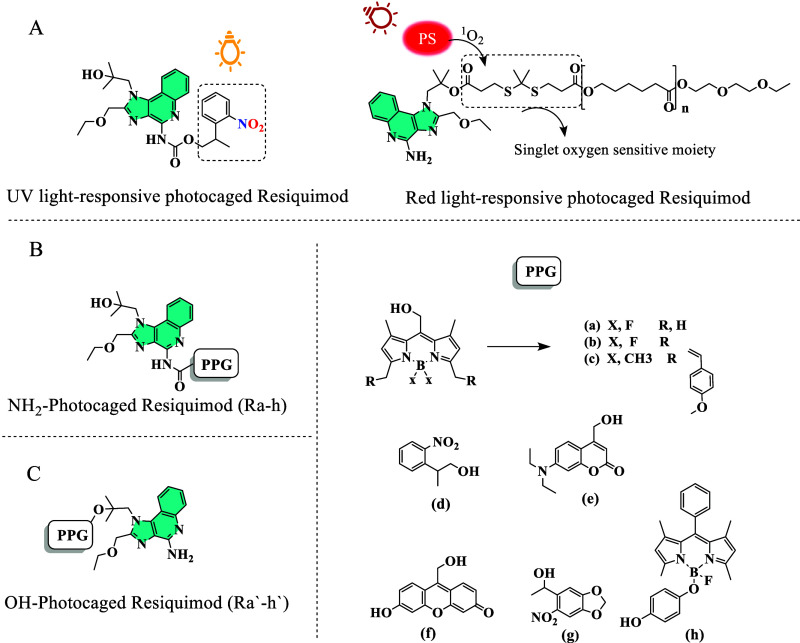
(A) Previously reported photocaged resiquimod; (B,C) NH_2_- and OH-photocaged resiquimod analogues designed for docking studies.

Resiquimod was previously photocaged in two distinct studies. The first example, conducted by Ryu et al., utilized an *o*-nitrophenyl ethyl PPG to cage the amine moiety of resiquimod through a carbamate linker, enabling mechanistic studies on its TLR7/8 activation (Figure 1A).^9^ However, this UV-light-activable resiquimod cannot be repurposed as a photochemotherapeutic due to the inherent limitations of UV light, including poor tissue penetration and high phototoxicity. In 2023, Wan et al. introduced a different approach, incorporating resiquimod into a photoactivatable nanoformulation. This system utilized a red-light-responsive photosensitizer to generate singlet oxygen, which then cleaved a singlet oxygen-sensitive linker attached to the hydroxyl group of resiquimod (Figure 1A).^27^ Although effective, this method relies on a complex pharmaceutical formulation with a photosensitizer, limiting its practicality.

In this study, we designed BODIPY-caged resiquimod as a dual-acting compound to precisely control resiquimod’s immunomodulatory effects, avoiding its side effects, while simultaneously masking BODIPY’s inherent dark toxicity. This innovative “win–win” approach leverages resiquimod to minimize BODIPY’s toxicity under dark conditions and BODIPY to enable light-triggered activation of resiquimod for targeted TLR7 activation and robust immunostimulation, overcoming the limitations of earlier approaches by enabling drug activation under safer light, eliminating the need for UV light or a multicomponent formulation.

## 2 Results and Discussion

### 2.1 Rational Design of BODIPY-Caged Resiquimod

Resiquimod was photocaged in two previous studies. The first involved caging at the amine moiety using an *o*-nitrophenyl ethyl PPG,^9^ while the second involved caging at the hydroxyl group with a singlet oxygen-sensitive linker, which is cleaved upon singlet oxygen generation by a red light-responsive photosensitizer (Figure 1A).^27^ To identify the optimal caging site for resiquimod, a structure-based drug design approach was employed. According to the guidelines established by Szymanski and Feringa for molecular design in photopharmacology, a structure-based drug design approach is recommended when the target structure is known and the ligand binding site is well defined.^28^ To assess and predict the binding affinities of various caged derivatives of resiquimod at the TLR7/8 binding site, molecular docking studies were conducted. These studies involved two groups of compounds to determine the binding affinities when resiquimod is caged at either its NH_2_ (Figure 1B) or its OH (Figure 1C) functionalities. Additionally, it predicts whether changing the PPG has different binding affinities or not. The crystal structures of human TLR8 (PDB ID: 3W3L, resolution 2.33 Å) and monkey TLR7 (PDB ID: 5GMH, resolution 2.20 Å), both complexed with resiquimod, were obtained from the Protein Data Bank (PDB). Table 1 summarizes the best docking scores for each compound with TLR8, while Table S1 presents the binding scores with TLR7. Resiquimod achieved a binding energy of −8.5 kcal/mol for TLR8. Notably, the interaction between resiquimod and TLR8 involved π–π stacking between the benzene rings of imidazoquinoline and Phe^405^.

**Table 1:** Summary of the Docking Scores, Indicative of Binding Energies (kcal mol^–1^), for Amino-Caged Resiquimods (**Ra–h**) and Hydroxy-Caged Resiquimods (**Ra**′**–h**′) in Association with TLR8 (PDB ID: 3W3L)

NH_2_-caging		OH-caging	
ligand	docking score	ligand	docking score
resiquimod	–8.506		
**Ra**	–3.101	**Ra**	–7.009
**Rb**		**Rb′**	
**Rc**		**Rc′**	
**Rd**	–3.536	**Rd′**	–6.046
**Re**	–3.322	**Re′**	–6.839
**Rf**	–5.483	**Rf′**	–7.374
**Rg**	–3.899	**Rg′**	–8.172
**Rh**		**Rh′**	–7.260

**Figure 2:**
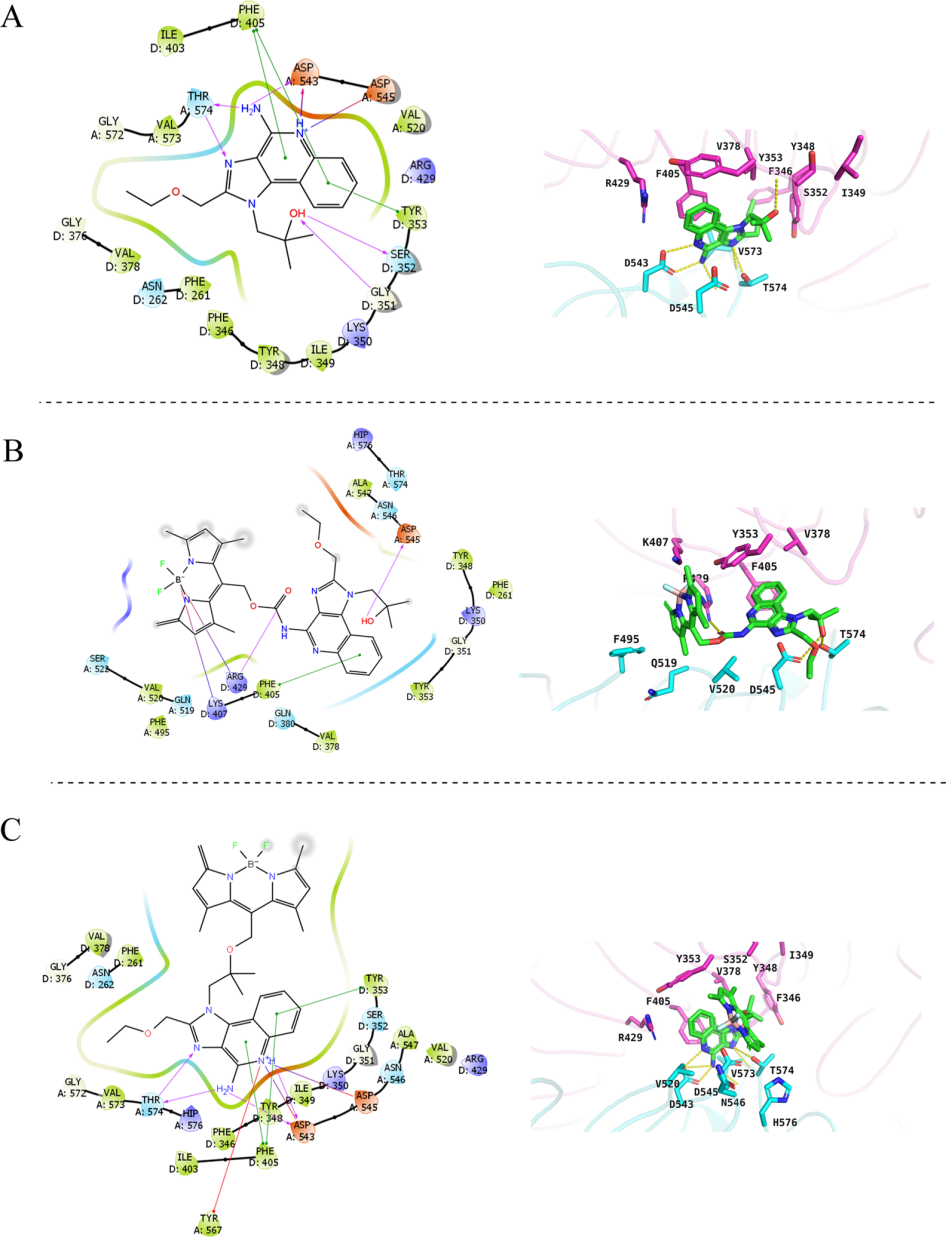
(A–C) show 2D and 3D binding interaction diagrams of resiquimod, compound **Ra**, and compound **Ra′** with TLR8 (PDB ID: 3W3L), respectively.

Additionally, the amidine group of the quinoline moiety formed hydrogen bonds with ASP^543^, ASP^545^, and THR^574^. The nitrogen atoms of the imidazole moiety formed hydrogen bonds with Thr^574^. The 2-ethoxymethyl substituent protruded into a small hydrophobic pocket formed by Phe^346^, Tyr^348^, Gly^376^, Val^378^, Ile^403^, Phe^405^, Gly^572^, and Val^573^ (Figure 2A). These hydrophobic interactions may be necessary for the agonistic activity of chemical ligands targeting TLR8.^29^ From the docking results, OH-caged resiquimod (**Ra**′**–Rh**′) showed higher binding affinities compared to NH_2_-caged compounds (**Ra–Rh**). The docking studies for caged resiquimods with various PPGs showed consistent results across different PPGs; however, BODIPY-caged resiquimods displayed either the highest binding energy or no binding at all. Compounds **Ra–Rh** caged at the C4 amine of imidazoquinolines demonstrated significantly lower binding affinities compared to resiquimod, as evidenced in the 2D binding diagrams, which showed no binding interactions with ASP^545^, ASP^543^, and THR^574^ (Figure 2B). In contrast, **Ra′–Rh′** compounds showed a trivial difference in binding affinities compared to resiquimod, with critical moieties still binding to ASP^545^, ASP^543^, and THR^574^ (Figure 2C). Additionally, the 2-ethoxymethyl substituent still protruded into a small hydrophobic pocket formed by Phe^346^, Tyr^348^, Gly^376^, Val^378^, Ile^403^, Phe^405^, Gly^572^, and Val^573^. These findings underscore the critical role of the resiquimod’s NH_2_ group in binding with TLR7/8. Based on that, it is predicted that the caging of resiquimod from the amine moiety would block the resiquimod activity.

Since the cargo release from BODIPY proceeds via a Photo-S_N_1 mechanism, which is highly dependent on the p*K*a of the cargo, amines generally serve as poor leaving groups.^24^ To address this limitation, a carbamate linker was employed to connect the C4 amine of resiquimod to the BODIPY chromophore. This modification would facilitate the deprotection reaction, enabling efficient photocleavage and ensuring the rapid release of resiquimod upon light activation. Additionally, red-light-sensitive BODIPY would be obtained by attaching two styryl groups on the 3 and 5 positions. It was reported that the extension of π-conjugation shifts the absorption maxima to the red-light region.^30^ Moreover, because the balance between lipid and water solubility is critical for any drug, two PEG (poly(ethylene glycol)) tails will be attached to the two styryls to enhance the aqueous solubility.

### 2.2 Synthesis of Photocaged Resiquimod

**Scheme 1:**
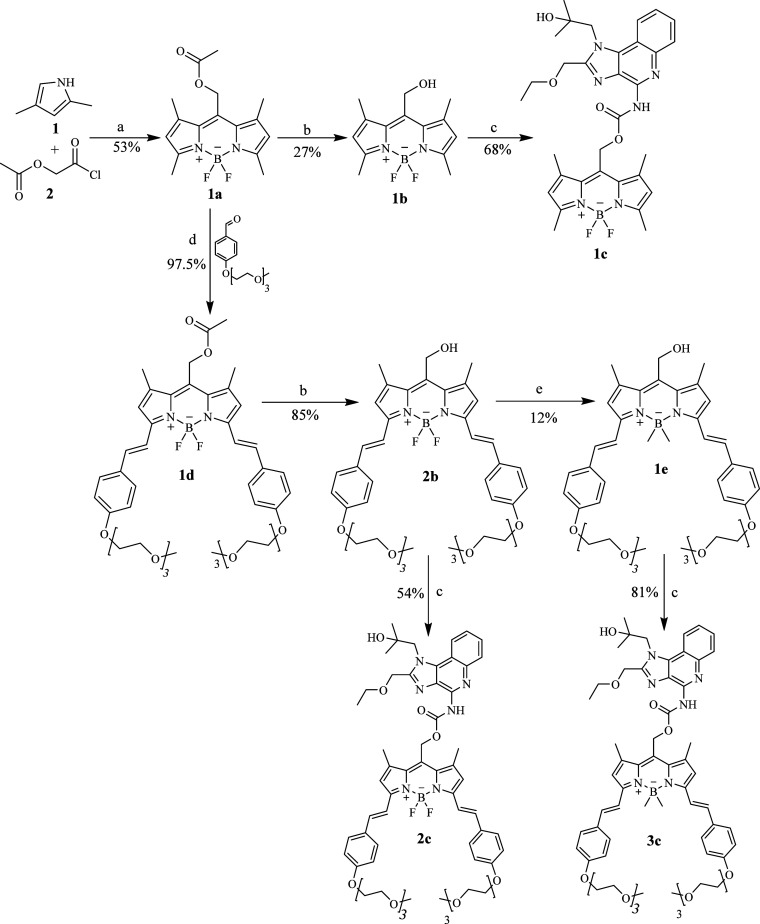
Synthetic Route for Compounds **1–3c** Reagents and conditions: (a) reflux for 1 h at 40 °C; DIPEA, BF_3_·OEt_2_, at room temperature for 30 min; (b) 0.1 M LiOH treatment; (c) resiquimod, 4-nitrophenyl chloroformate, DIPEA, reflux for 24 h at 40 °C; (d) piperidine, under vacuum for 3.5 h at 60 °C; (e) CH_3_MgBr, at room temperature.

The synthesis of the BODIPY core was accomplished in two steps, as illustrated in Scheme 1. The first step involved the preparation of dipyrromethene, followed by complexation with a boron atom. Compound **1a** (BODIPY acetate) was typically synthesized via a one-pot reaction. Initially, the acetyl dipyrromethene core was formed by condensing 2,4-dimethyl pyrrole (**1**) with acetoxyacetyl chloride (**2**) at 40 °C for 1 h. Subsequently, *N*,*N*-diisopropylethylamine (DIPEA) was added to neutralize the liberated acid. Finally, boron trifluoride etherate (BF_3_OEt_2_) was introduced to form compound **1a** (BODIPY acetate) through complexation between BF_3_ (a Lewis acid) and the nitrogen atoms of dipyrromethene (a Lewis base).

Separately, compound **2e** (4-[2-[2-(2-methoxyethoxy)ethoxy]ethoxy]benzaldehyde) was synthesized using the Williamson ether synthesis method, involving the reaction of *p*-hydroxybenzaldehyde and bromoether [1-bromo-2-(2-(2-methoxyethoxy)ethoxy)ethane] in the presence of K_2_CO_3_. The BODIPY core was elongated by introducing three-unit pegylated styryl groups through a Knoevenagel condensation reaction. This was achieved by reacting compound **2e** with compound **1a** at the 3 and 5 positions in the presence of piperidine under vacuum at 60 °C, resulting in the formation of compound **1d**.

BODIPY chromophores (**1b–2b**) were synthesized through the hydrolysis of **1a** and **1d** by using 0.1 M LiOH. A significant difference in yield was observed between the two reactions: **2b** was obtained with an 85% yield, whereas the yield of **1b** was only 27%, likely due to the steric protection afforded by the pegylated styryl groups in **2b**. Conversely, the boron atom of the BODIPY core in **1b** was more susceptible to attack by LiOH. Compound **1e** was obtained through methylation of the boron atom in **2b** by the addition of Grignard’s reagent (CH_3_MgBr).

The three BODIPY-caged resiquimod compounds (**1–3c**) were synthesized via a one-pot reaction involving BODIPY alcohols (**1b**, **2b**, and **1e**), resiquimod (as the cargo), and 4-nitrophenyl chloroformate (as the carbonyl group donor) in the presence of DIPEA. It was found that adding 4-nitrophenyl chloroformate at the beginning of the reaction significantly increased the yield compared to adding it with BODIPY alcohol or resiquimod alone to form a chloroformate intermediate. The yields of the three caged compounds **1c**, **2c**, and **3c** were 68%, 54%, and 81%, respectively.

### 2.3 Photophysical Properties and Photochemistry

**Figure 3:**
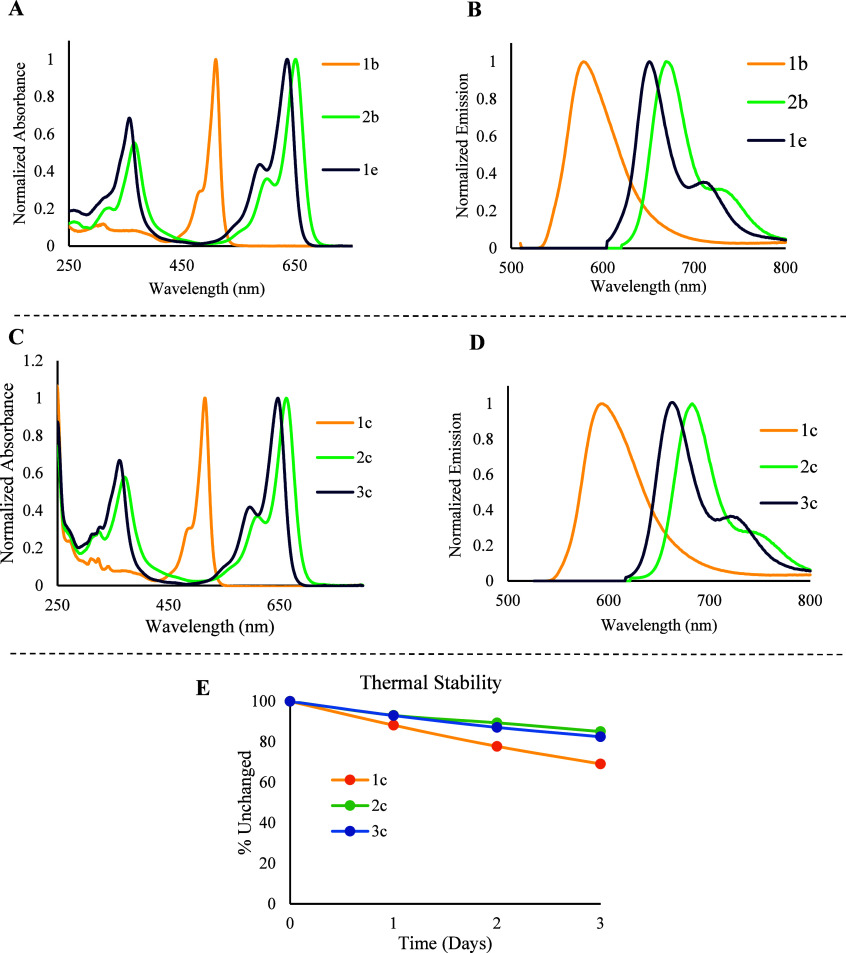
(A) UV–vis absorption spectra of compounds **1–2b** and **1e**; (B) fluorescence emission spectra of compounds **1–2b** and **1e**; (C) UV–vis absorption spectra of compounds **1–3c**; (D) fluorescence emission spectra of compounds **1–3c**, all recorded in CH_3_CN; (E) thermal stability analysis of compounds **1–3c** at 37.5 °C, conducted in DMSO-*d*_6_ and monitored via ^1^H NMR spectroscopy.

The UV–vis absorption and fluorescence emission spectra of BODIPY chromophores **1–2b** and **1e** and BODIPY-caged resiquimods (**1–3c**) were investigated in acetonitrile at room temperature, and all relevant data are summarized in Table 2. For BODIPY chromophores, **1b** is absorbed in the green-light region with an absorption maximum (λ_max_) of 510 nm. The extension of the π-conjugation system by attaching two styryl groups at positions 3 and 5 of the BODIPY core significantly shifted the λ_max_ of **2b** and **1e** to the red-light region. The absorption maximum of **2b** was observed at 651 nm, while the methylation of the boron atom slightly caused a blue shift, as noticed from **1e** with λ_max_ = 637 nm (Figure 3A). Similarly, BODIPY-caged resiquimods (**1–3c**) exhibited λ_max_ values of 516, 662, and 647 nm, respectively (Figure 3C). Analogously, the emission maxima for **1b** and **1c** were in the green-light region, 587 and 594 nm, respectively. This maximum red-shifted in **2b**, **1e**, **2c**, and **3c** to 670, 654, 683, and 663 nm, respectively, because of the two attached styryls (Figure 3B,D). Stokes shift is the wavelength difference in absorbed and emitted wavelengths of a fluorophore.^31^**1b** and **1c** showed a considerable value for the Stokes shift with about an 80 nm difference between the emitted and absorbed light. Meanwhile, **2b**, **1e**, **2c**, and **3c** showed similar stroke shift values of about 15–20 nm. This value indicates that the energy difference between the excited and ground states in **1b** and **1c** is much higher than those in **2b**, **1e**, **2c**, **and 3c**.

**Table 2:** Photophysical Data of **1b**, **2b**, **1e**, **1c**, **2c**, and **3c**

Cpd	λ_abs_^a^ (nm)	λ_emi_^b^ (nm)	SS^c^ (nm)	ε^d^ (M^–1^ cm^–1^)	Φ_f_^e^	τ^f^ (ns)	Φ_u_^g^ (%)
**1b**	510	587	77	75,950 ± 1300	0.835 ± 0.001	6.04 ± 0.01	nd^h^
**2b**	651	670	19	113,546 ± 215	0.554 ± 0.0005	3.83 ± 0.01	nd^h^
**3e**	637	654	17	124,169 ± 2836	0.479 ± 0.0005	3.39 ± 0.01	nd^h^
**1c**	516	594	78	74,250 ± 400	0.809 ± 0.0005	6.33 ± 0.003	1.17 × 10^–1^
**2c**	662	683	21	122,412 ± 1670	0.482 ± 0.0007	3.56 ± 0.08	1.18 × 10^–3^
**3c**	647	663	16	91,756 ± 6195	0.411 ± 0.0006	3.16 ± 0.01	2.09 × 10^–2^

a Absorption maximum.

b Fluorescence maximum.

c Stokes shift.

d Molar extinction coefficient.

e Fluorescence quantum yield, measured using an absolute PL quantum yield spectrometer.

f Fluorescence lifetime.

g Degradation quantum yield based on 10% conversion.

h Not detected, all measured in CH_3_CN.

The fluorescence lifetime profiles of BODIPY chromophores **1–2b** and **1e** and BODIPY-caged resiquimods **1–3c** were compared. Overall, the fluorescence quantum yields of the three chromophores **1b**, **2b**, and **1e** are more significant than those of their corresponding caged compounds **1**–**3c**. This means caging reduces the emissive character of the BODIPY fluorophore. **1b** and **1c** showed a much higher fluorescence quantum yield (about 0.8) than the other compounds, which showed about (0.4–0.5) values. This indicates that the radiative decay in **1b** and **1c** is much higher than in other compounds. The fluorescence lifetime indicates the time that a molecule remains in its excited state before returning to the ground state.^32,33^ Compounds **1b** and **1c** showed longer lifetimes of about 6 ns. Other compounds showed a faster decay rate of the excited state, and they had almost half of this lifetime with about 3 ns. Fluorescence lifetimes are measured using a time-correlated single-photon counting technique.

The degradation quantum yield of **1–3c** was determined in CH_3_CN by using potassium ferrioxalate as the actinometer.^34^ The samples were irradiated with a 365 nm LED lamp under air conditions. Before that, the photon flux was calculated (Figure S1). The decomposition quantum yield was calculated based on a 10% conversion to avoid interference with other photoproducts (the internal filter effect). Figure S2A–C shows the rate of 10% conversion of **1–3c**, respectively. Compound **1c** exhibited the highest Φ_u_ value among the three, with 0.12%. However, the BODIPY elongation with the two styryls significantly declined the quantum yield by 100 times, where the compound **2c** quantum yield was calculated to be 0.12 × 10^–2^%· Methylation of the boron atom of **2c** substantially increased the quantum yield by ∼17 times, where the Φ_u_ for **3c** was calculated to be 0.021%. The observed outcome aligns with previous reports indicating that the B-alkylation of BODIPY enhances photoreaction efficiency. This enhancement is attributed to the increased electron density contributed by the alkyl groups, which in turn stabilizes the intermediate carbocation. Furthermore, methyl groups have been identified as the most favorable substituents as extending the alkyl chain to ethyl or phenyl results in a reduced quantum yield. This decrease is likely due to a distortion in the planarity of the BODIPY core.^30,35,36^

### 2.4 Thermal Stability of Compounds **1–3c**

Thermal stability of the three caged resiquimods, **1**–**3c**, was investigated in DMSO-*d*_6_ at 37.5 °C in dark conditions. ^1^H NMR for the three compounds was measured periodically for 3 days. The decomposition was calculated based on the intensity of three different signals relative to the DMSO-*d*_6_ signal as an internal standard. The three signals were chosen as follows: one from the resiquimod part, one from the BODIPY part, and the NH signal as a linkage part (Figures S3–S5). Although the decomposition for the three peaks showed insignificant variations, the average mean was calculated. The three compounds showed no observable decomposition before 24 h. However, compound **1c** was the most thermally unstable of the three compounds. It exhibits 12% decomposition after 1 day and 22% and 31% after two and 3 days, respectively. Meanwhile, **2c** and **3c** showed almost the same thermal stability, with 7%, 11%, and 15% decomposition for the first, second, and third days, respectively (Figure 3E).

### 2.5 Resiquimod Uncaging from **1c**

**Figure 4:**
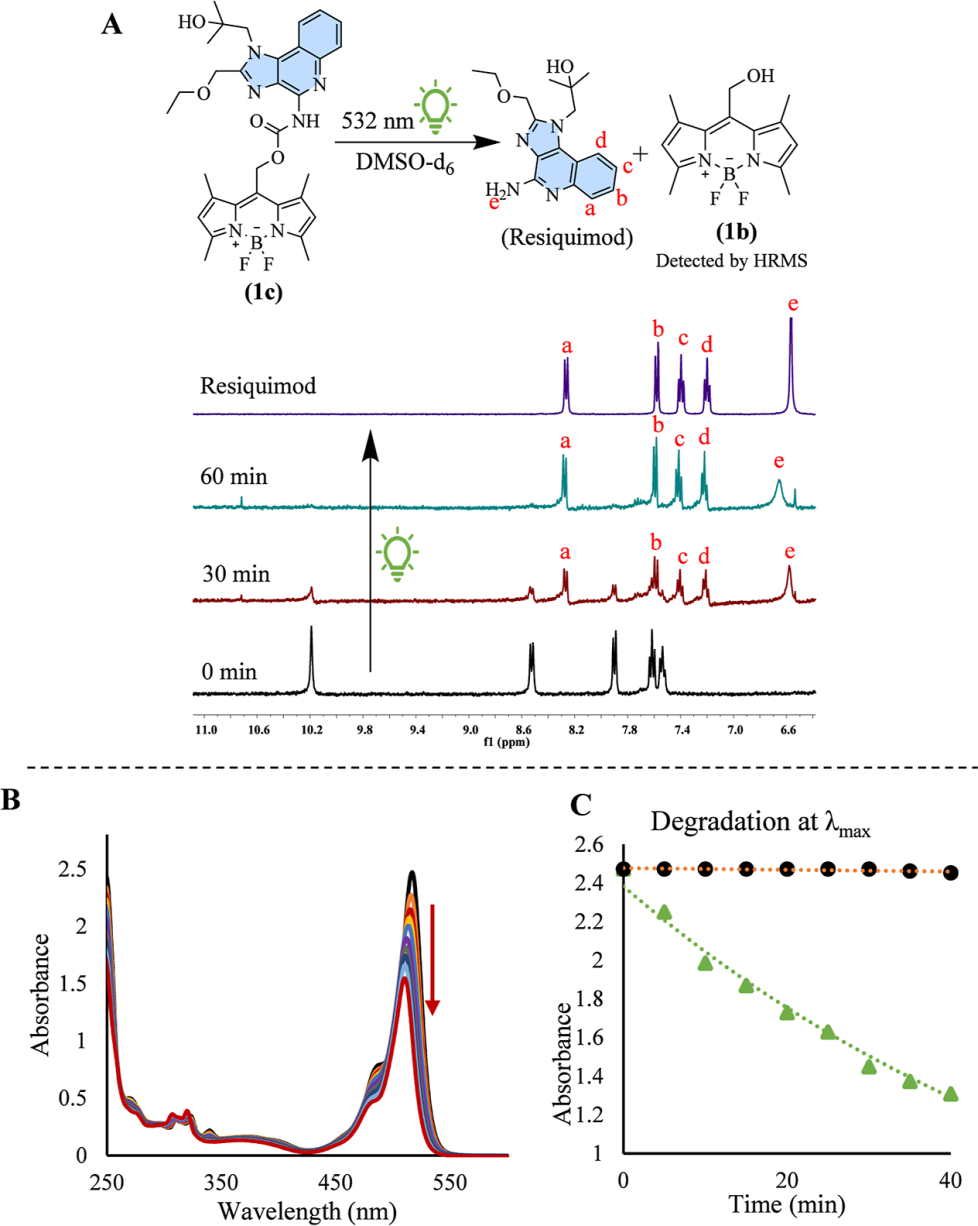
(A) ^1^H NMR spectra (400 MHz, 6.6–10.6 ppm) acquired during the photolysis of compound **1c** using a 532 nm using 10 Hz Na^+3^ YAG laser in DMSO-*d*_6_. The spectra are presented sequentially, illustrating compound **1c** prior to irradiation, after 30 min of irradiation, after 1 h of irradiation, and the ^1^H NMR spectrum of resiquimod in DMSO-*d*_6_. (B) Photolysis of compound **1c** in 50% water and 50% CH_3_CN, monitored at 5 min intervals. (C) Revealing the temporal decline at 516 nm of irradiated **1c** (green) and the aqueous stability of unirradiated **1c** (black).

The photouncaging of resiquimod from **1c** was investigated in DMSO-*d*_6_ using a 532 nm YAG Laser with 10 Hz Na^+3^ as a light source. A 0.4 mL aliquot of 8.49 mM **1c** solution was transferred to a NMR tube and irradiated using a laser with an approximate power output of ∼300 mW. The progress of the photoreaction was monitored via ^1^H NMR spectroscopy. As shown in Figure 4A, nearly complete consumption (∼100%) of **1c** was achieved after 1 h of irradiation, as evidenced by the disappearance of its characteristic aromatic signals. The uncaging of resiquimod was confirmed by the appearance of its diagnostic aromatic signals, including two doublets (a, b) and two triplets (c, d), which corresponded well with the ^1^H NMR spectrum of a resiquimod control sample. Following irradiation, triphenylmethane was added as an internal standard to the crude photolysate for quantification, revealing that approximately ∼95% of resiquimod had been released. Additionally, HRMS analysis of the photolysate confirmed the release of resiquimod and the formation of compound **1b** (Figure S6). However, detecting compound **1b** in the ^1^H NMR spectrum was challenging, likely due to further photosensitization effects in the presence of molecular oxygen.

To investigate the photorelease of resiquimod from compound **1c** in an aqueous medium, a solvent system comprising 50% water and 50% acetonitrile (CH_3_CN) was employed. The photolysis of **1c** was monitored by using UV–vis spectroscopy. A 3 mL aliquot of a 30 μM solution of **1c** was placed in a cuvette and irradiated with a 525 nm LED lamp, delivering a power of approximately 500 mW. Figure 4B demonstrates the gradual decrease in the λ_max_ of **1c** as a function of the irradiation time. This trend is further highlighted in Figure 4C, where the green markers represent a time-dependent reduction in absorbance at 516 nm, in contrast with the black markers, which indicate the dark control. The dark control exhibited no significant change over time, confirming the aqueous stability of **1c** in the absence of light. Absorbance measurements were recorded at 5 min intervals throughout the experiment.

**Figure 5:**
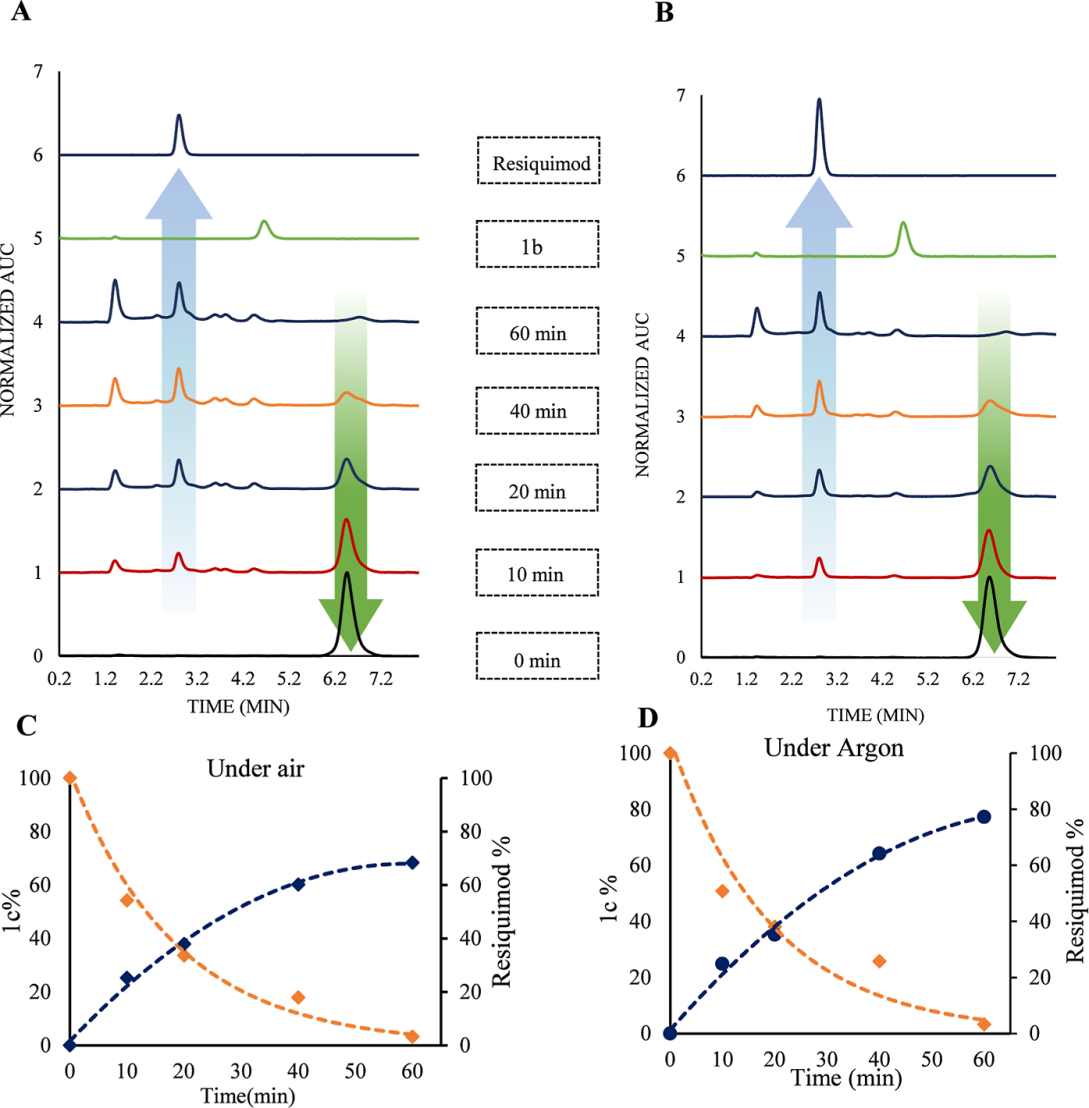
HPLC spectra depicting the photolysis of compound **1c** via a 532 nm laser lamp in CH_3_CN. (A) Irradiation performed under ambient air conditions. (B) Irradiation performed under an argon atmosphere. HPLC parameters include column type (ODS-3, 5 μm), flow rate (1.5 mL/min), detector wavelength (315 nm), and solvent system composition (CH_3_CN/H_2_O/triethylamine, 70:30:0.1%). Panels (C,D) feature dual *y*-axes, illustrating the percentage decline of compound **1c** during photolysis alongside the corresponding increase in the chemical yield of resiquimod over time, with calculations derived from the resiquimod calibration curve under both air and argon conditions.

The photorelease of resiquimod from **1c** was further investigated in CH_3_CN using high-performance liquid chromatography (HPLC). A 3 mL aliquot of a 0.55 mM solution of **1c** was placed in a cuvette, and two experimental setups were established to monitor the process. The first was irradiated under ambient air, while the second was deoxygenated by bubbling argon for 20 min before irradiation. The laser lamp was set to a power of ∼300 mW. Figure 5A,B illustrate the gradual decrease in the **1c** peak (retention time of 6.8 min) over time and the corresponding increase in the resiquimod peak (represented by a blue up-arrow) in both air and argon conditions, respectively. A minor peak, corresponding to BODIPY alcohol **1b**, was also observed at 4.15 min which matches with the **1b** spectrum. Additionally, a photoproduct with a retention time of 1.3 min showed a higher intensity (area under the curve) under air conditions than under argon, suggesting that oxygen promotes the formation of this species. Future efforts will focus on isolating and characterizing this photoproduct. To quantify the amount of released resiquimod, a resiquimod calibration curve was prepared (Figure S7), and the time-dependent changes in **1c** (orange line) and resiquimod (blue line) were plotted (Figure 5C,D). Based on a conversion rate of ∼97% in both cases, the chemical yield of resiquimod was calculated: ∼68% under air and ∼77% under argon. The lower yield in air can be attributed to quenching of the triplet state by oxygen, which does not occur under argon. The substantial difference in resiquimod yield between DMSO-*d*_6_ (95%) and CH_3_CN (68%) can be attributed to the higher water content in DMSO-*d*_6_, which enhances the capture of the meso-carbocation via solvolysis. This observation aligns with the photorelease mechanism proposed by Winter in 2015, wherein meso-BODIPY undergoes S_N_1 cleavage upon irradiation, resulting in the formation of a meso-carbocation through heterolysis, followed by solvolysis to complete the process.^37^

### 2.6 Resiquimod Uncaging from **2c**

The uncaging of resiquimod from compound **2c** was investigated in DMSO-*d*_6_ by using a 660 nm LED light source (∼400 mW). A 0.4 mL sample of 7.15 mM **2c** was irradiated and monitored via ^1^H NMR. Compared to compound **1c**, the release of resiquimod from **2c** proceeded at a significantly slower rate, with approximately 90% of **2c** consumed after 10 h of irradiation (Figure S8A). The release of resiquimod was confirmed by the appearance of characteristic aromatic signals (doublets a, b and triplets c, d), with minor peak shifts in the ^1^H NMR spectra. Although the ^1^H NMR spectrum of the photolysate shows a slight shift in arising peaks compared to resiquimod ^1^H NMR peaks, mass analysis confirmed the release of resiquimod and the formation of compound **2b**. In an aqueous solvent system (50:50 H_2_O/CH_3_CN), 30 μM **2c** was irradiated using a 660 nm LED (400 mW). The photolysis was tracked by monitoring the gradual decrease in λ_max_ of **2c** at 662 nm, indicating successful but very slow uncaging over approximately 200 min. The dark control exhibited no spectral changes, confirming the light-dependent nature of the reaction (Figure S8B).

### 2.7 Resiquimod Uncaging from **3c**

**Figure 6:**
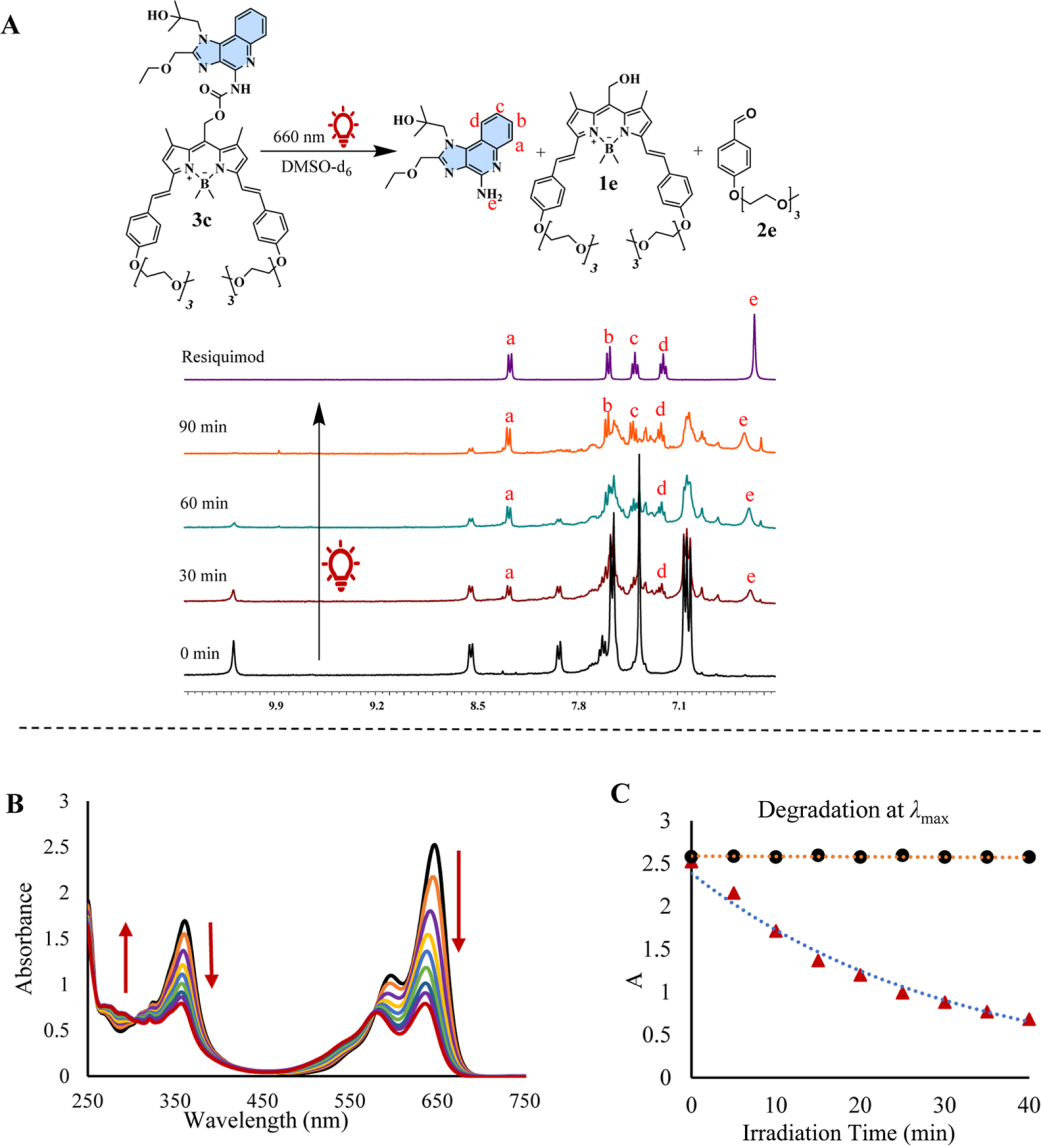
(A) ^1^H NMR spectra (400 MHz, 6.6–10.6 ppm) acquired during the photolysis of compound **3c** using a 660 nm LED lamp in DMSO-*d*_6_. The spectra are presented sequentially, illustrating compound **3c** prior to irradiation, after 30, 60, and 90 min of irradiation, and the ^1^H NMR spectrum of resiquimod in DMSO-*d*_6_. (B) Photolysis of compound **3c** in a solvent mixture of 50% acetonitrile and 50% water was monitored at 5 min intervals. (C) Temporal decline in the **3c** absorption maximum at 647 nm.

The photorelease of resiquimod from **3c** was investigated in DMSO-*d*_6_ using a 660 nm LED light source (∼400 mW). A 0.4 mL sample of 8.1 mM **3c** was irradiated and monitored by ^1^H NMR spectroscopy. Compared to **2c**, the uncaging process for **3c** was significantly faster and as clear as observed in **1c**. As depicted in the ^1^H NMR spectra (Figure 6A), approximately 90% of **3c** was consumed within 90 min of irradiation. The release of resiquimod was confirmed by the appearance of its characteristic aromatic signals in the ^1^H NMR spectrum, including two doublets (a, b) and two triplets (c, d), which aligned with the reference spectrum of resiquimod. This was further corroborated by HRMS, which confirmed the formation of resiquimod and compound **1e** in photolysate (Figure S9). Additionally, HRMS suggested the formation of **2e**, likely due to oxidation of the olefinic moiety of the styryl groups attached at the 3 or 5 positions of the BODIPY core. This oxidation was attributed to singlet oxygen (^1^O_2_) generated during the photoreaction, as BODIPY converts molecular oxygen into ^1^O_2_ (Figure S9).

The photouncaging of resiquimod from **3c** was also examined in aqueous media (50:50 H_2_O/CH_3_CN) using a 30 μM solution of **3c**. As shown in Figure 6B, the λ_max_ at 647 nm of **3c** decreased rapidly with irradiation, indicating a faster uncaging process compared to **2c**. Figure 6C displays the time-dependent decrease in the λ_max_ of **3c** (blue dotted line), while the dark control (black dotted line) exhibited no changes over time, confirming the aqueous stability of **3c** in the absence of light.

### 2.8 Safety Evaluation (Cytotoxicity on Normal Cells) and Cell Morphology after Light Irradiation

**Figure 7:**
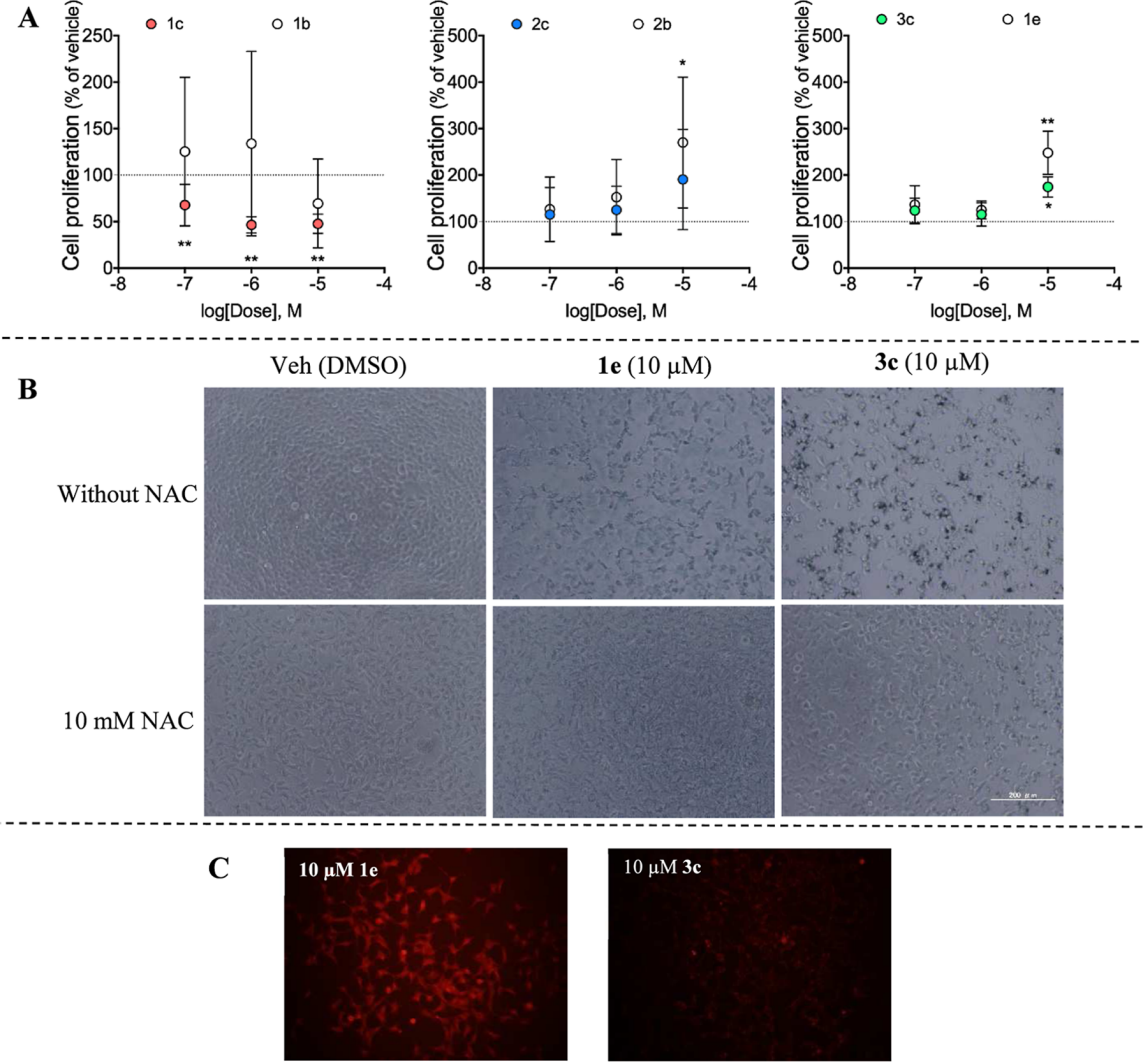
(A) Impact of compounds **1–3c** and their corresponding BODIPY photocages on the viability of AD293 cells in the absence of light irradiation. The data are presented as means ± SD of three different experiments. (B) Evaluation of the effects of compounds **3c** and **1e** on AD293 cell morphology after 30 min of irradiation with a 650 nm LED lamp, conducted in the presence or absence of *N*-acetyl cysteine. (C) Fluorescence imaging of AD293 cells treated with 10 μM **1e** or **3c**.

The cytotoxic potential of compounds **1–3c** and BODIPY photocages, each at concentrations 100 nM, 1 μM, and 10 μM, was assessed using the MTT assay (3-[4,5-dimethylthiazol-2-yl]-2,5-diphenyltetrazolium bromide) in AD293 cells. **1c** and **1b** were found to significantly inhibit cellular proliferation in comparison to the vehicle control (0.1% DMSO), indicating a notable degree of cytotoxicity. Conversely, compounds **2c**, **2b**, **3c**, and **1e** did not exhibit detectable cytotoxic effects under identical conditions at different concentrations (Figure 7A). Consequently, compound **3c** was selected for subsequent TLR7 receptor binding studies due to its noncytotoxic profile relative to **1c**, coupled with its superior photophysical properties, including a degradation quantum yield that was ∼17 times higher than that of compound **2c** and the faster resiquimod uncaging. The effects of compounds **3c** and **1e** on cellular morphology were further investigated by treating AD293 cells with 10 μM of each compound, followed by LED irradiation. In the absence of light exposure, neither compound induced noticeable morphological changes, consistent with the noncytotoxic profiles observed in the MTT assay (Figure S10). However, upon LED irradiation, significant morphological abnormalities indicative of cellular damage or apoptosis were observed, with compound **1e**, showing effects greater than those of **3c** (Figure 7B), likely due to singlet oxygen generation from the BODIPY core. The noncytotoxicity of the photoreaction side product **2e** (irradiation product of **3c**, Figure S11) supports this mechanism. Additionally, resiquimod (10 μM) induced no morphological changes under irradiated or nonirradiated conditions, further confirming that the Type II photodynamic activity of the BODIPY core is the primary driver of phototoxicity in compounds **1e** and **1c** (as will be elaborated in the photodynamic studies section). This hypothesis was substantiated through further morphological assessments of AD293 stably expressing TLR7. AD293 were seeded in 96-well plates at a density of 20,000 cells per well. After 24 h, the culture medium was replaced with Opti-MEM. One h later, cells were treated with 10 mM *N*-acetylcysteine. Thirty minutes post-treatment, cells were stimulated with 10 μM compounds **3c** or **1e** and subsequently exposed to LED light for 30 min. The control group was shielded from light exposure while undergoing identical experimental conditions. Cell observations were conducted 24 h post-treatment using a microscope. Pretreatment with NAC effectively abrogated the morphological alterations induced by **1e** and **3c**, thereby suggesting a pivotal role for reactive oxygen species (ROS) in driving these cellular changes (Figure 7B). These findings strongly imply that ROS are the critical mediators of the phototoxic effects observed in response to LED-activated **1e** and **3c**.

### 2.9 Bioimaging and TLR7 Assay of Choice

To assess the bioimaging capability of compound **1e**, cultured A549 cells were treated with 1 μM compound. Fluorescence derived from compound **1e** was detected at 0 and 40 min after treatment using a confocal microscope (FV3000, Olympus, Tokyo, Japan) equipped with a 640 nm laser and a 650–750 nm band-pass filter. The results demonstrated that compound **1e** successfully enters A549 cells, with a significant increase in intracellular fluorescence observed after 40 min (Figure S12). Compound **1e** also exhibited a strong bioimaging capability in AD293 cells, characterized by bright red fluorescence. In contrast, compound **3c** showed a weaker imaging performance (Figure 7C), although it still produced red fluorescence. This fluorescence, however, posed a challenge for TLR7 assays dependent on fluorescence detection. Specifically, the red fluorescence of both compounds **1e** and **3c** would interfere with NF-κB luciferase/fluorescence reporter assays, making them unsuitable for evaluating NF-κB signaling, leading to inaccurate results. To avoid this interference, we opted to assess the degradation of Iκ-B protein as an alternative indicator of NF-κB signaling activation. This approach would provide a reliable and interference-free method to evaluate TLR7-mediated NF-κB signaling in the presence of fluorescent compounds.

### 2.10 Spatiotemporal Regulation of TLR7 Receptors

**Figure 8:**
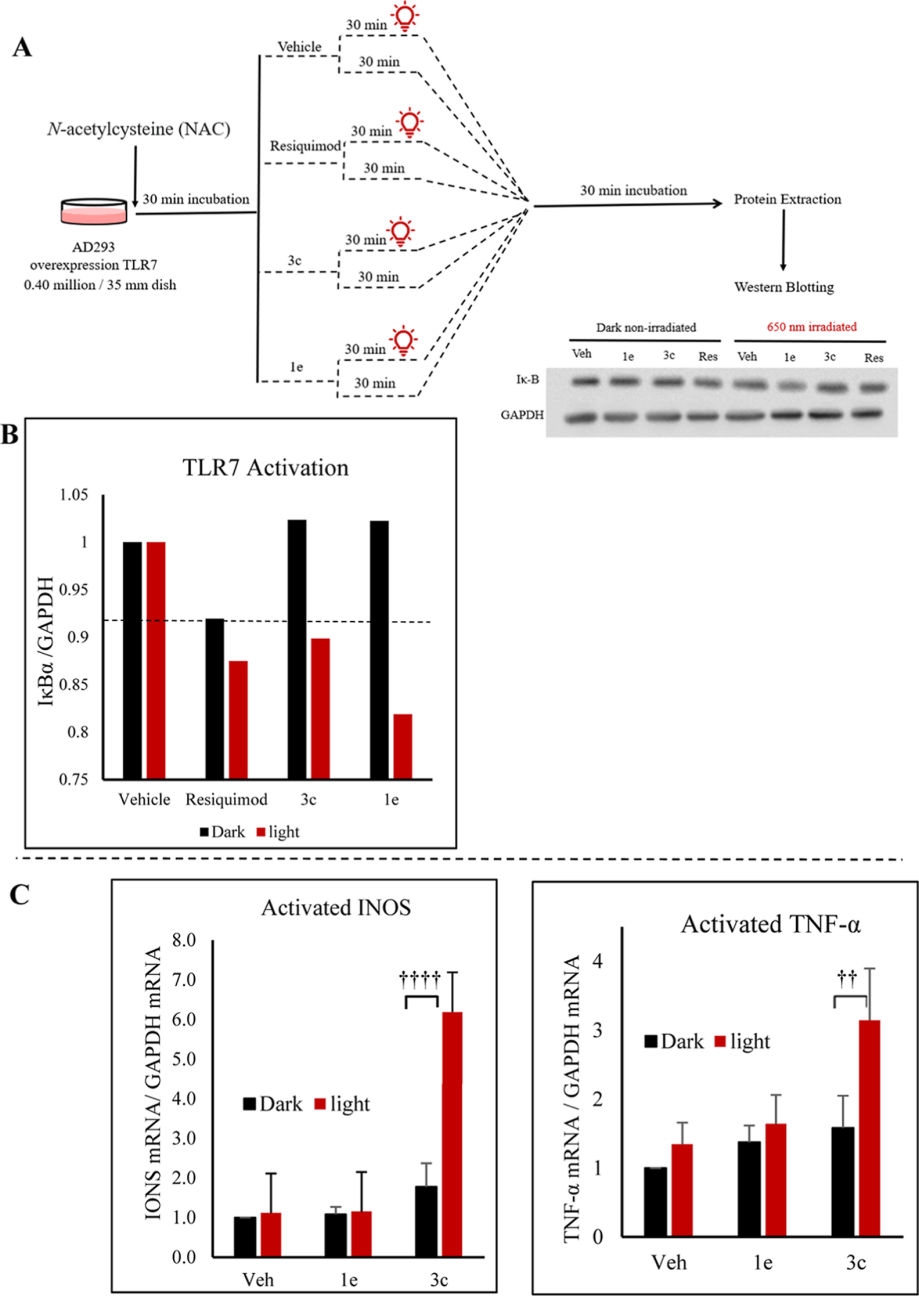
Spatiotemporal regulation of TLR7 receptors and immunotherapeutic potential of compound **3c**. (A) Schematic representation of the experimental setup and procedure. (B) Graphical representation of the relative IκBα/GAPDH protein levels for the vehicle, resiquimod, compounds **3c**, and **1e** under both dark conditions and 650 nm irradiation. The data are presented as means of three different experiments. (C) Red-light-activated immunotherapeutic potential of compound **3c**: induction of iNOS and TNF-α mRNA in M1 macrophage polarization. The data are presented as means ± SD. (†) Denotes fold changes in mRNA expression levels, representing the relative extent of regulation observed in treated samples.

To evaluate the efficacy of red light in controlling immune activation via compound **3c**, its effect on the NF-κB signaling pathway—a known downstream effector of TLR7—was analyzed using Western blotting. Stable AD293 cells expressing TLR7 were generated through transfection with a TLR7 plasmid followed by hygromycin B selection to ensure consistent expression. Prior to treatment, cells were preincubated with *N*-acetyl-cysteine (NAC) to neutralize reactive oxygen species, reducing nonspecific oxidative effects. After NAC pretreatment, cells were exposed to 10 μM either **3c** or **1e**, with or without 30 min of 650 nm red-light irradiation (Figure 8A). Western blot analysis monitored the degradation of IκBα, an inhibitor of NF-κB, as a measure of TLR7 activation. As expected, treatment with uncaged resiquimod (10 μM) resulted in IκBα degradation, confirming the activation of NF-κB and validating the experimental setup. In the absence of red-light irradiation, **3c**-treated cells exhibited stable IκBα levels, demonstrating that the photocaging strategy effectively suppressed resiquimod’s TLR7 activation. However, upon 650 nm red-light irradiation, **3c** successfully released resiquimod, resulting in significant degradation of IκBα, thereby activating the NF-κB pathway (Figure 8B). This demonstrates that the red-light-triggered uncaging of **3c** can precisely regulate TLR7 activation spatiotemporally. Unexpectedly, **1e**, primarily intended as a protecting group, showed pronounced activation of the NF-κB pathway upon red-light irradiation. This was attributed to the generation of singlet oxygen (^1^O_2_) by **1e** under LED irradiation, which appears to influence NF-κB signaling even in the presence of NAC, suggesting that the antioxidant capacity of NAC was insufficient to neutralize the oxidative effects. Previous studies indicate that reactive oxygen species (ROS) can modulate NF-κB signaling, either activating or inhibiting the pathway depending on the context and duration of exposure.^38,39^ The use of 650 nm red light in this system is advantageous compared to UV-based activation methods or the need for nanoparticle formulations as red light penetrates tissues more effectively and minimizes potential phototoxicity. These results highlight the potential of **3c** as a light-activated immune modulator while also raising intriguing questions about the role of photosensitizers like **1e** in immune signaling. Further studies are warranted to elucidate the precise molecular mechanisms by which ^1^O_2_ influences the NF-κB pathway and to explore its broader implications in immune regulation.

### 2.11 Immune Therapeutic Potential of **3c**

To evaluate the immunotherapeutic potential of compound **3c**, its ability to promote macrophage polarization toward the M1 phenotype was investigated by using the murine macrophage cell line RAW264.7. Cells were pretreated with *N*-acetyl-cysteine (NAC) to neutralize nonspecific reactive oxygen species, followed by treatment with 100 nM either **3c** or its precursor **1e**. Subsequently, the cells were irradiated with 650 nm red light for 30 min, and the expression of M1 macrophage markers was assessed. Following irradiation, cells treated with **3c** showed a ∼4-fold increase in inducible nitric oxide synthase (iNOS) and a ∼2-fold increase in tumor necrosis factor-alpha (TNFα) mRNA—key markers of M1 macrophage polarization—compared to the nonirradiated control (Figure 8C). In contrast, treatment with **1e** under the same conditions did not induce notable changes in the expression of these markers, indicating that the immunostimulatory effects are specifically triggered by the light-activated release of resiquimod from **3c**. Importantly, the use of 650 nm red light in this study offers significant advantages over UV-based activation methods commonly employed in previous studies.^9^ Red light penetrates tissues more effectively, minimizing damage to surrounding cells and tissues while achieving precise spatiotemporal control. Moreover, this approach eliminates the need for complex nanoparticle formulations to deliver resiquimod, simplifying the therapeutic strategy and enhancing its translational potential.^27^

### 2.12 Singlet Oxygen Quantum Yield

**Figure 9:**
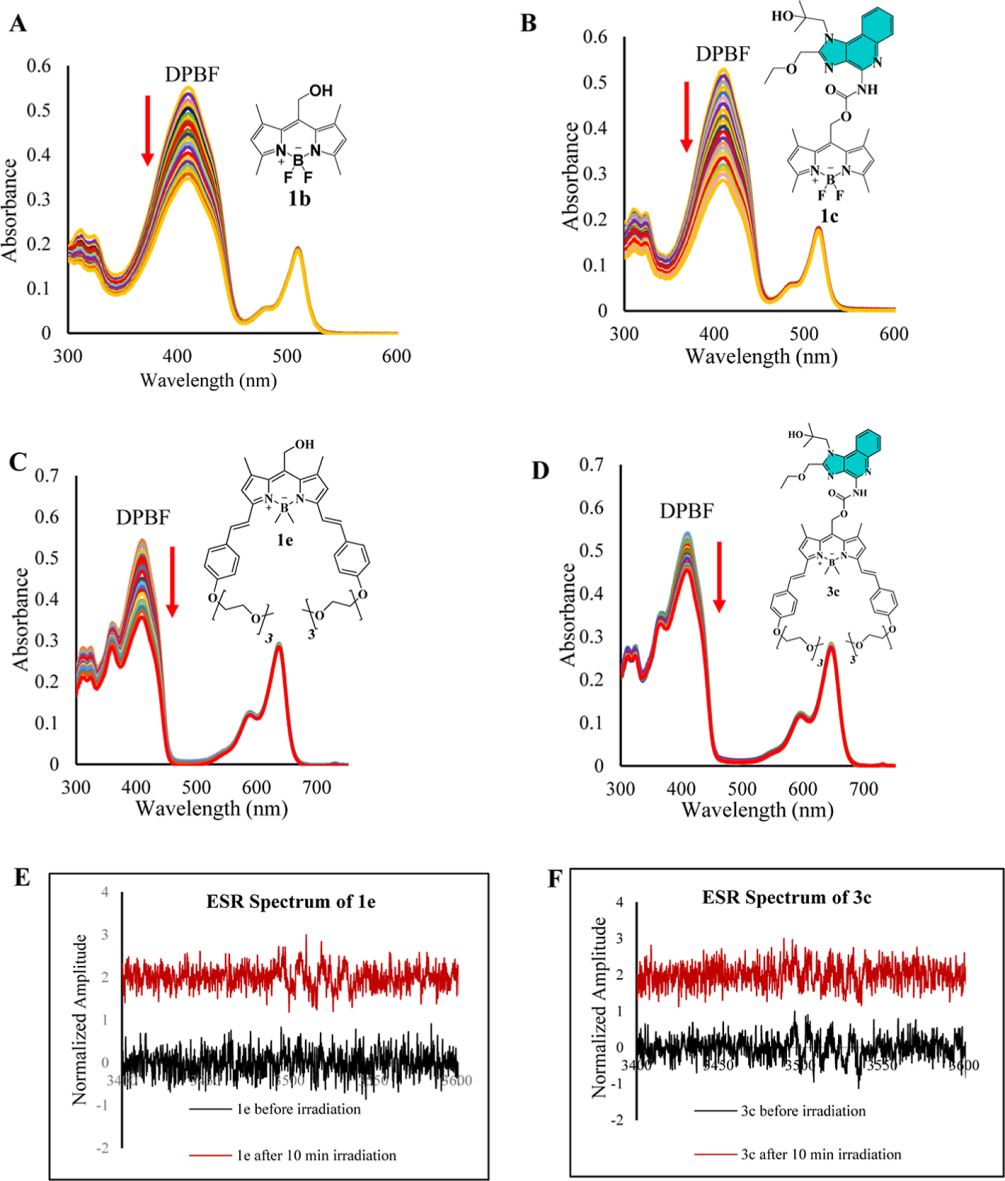
Measurement of singlet oxygen quantum yield, superoxide radical (O_2_^•–^), and hydroxyl radical (OH^•^) spin trapping. Panels (A,B) depict the alterations in the absorbance spectra of DPBF upon irradiation at 525 nm in the presence of compounds **1b** and **1c**, respectively. Similarly, panels (C,D) illustrate the spectral changes observed under irradiation at 660 nm in the presence of compounds **1e** and **3c**, respectively. Panels (E,F) present the ESR spectra for the detection of superoxide radicals (O_2_^•–^) and/or hydroxyl radical (OH^•^) before and after 10 min irradiation of compounds **1e** and **3c**, utilizing DMPO as the spin-trap agent.

The observed cytotoxicity in AD293 cells pretreated with **3c** and **1e** upon red light exposure in the absence of *N*-acetylcysteine (NAC), alongside the activation of the NF-κB pathway by **1e** in the presence of NAC, prompted an investigation into the singlet oxygen quantum yield (Φ_Δ_) of these compounds. Φ_Δ_ is a key parameter in evaluating the photosensitizing efficiency of photocages as it quantifies their ability to generate ^1^O_2_ upon irradiation. ^1^O_2_ generation potential of compounds **1–3c**, along with BODIPYs **1–2b** and **1e**, was assessed using 1,3-diphenylisobenzofuran (DPBF) as a selective ^1^O_2_ scavenger (Figure 9). Methylene blue was used as a reference for the Φ_Δ_ measurements of **2**–**3c**, **2b**, and **1e** because their absorption maxima are around 650 nm, similar to methylene blue (Figure S14). Rose bengal served as the reference for **1c** and **1b** because they are all absorbed in green light (Figure S13). The results revealed that free BODIPYs exhibited significantly higher singlet oxygen quantum yields compared to their corresponding photocaged resiquimod analogues. Specifically, **1b** and **1c** demonstrated Φ_Δ_ values of 7% and 3.2%, respectively, while **2b** and **2c** showed yields of 0.37% and 0.33%. Notably, methylation of the boron atom in the BODIPY core resulted in a 6-fold increase in Φ_Δ,_ as observed with **1e** and **3c**, which displayed Φ_Δ_ values of 2.2% and 1.2%, respectively. The lower singlet oxygen quantum yield of caged compounds **1–3c** compared to their respective photocages **1a**, **1b**, and **1e** can be attributed to the structural modification at the meso position, where resiquimod is caged. This modification alters the photophysical behavior of the BODIPY core. Specifically, the uncaging process of resiquimod from BODIPY predominantly occurs from the first excited singlet state (S1). As a result, a significant portion of the energy from S1 is channeled into the uncaging reaction rather than intersystem crossing (ISC) to the triplet state. Consequently, the reduced efficiency of ISC leads to lower production of singlet oxygen in **1–3c** compared with their respective BODIPY alcohols. To further elucidate the potential generation of reactive oxygen species (ROS) other than singlet oxygen, electron spin resonance (ESR) measurements were performed using 5,5-dimethyl-1-pyrroline-*N*-oxide (DMPO) as a spin-trapping agent for superoxide anion (O_2_^•–^) and hydroxyl radical (OH^•^).^40,41^ The characteristic six-line and four-line ESR signals corresponding to the DMPO–O_2_^•–^ and DMPO–OH^•^ adducts were not detected for BODIPY alcohol (**1e**) or photocaged resiquimod (**3c**) under either dark or irradiated conditions (Figure 9E,F). These findings clearly indicate that neither compound generates superoxide anions or hydroxyl radicals upon light activation. This supports the conclusion that the photodynamic activity of both compounds operates exclusively through a Type II mechanism with singlet oxygen acting as the primary cytotoxic agent. Additionally, the absence of superoxide anion and hydroxyl radical (OH^•^) generation in case of **3c** indicates that resiquimod does not modulate the photodynamic behavior of the BODIPY core or shift its activity toward a type I mechanism.

### 2.13 Photodynamic Effect (Phototoxicity)

**Figure 10:**
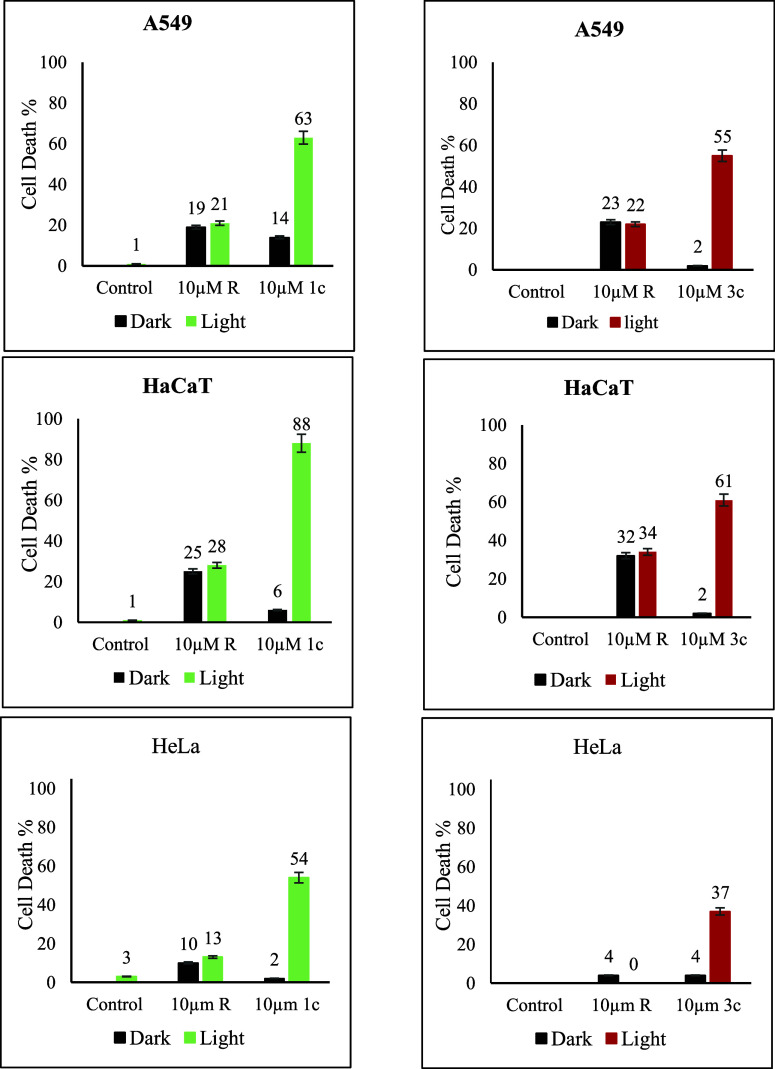
Effects of resiquimod (R), **1c**, and **3c** on cellular viability in the presence or absence of illumination. A549, HeLa, and HaCaT cells were seeded in 96-well plates, and resiquimod (R), **1c**, and **3c** at a concentration of 10 μM were added to each well after 1 day of cell incubation. Cells were maintained either in the dark (black bars), illuminated with 530 nm green light (green bars), or illuminated with 650 nm red light (red bars), and subsequently reincubated at 37 °C with 5% CO_2_ in a humidified atmosphere. Cell viability was assessed by CCK-8. The data are presented as means ± SD.

Building on the considerable singlet oxygen quantum yield results, we explored the photodynamic effects of BODIPY-caged resiquimods **1c** and **3c** using the CCK-8 assay to assess cytotoxicity under both light and dark conditions. In its uncaged form, resiquimod (10 μM) exhibited moderate cytotoxicity in HaCaT cells of ∼32% c, minimal cytotoxicity in A549 cells of ∼20%, and negligible cytotoxicity in HeLa cells. However, upon caging, the cytotoxicity of resiquimod on HaCaT was markedly reduced, indicating that the photocaging strategy effectively suppressed resiquimod’s inherent cytotoxicity. Under dark conditions, compound **1c** (10 μM) did not exhibit cytotoxicity in both HeLa and HaCaT cells and showed only ∼14% cell death in A549 cells. Similarly, **3c** (10 μM) showed negligible cytotoxicity across all cell lines tested, indicating that the photocaging strategy effectively minimizes both the intrinsic cytotoxicity of resiquimod on HaCaT cells and the possible dark toxicity associated with the BODIPY photosensitizer on all cell lines (Figure 10). Upon irradiation with green light (530 nm), a drastic increase in cytotoxicity was observed, demonstrating the photodynamic potential of the BODIPY-caged compounds. **1c**, in particular, showed significant phototoxicity in the three cell lines, with 63% cell death in A549 cells, 88% in HaCaT cells, and 54% in HeLa cells. Compound **3c** when irradiated with 650 nm, though less phototoxic than **1c**, still showed significant cytotoxic effects, inducing 55% cell death in A549 cells, 41% in HaCaT cells, and 37% in HeLa cells. The significant increase in cell death upon light activation is primarily attributed to the photodynamic effect of the BODIPY core, which is further synergized with resiquimod’s intrinsic cytotoxicity in irradiated HaCaT cells.

### 2.14 3D Tumor Spheroid Assessment of **3c**’s Safety and Efficacy

**Figure 11:**
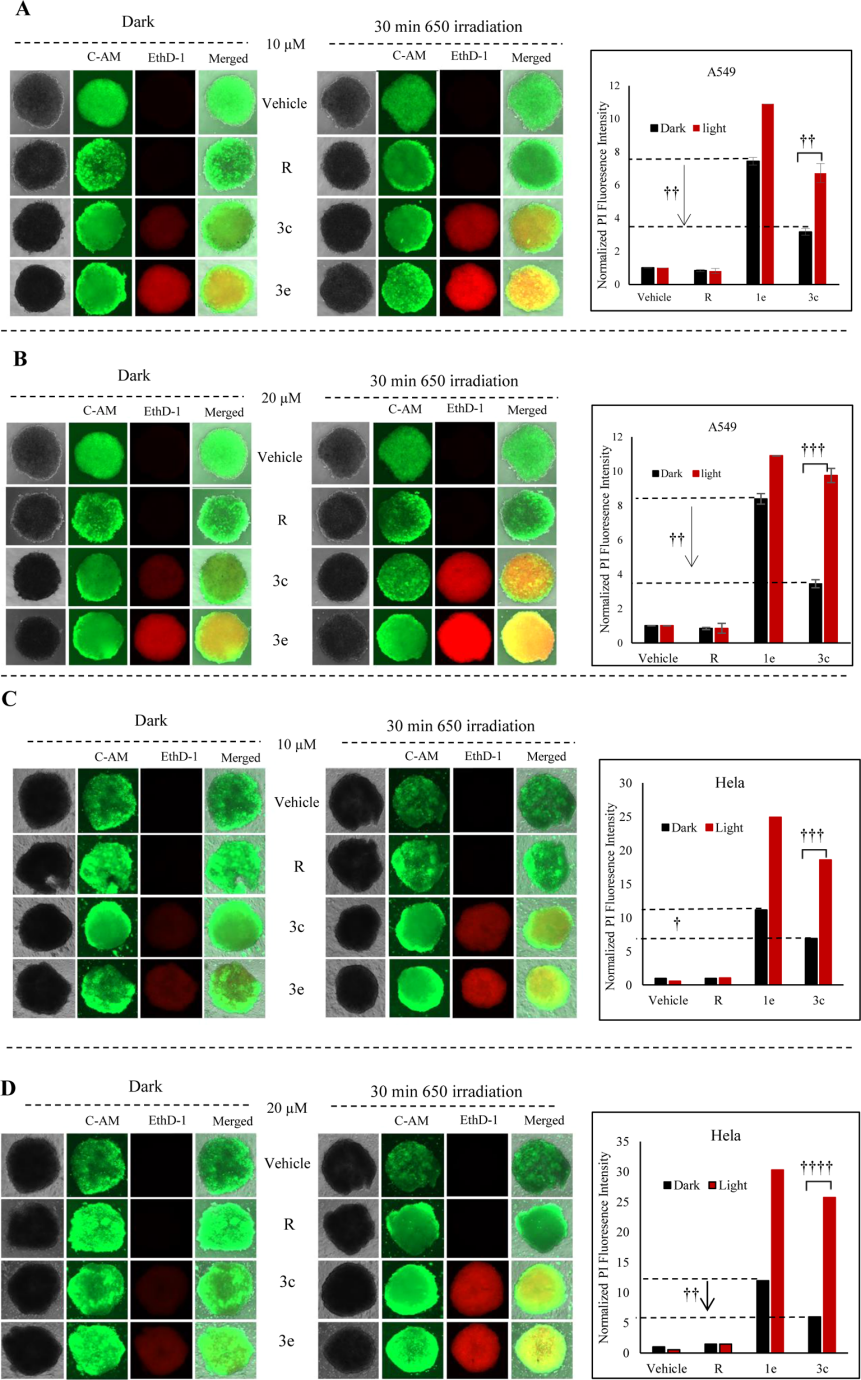
Photodynamic and dark toxicity evaluation in 3D tumor spheroids for compounds **3c** and **1e**. Live/dead cell assays were performed using calcein AM (C-AM, green) to label viable cells and ethidium homodimer-1 (EthD-1, red) to identify dead cells. (A,B) Photodynamic efficacy and dark toxicity were assessed in A549 spheroids treated with resiquimod (R), BODIPY alcohol (**1e**), and the BODIPY-resiquimod hybrid (**3c**) at concentrations of 10 μM and 20 μM under irradiated and nonirradiated conditions. (C,D) Photodynamic efficacy and dark toxicity in HeLa spheroids using the same compounds and experimental setup. The histograms accompanying panels (A–D) depict ethidium homodimer-1 fluorescence intensity as a quantitative measure for cell death. (†) Denotes fold changes in fluorescence intensity, representing the relative extent of cell death observed in treated spheroids. The data for A and B are presented as means ± SD.

To further evaluate the safety and therapeutic potential of the BODIPY-resiquimod hybrid (**3c**) in a more physiologically relevant model, we conducted a 3D tumor spheroid experiment using HeLa and A549 cells. This model allows for a more comprehensive assessment of the photodynamic effects and dark toxicity of **3c** in a 3D cellular context. In its uncaged form, resiquimod (10 μM and 20 μM) exhibited no detectable activity under either dark or light conditions, consistent with its minimal intrinsic cytotoxicity. In contrast, the BODIPY photosensitizer (**1e**) exhibited markedly higher dark toxicity compared to compound **3c**, demonstrating approximately 2-fold greater cytotoxicity in both cell lines at concentrations of 10 and 20 μM. These findings confirm that conjugating resiquimod to the BODIPY core effectively mitigates the inherent dark toxicity typically associated with photosensitizers when used at higher concentrations.^42^ Upon light irradiation, both the BODIPY alcohol (**1e**) and the BODIPY-resiquimod hybrid (**3c**) displayed substantial phototoxic effects compared to their nonirradiated counterparts, underscoring the robust photodynamic activity of these compounds. Notably, the BODIPY alcohol exhibited higher photodynamic activity than **3c**. However, the significantly reduced dark toxicity of **3c** underscores its advantage as a safer and more controlled therapeutic agent for photodynamic therapy (Figure 11). These results validate our hypothesis that modifying the BODIPY structure by attaching resiquimod to its hydroxyl group reduces the dark toxicity without significantly compromising photodynamic efficacy. This strategic design enhances the therapeutic profile of BODIPY-based photosensitizers, offering a promising approach for safer and more effective photodynamic therapy in complex biological systems.

## 3 Conclusions

In this study, we developed BODIPY-caged resiquimod compounds (**1–3c**) as dual-acting phototherapeutics, addressing the limitations of traditional UV-responsive PPGs. Molecular docking simulations guided the rational design, identifying the amine moiety of resiquimod as the optimal caging site for effective photocaging. Unlike prior UV-based methods, our BODIPY-caged resiquimod absorbs the red light, offering deeper tissue penetration and minimizing possible phototoxicity. Compound **3c** demonstrated strong thermal stability, noncytotoxicity in dark conditions, and precise spatiotemporal control over TLR7 activity. In the absence of light, **3c** effectively masked resiquimod’s activity, preventing NF-κB signaling pathway activation. However, upon red-light irradiation, resiquimod was released, restoring its immune-modulatory effects and activating the NF-κB pathway, thereby achieving targeted TLR7 activation. Macrophage activation assays confirmed the robust immunostimulatory effects of **3c** upon light-triggered uncaging, further supporting its immune therapeutic potential. Additionally, 3D spheroid experiments revealed that compound **3c** exhibited minimal dark toxicity compared to **1e** (BODIPY alcohol), indicating that attaching resiquimod to BODIPY effectively masked the photosensitizer’s dark toxicity. Upon red-light irradiation, compound **3c** demonstrated cytotoxicity comparable to that of the parent photosensitizer **1e**. These findings underscore a “win–win” strategy where BODIPY-caged resiquimod acts as both a light-induced immunostimulant and a noncytotoxic photosensitizer. These findings underscore the potential of BODIPY-caged resiquimod as a dual-function therapeutic with precise light-triggered immune modulation and photodynamic effects, offering a promising platform for cancer therapy that integrates phototherapy and immunotherapy.

## 4 Experimental Section

### 4.1 General Information

All commercially available chemical reagents were purchased from TCI, Wako, or Sigma-Aldrich and directly used without any further purification. NMR spectra were recorded on Bruker Ascend 400 (^1^H NMR: 400 MHz, ^13^C NMR: 100 Hz) spectrometers at 298 K. Coupling constants (*J*) are denoted in Hz and chemical shifts (δ) are in ppm. The abbreviations s, d, t, q, and dd denote the resonance multiplicities singlet, doublet, triplet, quartet, and double of doublets, respectively. Mass spectrometric data were observed with Thermo Fisher Scientific LTQ Orbitrap XL. UV–vis spectra were recorded on a SHIMADZU UV-3600 Plus spectrometer. The spectra were observed at room temperature using a slit width of 1 nm with middle scan rate. Fluorescence emission spectra were obtained from a FluoroMax-4 spectrofluorometer. Fluorescence quantum yield was determined using an Absolute PL quantum yield spectrometer C11347. Compounds **1–3c** were purified to a purity of over 95% as determined by HPLC (Figures S15–S17).

### 4.2 Molecular Docking

The molecular docking studies were conducted using Schrödinger software, version 2022.2, utilizing the Maestro GUI. The crystal structures of human TLR8 (PDB ID: 3W3L, resolution 2.33 Å)^29^ and monkey TLR7 (PDB ID: 5GMH, resolution 2.20 Å),^43^ both complexed with resiquimod (R848), were obtained from the Protein Data Bank (PDB). These structures were prepared using Maestro’s Protein Preparation Wizard, which included the removal of nonessential water molecules, the addition of hydrogen atoms for correct protonation states and geometry optimization, and the optimization of the hydrogen bonding network. The OPLS4 force field was applied for accurate molecular mechanics calculations of the proteins.^44^ The synthesized compounds were prepared using the LigPrep module in Maestro. This process generated various protonation states at physiological pH (7.0 ± 2.0) and performed energy minimization to obtain the most stable conformations for docking. The OPLS4 force field was also used for the compounds to ensure consistency in the molecular mechanics calculations. Receptor grids were created around the active sites of TLR8 and TLR7. For TLR8, the grid was centered at coordinates *x*: 51.53, *y*: 15.57, *z*: 23.38, and for TLR7, at coordinates *x*: −14.92, *y*: −28.43, *z*: −11.98, both with dimensions of 20 Å × 20 Å × 20 Å. The centroids were based on the cocrystallized resiquimod’s position to include all critical interacting residues and allow for conformational flexibility. The compounds were docked into the target proteins using the Glide module in standard precision (SP) mode to achieve accurate binding affinity predictions.^45^ The Glide Score estimated the free energy of binding, aiding in the identification of favorable compound–protein interactions. The docking protocol was validated by redocking the cocrystallized resiquimod and comparing the predicted poses with the experimental structures. For TLR8, the root-mean-square deviation (RMSD) was 0.69 Å and, for TLR7, it was 0.74 Å. An RMSD of less than 2 Å indicated a successful docking protocol, demonstrating the accuracy and reliability of the studies.^46^

### 4.3 Synthesis of Compound **1a**

Under dark conditions, in a 2-necked round-bottom flask, 2.854 g (30 mmol) of 2,4-dimethyl pyrrole (**1**) was dissolved in dry dichloromethane (30 mL) under a nitrogen atmosphere. Acetoxyacetyl chloride (**2**) 2.475 g (18 mmol) was added dropwise to the solution, and the mixture was stirred at room temperature for 10 min. Subsequently, the solution was refluxed at 40 °C for 1 h. Afterward, the solution was cooled to room temperature, and 7.755 g (60 mmol) of *N*,*N*-diisopropylethylamine (DIPEA) was added dropwise. The mixture was stirred for 30 min before adding 5.677 g (40 mmol) of BF_3_OEt_2_ dropwise. The resulting mixture was stirred for an additional 30 min. The mixture evaporated, leaving behind a residue. The residue was then purified on a silica column using a hexane/dichloromethane mixture (4:3). This yielded 2.548 g (53%) of **1a** as a shiny orange solid. ^1^H NMR (400 MHz, CDCl_3_, δ): 6.08 (s, 2H), 5.30 (s, 2H), 2.53 (s, 6H), 2.36 (s, 6H), 2.13 (s, 3H). ^13^C NMR (100 MHz, CDCl_3_): δ 170.50, 156.61, 141.47, 133.36, 132.66, 122.29, 57.85, 20.54, 15.57, 14.64. HRMS-ESI calcd for C_17_H_20_BF_2_N_2_O_2_ [M + H]^+^ 321.15804; found, 321.15781.

### 4.4 Synthesis of Compound **1b**

In a round-bottom flask, 1.68 g (5.25 mmol) of (**1a**) was dissolved in 100 mL of tetrahydrofuran at room temperature in dark conditions. Then, 100 mL of 0.1 M aqueous LiOH solution was added dropwise. The mixture was stirred at room temperature for 30 min, extracted directly in dichloromethane, and then washed two times with water. The organic layer was dried by using anhydrous Na_2_SO_4_ to remove water droplets. DCM evaporated at room temperature, and the crude was loaded on a silica column. The red solid **1b** was separated using dichloromethane as an eluent to yield 393.75 mg (27%). ^1^H NMR (400 MHz, DMSO, δ): 6.29 (s, 2H), 5.61 (t, *J* = 5.1 Hz, 1H), 4.77 (d, *J* = 5.0 Hz, 2H), 2.55 (s, 6H), 2.47 (s, 6H). ^13^C NMR (100 MHz, DMSO-*d*_6_): δ 154.58, 142.20, 141.03, 131.85, 121.71, 54.18, 15.22, 14.31. HRMS-ESI calcd for C_14_H_18_BF_2_N_2_O [M + H] ^+^ 279.14748; found, 279.14767.

### 4.5 Synthesis of Compound **h**

Two g (16.43 mmol) of *P*-hydroxy benzaldehyde and 5 g (35.8 mmol) of K_2_CO_3_ were transferred to a 2-neck round-bottom flask, and the air was removed by repeated vacuum–nitrogen cycles. Afterward, 20 mL of DMF was added, and stirring was conducted to ensure a homogeneous suspension. 3.93 g (17.34 mmol) of bromoether [1-bromo-2-(2-(2-methoxyethoxy) ethoxy) ethane] was diluted with 10 mL of anhydrous DMF and injected into the homogeneous suspension at room temperature. The mixture was refluxed at 65 °C for 24 h. The reaction mixture was directly extracted by using biphasic water and ethyl acetate. The ethyl acetate layer was washed many times with water to remove all DMF. Before evaporation, the organic layer was dried over anhydrous sodium sulfate to obtain yield = 83% (3.6 g yellow oil). ^1^H NMR (400 MHz, CDCl_3_, δ): 9.84 (s, 1H), 7.82–7.72 (m, 2H), 6.98 (d, *J* = 8.6 Hz, 2H), 4.21–4.13 (m, 2H), 3.85 (dd, *J* = 5.1, 4.4 Hz, 2H), 3.74–3.67 (m, 2H), 3.66–3.57 (m, 4H), 3.50 (dd, *J* = 6.1, 3.4 Hz, 2H), 3.33 (d, *J* = 0.8 Hz, 3H). ^13^C NMR (100 MHz, CDCl_3_) δ: 190.45, 163.69, 131.68, 129.87, 114.73, 71.71, 70.64, 70.41, 70.32, 69.24, 67.64, 58.70. HRMS-ESI calcd for C_14_H_20_O_7_Na [M + Na] ^+^ 291.12029; found, 291.12091.

### 4.6 Synthesis of Compound **1d**

0.6 g (1.875 mmol) of **1a**, 2.9 g (18.75 mmol) of 4-(1,4,7,10-tetraocaundecyl) benzaldehyde, 1 mL of anhydrous DMF, and 12 drops of piperidine were added to a 2 neck round-bottom flask. Under dark conditions, the reaction mixture was stirred while vacuuming at 60 °C for 3.5 h until the reaction was complete by TLC. The desired compound was purified by silica gel flash chromatography using ethyl acetate/hexane (3:1) as primary eluents, and finally, the desired compound (**1d**) was eluted with 100% ethyl acetate to give 1.5 g (97.5% yield) as a dark-green semisolid. ^1^H NMR (400 MHz, CDCl_3_, δ): 7.52 (d, *J* = 13.6 Hz, 2H), 7.48 (d, *J* = 8.6 Hz, 2H), 7.15 (d, *J* = 16.2 Hz, 2H), 6.86 (d, *J* = 8.6 Hz, 4H), 6.63 (s, 2H), 5.19 (s, 2H), 4.15–4.05 (m, 4H), 3.85–3.76 (m, 4H), 3.72–3.66 (m, 4H), 3.66–3.63 (m, 4H), 3.64–3.58 (m, 28H), 3.56–3.46 (m, 41H), 3.33 (s, 6H), 2.30 (s, 6H), 2.07 (s, 3H).

### 4.7 Synthesis of Compound **2b**

In a round-bottom flask, 1.5 g (1.828 mmol) of **1d** was dissolved in 300 mL of dichloromethane and methanol (1:1). A 220 mL portion of 0.1 M LiOH was added dropwise while stirring. The mixture was stirred in dark conditions for 4 h until the starting material was consumed. An excess amount of dichloromethane was added, and the mixture was washed three times with brine. The organic layer was collected and dried over anhydrous MgSO_4_. The organic layer was evaporated, and the product was purified by GPC to obtain a dark blue solid in an 85% yield. ^1^H NMR (400 MHz, CDCl_3_, δ): 7.56–7.40 (m, 6H), 7.08 (d, *J* = 16.2 Hz, 2H), 6.86 (d, *J* = 8.6 Hz, 4H), 6.53 (s, 2H), 4.67 (s, 2H), 4.20–3.98 (t, 4H), 3.87–3.75 (t, 4H), 3.74–3.67 (m, 4H), 3.67–3.62 (m, 4H), 3.63–3.56 (m, 4H), 3.57–3.43 (m, 6H), 3.34 (s, 6H), 2.37 (s, 6H). ^13^C NMR (100 MHz, CDCl_3_): δ 159.61, 152.71, 140.25, 136.02, 134.63, 134.10, 129.60, 129.03, 118.25, 116.95, 114.87, 77.35, 71.90, 70.79, 70.60, 70.50, 69.64, 67.47, 58.99, 55.45, 15.58. HRMS-ESI calcd for C_42_H_53_BF_2_N_2_O_9_Na [M + Na] ^+^ 801.37044; found, 801.37085.

### 4.8 Synthesis of Compound **1e**

300 mg (0.38 mmol) of **2b** was transferred to a two-neck round-bottom flask, and air was removed by repeated vacuum–nitrogen cycles. Afterward, 40 mL of dry THF was injected under nitrogen conditions. 3.84 mL (3.8 mmol) of 1 M CH_3_MgBr in THF was injected dropwise at 0 C. The reaction mixture was stirred for 1 h at room temperature under dark. The reaction was quenched by dropwise addition of 20 mL of saturated NH_4_Cl and an excess of dichloromethane. The mixture was washed 3 times with brine. The organic layer was collected and dried over anhydrous MgSO_4_. The residue was then purified on a silica column using a gradient elution: first, 100% dichloromethane, dichloromethane, and ethyl acetate 80:20, 60:40, and (80:20), and finally with 100% ethyl acetate. The product was extra-purified using GPC to obtain 65 mg of a dark-blue solid in a 22% yield. ^1^H NMR (400 MHz, CDCl_3_, δ): 7.48 (d, *J* = 8.4 Hz, 4H), 7.44 (s, 2H), 7.05 (d, *J* = 16.2 Hz, 2H), 6.94 (d, *J* = 8.5 Hz, 4H), 6.71 (s, 2H), 5.29 (s, 1H), 4.98 (s, 2H), 4.24–4.10 (m, 4H), 3.93–3.82 (m, 4H), 3.75 (dd, *J* = 5.5, 3.4 Hz, 4H), 3.69 (dd, *J* = 5.8, 3.6 Hz, 4H), 3.68–3.61 (m, 4H), 3.60–3.51 (m, 4H), 3.38 (s, 6H), 2.58 (s, 6H), 0.44 (s, 6H). ^13^C NMR (100 MHz, CDCl_3_): δ 159.25, 150.43, 136.56, 135.68, 132.72, 132.54, 130.16, 128.36, 128.17, 119.20, 118.60, 115.05, 77.23, 71.95, 70.87, 70.67, 70.58, 69.70, 67.53, 59.05, 56.36, 31.92, 29.69, 29.35, 29.26, 22.68, 16.13, 14.11, 14.04. HRMS-ESI calcd for C_44_H_59_BN_2_O_9_Na [M + Na]^+^ 793.42058; found, 793.42084.

### 4.9 Synthesis of Compound **1c**

38 mg (0.136 mmol, 1 equiv) of **1b**, 43 mg (0.136 mmol, 1 equiv) of resiquimod, and 41 mg (0.2 mmol, 1.5 equiv) of 4-nitrophenyl chloroformate were transferred to a two-neck round-bottom flask, and the air was removed by repeated vacuum–nitrogen cycles. Afterward, 5 mL of dry THF was injected under nitrogen to dissolve the three compounds. 88 mg (0.68 mmol, 5 equiv) of DIPEA was injected dropwise at room temperature. The mixture was stirred for 10 min at room temperature before refluxing at 35–40 °C for 24 h in the dark. The reaction was quenched by the addition of an excess amount of dichloromethane. The organic layer was washed 3 times with water and dried over anhydrous MgSO_4_. The residue was then purified on a silica column using gradient elution: first, 100% dichloromethane and dichloromethane/ethyl acetate 10:1, 5:1, and finally 1:1. The product was crystallized using dichloromethane and hexane to obtain 58 mg of pure orange solid in a 68% yield. ^1^H NMR (400 MHz, CDCl_3_, δ): 10.75 (s, 1H), 8.22 (d, *J* = 8.1 Hz, 1H), 8.10 (d, *J* = 8.2 Hz, 1H), 7.65 (t, *J* = 7.5 Hz, 1H), 7.53 (t, *J* = 7.5 Hz, 1H), 5.78 (s, 2H), 5.18 (s, 2H), 4.66 (s, 2H), 4.43 (s, 1H), 4.36 (d, *J* = 55.2 Hz, 1H), 4.01 (s, 1H), 3.52 (q, *J* = 6.9 Hz, 2H), 2.34 (s, 5H), 2.23 (s, 5H), 1.25 (s, 6H), 1.06 (t, *J* = 6.9 Hz, 3H). ^13^C NMR (100 MHz, CDCl_3_) 156.58, 151.32, 150.20, 144.56, 143.90, 141.13, 135.45, 132.52, 132.34, 130.18, 127.48, 127.15, 124.74, 122.16, 120.50, 116.58, 77.24,70.64, 66.42, 64.06, 58.08, 56.35, 29.70, 27.68, 15.14, 14.59. HRMS-ESI calcd for C_32_H_38_BF_2_N_6_O_4_ [M + H] ^+^ 619.30102; found, 619.30182.

### 4.10 Synthesis of Compound **2c**

67 mg (0.086 mmol, 1 equiv) of **2b**, 27 mg (0.086 mmol, 1 equiv) of resiquimod, and 26 mg (0.128 mmol, 1.5 equiv) of 4-nitrophenyl chloroformate were transferred to a 2-neck reaction flask, and air was removed by repeated vacuum–nitrogen cycles. Afterward, 5 mL of dry THF was injected under nitrogen to dissolve the three compounds. At room temperature, 55.6 mg of DIPEA (0.43 mmol, 5 equiv) was injected dropwise. The mixture was stirred for 10 min at room temperature before refluxing at 35–40 °C for 24 h under nitrogen conditions. Afterward, the solvent evaporated, and the residue was then purified on a silica column using gradient elution: first, 100% ethyl acetate and finally ethyl acetate: methanol (20:1). The product was further purified using GPC to obtain 47 mg of a pure cyan solid in 54%. ^1^H NMR (400 MHz, DMSO-*d*_6_, δ): 10.19 (s, 1H), 8.54 (d, *J* = 8.3 Hz, 1H), 7.93 (d, *J* = 7.8 Hz, 1H), 7.66–7.60 (m, 1H), 7.55 (d, *J* = 6.6 Hz, 5H), 7.37 (s, 4H), 7.05 (d, *J* = 6.5 Hz, 4H), 7.01 (s, 2H), 5.49 (s, 2H), 4.92 (s, 2H), 4.73 (s, 3H), 4.15 (s, 4H), 3.77 (s, 4H), 3.59 (d, *J* = 2.6 Hz, 4H), 3.54 (dd, *J* = 8.8, 5.6 Hz, 12H), 3.47–3.41 (m, 6H), 3.38 (s, 6H), 1.27–1.03 (m, 12H). ^13^C NMR (100 MHz, CDCl_3_): δ 159.90, 153.20, 151.67, 150.08, 144.41, 143.90, 139.29, 136.80, 135.51, 134.03, 129.52, 129.21, 127.96, 127.61, 127.11, 124.86, 120.79, 118.39, 116.55, 116.30, 115.04, 77.26, 71.95, 70.89, 70.68, 70.59, 70.39, 69.69, 67.56, 66.27, 63.81, 59.05, 58.18, 56.39, 31.91, 29.68, 29.48, 29.34, 27.51, 22.67, 15.18, 14.49, 14.10. HRMS-ESI calcd for [M + H]^+^ C_60_H_75_BF_2_N_6_O_12_ 1119.54203; found, 1119.54504.

### 4.11 Synthesis of Compound **3c**

55 mg (0.071 mmol, 1 equiv) of **1e**, 27 mg (0.086 mmol, 1.2 equiv) of resiquimod, and 26 mg (0.128 mmol, 1.8 equiv) of 4-nitrophenyl chloroformate were transferred to a 2-neck round-bottom flask, and air was removed by repeated vacuum–nitrogen cycles. Afterward, 5 mL of dry THF was injected under nitrogen to dissolve the three compounds. At room temperature, 55.6 mg (0.43 mmol, 5 equiv) of DIPEA was injected dropwise. The mixture was stirred for 10 min at room temperature before refluxing at 35–40 °C for 24 h under nitrogen conditions. The solvent was evaporated, and the residue was then subjected to purification on a silica column using 100% ethyl acetate; the product was further purified using GPC to obtain about 50 mg of a pure blue solid (yield = 63% and 81%) based on **1e** because 12.5 mg was restored from column separation. ^1^H NMR (400 MHz, CDCl_3_, δ): 8.78 (s, 1H), 8.22 (d, *J* = 8.3 Hz, 1H), 8.11 (d, *J* = 8.1 Hz, 1H), 7.60 (t, *J* = 7.6 Hz, 1H), 7.52–7.42 (m, 7H), 7.06 (d, *J* = 16.2 Hz, 2H), 6.95 (d, *J* = 8.8 Hz, 4H), 6.69 (s, 2H), 5.59 (s, 2H), 4.81 (s, 2H), 4.73 (s, 2H), 4.22–4.11 (m, 4H), 3.94–3.84 (m, 4H), 3.79–3.72 (m, 4H), 3.72–3.64 (m, 8H), 3.62–3.57 (m, 2H), 3.57–3.54 (m, 4H), 3.38 (s, 6H), 1.29 (s, 5H), 1.25 (s, 8H), 1.18 (t, *J* = 7.0 Hz, 3H), 0.48 (s, 6H). ^13^C NMR (100 MHz, CDCl_3_): δ 173.29, 159.31, 159.31, 151.06, 150.74, 150.62, 144.54, 143.96, 136.82, 135.44, 133.40, 132.85, 130.57, 130.17, 128.42, 127.50, 127.30, 124.63, 119.84, 119.19, 118.81, 116.84, 115.08, 77.74,, 77.23,, 71.96, 71.40, 70.89, 70.69, 70.60, 69.71, 68.87, 67.55, 66.58, 64.83, 62.10, 59.43, 59.06, 56.46, 34.06, 31.92, 29.70, 29.66, 29.36, 29.27, 29.12, 27.84, 27.22, 24.86, 22.68, 16.33, 14.83, 14.11, 14.02. HRMS-ESI calcd for [M + H]^+^ C_62_H_80_BN_6_O_12_ 1111.59218; found, 1111.59497.

### 4.12 Chemical Actinometer for Quantum Yield Measurement^34^

The quantum yield (Φ) for the uncaging reaction was calculated using the formula Φ = (number of reacted molecules per time unit)/(number of photons absorbed per time unit). Ferrioxalate serves as a reliable chemical actinometer for measuring photon fluxes, decomposing upon irradiation to generate ferrous ions, which are quantified by their conversion to the colored tris–phenanthroline complex that absorbs at 510 nm (ε = 11,100 M^–1^ cm^–1^). The complexation of ferric ions with phenanthroline is negligible at this wavelength. The photon flux of a 365 nm LED lamp was determined through a series of steps: 0.3 g of K_3_[Fe(C_2_O_4_)_3_]·3H_2_O was dissolved in 50 mL of 0.05 M H_2_SO_4_ (solution 1), and 10.4 mg of 1,10-phenanthroline monohydrate and 2.25 g of CH_3_COONa·3H_2_O were dissolved in 10 mL of 0.5 M H_2_SO_4_ (solution 2). A 3.3 mL aliquot of solution 1 was irradiated at 365 nm for 0, 0.2, 0.4, and 0.8 s. After each irradiation, 0.5 mL of solution 2 was added, and the absorption spectra were measured (Figure S1A). Changes in absorbance at 510 nm relative to the irradiation time were used to calculate the photon flux from the following equation
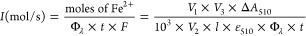
*V*_1_: irradiated volume (mL), *V*_2_: aliquot of irradiated solution taken for determining ferrous ions (mL), *V*_3_: final volume (mL), Δ*A*_510_: absorbance difference between solutions before and after irradiation, *I*: optical path length of the irradiation cell, ε_510_: molar extinction coefficient of Fe(phen)32+ at 510 nm, Φ_363.8_: quantum yield of ferrous ion generation at the irradiation wavelength (1.28),^34^*t*: irradiation time, *F*: mean function of light absorbed by the ferrioxalate solution. The photon flux of the 365 nm LED lamp was measured to be 2.84 × 10^–7^ mol/min (Figure S1B).

### 4.13 Thermal Stability Studies

The thermal stability of compounds **1–3c** was systematically investigated in DMSO-*d*_6_ by using ^1^H NMR spectroscopy. The compounds were incubated at 37.5 °C in NMR tubes and protected from light to prevent photodegradation. The DMSO-*d*_6_ signal served as an internal standard, consistently observed within the chemical shift range of 2.52 and 2.47 ppm across all spectra. To ensure reproducibility and accuracy, the degradation process was monitored by analyzing the secondary amine (NH) signal, the characteristic doublet of resiquimod, and a singlet at around 5 ppm (Figures S3–S5). The mean degradation of these three signals was calculated to provide a quantitative assessment of the thermal stability of the compounds.

### 4.14 Safety Evaluation (MTT Assay)

The potential cytotoxicity of compounds in AD293 cells was evaluated by the tetrazolium salt 3-[4,5-dimethylthiazol-2-yl]-2,5-diphenyltetrazolium bromide (MTT) assay. AD293 cells were seeded in a 96-well plate (4 × 10^4^ cells/well) in Dulbecco’s modified Eagle’s medium (DMEM) with 10% fetal bovine serum and penicillin/streptomycin (100 U/ml and 100 μg/mL, respectively). One day after cell seeding, the cells were treated with the vehicle (1% phosphate buffered saline) or each compound in serum-free DMEM for 24 h. Ten μL of MTT reagent (5 mg/mL) was added to all wells, and plates were incubated for 3 h at 37 °C. After incubation, the culture medium was removed, and 100 μL of DMSO was added to each well. The absorbance of each well at 590 nm was measured by using a microplate reader. All assays were performed in triplicate, and the results were obtained in four independent experiments.

### 4.15 Establishment of a Stable AD293 Cell Line with Constitutive TLR7 Expression

AD293 cells were seeded at a density of 0.40 million cells per 35 mm dish. 24 h later, cells were transfected with a TLR7 plasmid (cat: P190715-01-B08-E10, Vector Builder) using GenJet In Vitro DNA Transfection Reagent (Ver. II) (cat: SL100489, SignaGen). Hygromycin B selection (250 μg/mL) (cat: 084-07681, FUJIFILM Wako) was initiated 24 h post-transfection and repeated every other day for a total of 10 days. Stable TLR7-expressing AD293 cell lines were established 14 days post-transfection.

### 4.16 *N*-Acetyl-cysteine Treatment

AD293 cells stably expressing TLR7 were seeded in 96-well plates at 20,000 cells per well. After 24 h, the culture medium was replaced with Opti-MEM (Cat: 11058021, Thermo Fisher Scientific). One hour later, cells were treated with 10 mM *N*-acetyl-cysteine (Merck, cat: A8199). Thirty minutes post-treatment, cells were further stimulated with 10 μM **3c** or **1e**. Subsequently, cells were exposed to 650 nm LED light (DREAM CAST LIGHT, Kousho Co., Ltd.) for 30 min. The control group was shielded from LED light and underwent the same experimental procedures. 24 h postdrug treatment, cells were observed using a microscope (IX71, OLYMPUS).

### 4.17 Spatiotemporal Regulation of TLR7 Receptors (Western Blotting)

Western blotting was conducted as described previously.^47^ The cell lysates were separated by 10% SDS-polyacrylamide gel electrophoresis and blotted onto PVDF membranes. Nonspecific binding was decreased with a blocking buffer (5% skim milk or 5% bovine serum albumin), and the membranes were subsequently incubated with primary antibodies overnight at 4 °C. The following antibodies were used: tIκB (1:1000, cat: 9242, RRID: AB_331623, Cell Signaling Technology), and GAPDH (1:100,000; cat: 014–25,524, RRID: AB_2814991, FUJIFILM Wako). Following washing, the membranes were incubated with a horseradish peroxidase (HRP)-conjugated secondary antibody (1:5,000, goat antimouse IgG-HRP, catalog no. 115-035-003, RRID: AB_10015289; 1:5,000, goat antirabbit IgG-HRP, catalog no. 111-035-003, RRID: AB_2313567; Jackson ImmunoResearch Laboratories) for 1 h at room temperature. Chemiluminescence was detected using a Clarity Western ECL Substrate.

### 4.18 Analysis of M1Macrophage Marker Gene Expression (Quantitative PCR)

A quantitative polymerase chain reaction (PCR) was conducted as described previously.^47^ Mouse macrophage cell line RAW264.7 cells were maintained in DMEM supplemented with 10% fetal calf serum and penicillin/streptomycin (100 units/ml and 100 μg/mL, respectively) in an incubator in 5% CO_2_ at 37 °C. RAW264.7 cells were seeded at a density of 0.3 million cells per 35 mm dish. One day after cell seeding, cells were treated with 10 mM *N*-acetyl-cysteine (cat: A8199, Merck) to prevent the influence of reactive oxygen. Thirty minutes post-treatment, cells were further stimulated with 100 nM **3c** or **1e**. Subsequently, cells were exposed to 650 nm LED light (DREAM CAST LIGHT, Kousho Co., Ltd.) for 30 min. 24 h later, total RNA was extracted using a previously described method and used to synthesize cDNA with M-MLV reverse transcriptase (Thermo Scientific, Waltham, MA, USA; Cat: 28025013) and random primers (Thermo Scientific; cat: 48190011). The cDNAs synthesized using 1 μg of total RNA in each sample were subjected to real-time PCR assays with specific primers and THUNDERBIRD Next SYBRTM qPCR Mix (cat: QPX-201; Toyobo, Osaka). The sequences of primers are as follows: inducible nitric oxide synthase (iNOS), 5′-CAAGCACCTTGGAAGAGGAG-3′(forward) and 5′-AAGGCCAAACACAGCATACC-3′ (reverse); tumor necrosis factor-alpha (TNF-α), 5′-AACCACCAAGTGGAGGAG-3′ (forward) and 5′-CAGCCTTGTCCCTTGAAG-3′ (reverse); glyceraldehyde-3-phosphate dehydrogenase (GAPDH), 5′-AGCCCAGAACATCATCCCTG-3′ (forward) and 5′-CACCACCTTCTTGATGTCATC-3′ (reverse). Real-time PCR assays were conducted using a DNA engine Opticon 2 real-time PCR detection system (Bio-Rad Laboratories, Inc., Hercules, CA, USA). The three-step amplification protocol consisted of 3 min at 95 °C followed by 40 cycles at 95 °C for 15 s, 60 °C for 30 s, and 72 °C for 30 s. RNA quantities of target genes were calculated by using the *C*_t_ method. The *C*_t_ values of each amplification were normalized to that of GAPDH amplification.

### 4.19 Singlet Oxygen Quantum Yield (Φ_Δ_)

Φ_Δ_ was determined in acetonitrile by monitoring the photooxidation of 1,3-diphenylisobenzofuran (DPBF), which absorbs at ∼410 nm and forms a colorless product upon reaction with ^1^O_2_. As DPBF scavenges singlet oxygen, a gradual decrease in absorbance indicates ^1^O_2_ generation. To minimize the quenching of ^1^O_2_ by the tested compounds, the singlet oxygen quantum yields were measured at a 5–10-fold higher concentration of DPBF (20–25 μm) relative to the concentration of the investigated compounds (2.5–5 μm). Irradiation was carried out using monochromatic light at either 660 or 525 nm (3–5 mW), and the absorbance of DPBF at 410 nm was recorded at various time intervals (Figure 8). Methylene blue was used as a reference for the Φ_Δ_ measurements of **2–3c**, **2b**, and **1e**. While Rose bengal served as the reference for **1c** and **1b**. The quantum yields of the tested compounds were calculated by the relative method using the following equation.^48^
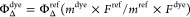
where Φ_Δ_^ref^ is the Φ_Δ_ of methylene blue (0.52)^49^ or rose bengal (0.76),^50^ m is the slope of the photobleaching rate of DPBBF at 410 nm (Figure S11), and *F* is the absorption correction factor given by (*F* = 1–10^–OD^) (optical density at irradiation wavelength).

### 4.20 Cell Culturing

A549, HaCaT, and HeLa cells were grown in Dulbecco’s modified Eagle’s medium (DMEM) with 10% fetal bovine serum, 2 mM l-glutamine, and penicillin/streptomycin (100 U/ml and 100 μg/mL, respectively) under standard growth conditions (37 °C, 5% CO_2_) in humidified air in an incubator. At about 70–90% confluency, cells were detached from the flask by accutase enzyme treatment, pelleted, and subcultured at a concentration of 5 × 10^5^ to 1 × 10^6^ cells in 20 mL complete cell culture medium per T75 flask. Cells were subcultured every three to 4 days (twice a week). Before starting any experiment, cells were passaged at least three times after thawing, taking into consideration not to exceed 20 passages (after thawing).

### 4.21 Photodynamic Effect (CCK-8 Assay)

The photodynamic effect (phototoxicity) of resiquimod and caged-resiquimods (**1c**, **3c**) was assessed using the CCK-8 assay under light and dark conditions. A549, HaCaT, and HeLa cells were seeded (2 × 10^3^ cells/well) in phenol red free-DMEM supplemented with 10% FBS, l-glutamine, and penicillin/streptomycin. After 24 h, the medium was replaced with a new one containing 1% DMSO and 10 μM of each compound. Two 96-well plates per cell line were prepared: one kept in the dark and another exposed to 530 or 650 nm LED light (30 mW) for 15 min after a 90 min incubation. Plates were then incubated for 72 h, media were replaced, and the CCK-8 reagent was added. Absorbance at 450 nm was measured before and after a 1–4 h incubation. Assays were conducted in triplicate (HaCaT in six replicates), and the cell viability was calculated relative to the dark control.

### 4.22 3D Spheroid Experiment

HeLa and A549 cells were cultured in Earle’s Minimum Essential Medium (E-MEM) supplemented with 10% heat-inactivated fetal bovine serum (HI-FBS) and 1% nonessential amino acids (NEAA) at 37 °C in a humidified incubator with 5% CO_2_. For 3D spheroid formation, 10,000 cells per well were seeded into ultralow attachment 96-well round-bottom plates. The cells were incubated for 3 days for A549 cells and 7 days for HeLa cells to form spheroids before treatment with compounds at the desired concentrations. After a 2 h incubation with the compounds, one plate of each cell line was either irradiated with 650 nm red light for 30 min or kept as nonirradiated control. The plates were then returned to the incubator, and spheroids were cultured for 72 h in a dark environment. After treatment with compounds, the media were removed from spheroids, and cell viability and cytotoxicity were assessed using the Viability/Cytotoxicity Kit for mammalian cells. Following the manufacturer’s protocol, spheroids were stained with Calcein-AM and Ethidium Homodimer-1 to differentiate live (green fluorescence) and dead (red fluorescence) cells. Staining was carried out for 1 h at 37 °C in the dark, followed by imaging with a fluorescent microscope (Axio Observer D1, Zeiss, Oberkochen, Germany). Fluorescence intensity values were analyzed to quantify the proportions of live and dead cells, normalized to 0.1% DMSO treated controls, to evaluate the effects of the compounds under irradiated and nonirradiated conditions.

## Supporting Information Available

Molecular docking, photophysical and photochemical properties, photoreactions of **1–3c**, safety assays, singlet oxygen quantum yield, HPLC purity analysis, NMR spectra, ESI-MS data, and results (PDF)
Molecular formula strings (CSV)
