# Supplementary material for: Design, Synthesis, and Biological Evaluation of BODIPY-Caged Resiquimod as a Dual-Acting Phototherapeutic
Source: J Med Chem. 2025 Feb 17;68(4):4561–81. doi: 10.1021/acs.jmedchem.4c02606 (PMC11873906; doi:10.1021/acs.jmedchem.4c02606)
Supplement: Supplementary file 1 — jm4c02606_si_001.pdf [file jm4c02606_si_001.pdf]

Design, Synthesis and Biological Evaluation of BODIPY-caged Resiquimod as a Dual-Acting Phototherapeutic

Eslam Roshdy[1,2], Haruto Taniguchi[3], Yoki Nakamura[3], Haruko Takahashi[4], Yutaka Kikuchi[4], Ismail Celik[5], Elsayed S. I. Mohammed [6,7], Yasuhiro Ishihara[8], Norimitsu Morioka[3], Manabu Abe*[1]

[1] Department of Chemistry, Graduate School of Advanced Science and Engineering, Hiroshima University, Higashi-Hiroshima City, Hiroshima 739-8526, Japan.

[2] Medicinal Chemistry Department, Faculty of Pharmacy, Minia University, Minia, 61519, Egypt.

[3] Department of Pharmacology, Graduate School of Biomedical & Health Sciences, Hiroshima University, Kasumi 1-2-3, Minami-ku, Hiroshima 734-8553, Japan.

[4]Graduate School of Integrated Sciences for Life, Hiroshima University, 1-3-1 Kagamiyama, Higashi-Hiroshima 739-8526, Hiroshima, Japan.

[5] Department of Pharmaceutical Chemistry, Faculty of Pharmacy, Erciyes University, 38039 Kayseri, Turkey.

[6]Avian Research Center, King Faisal University, Al Hofuf, Al-Ahsa 31982, Saudi Arabia.

[7] Department of Histology, Faculty of Veterinary Medicine, South Valley University, Qena 83523, Egypt.

[8] Program of Biomedical Science, Graduate School of Integrated Sciences for Life, Hiroshima University, 1-7-1, Kagamiyama, Higashi-Hiroshima, Hiroshima 739-8521, Japan

* Corresponding author, E-mail: mabe@hiroshima-u.ac.jp

Contents

I. **Molecular Docking Results.** 3
II. **Photophysical and Photochemical Properties.** 3
III. **Photoreaction of compound 1c.** 6
IV. **Photoreaction of Compound 2c.** 7
V. **Photoreaction of compound 3c.** 8
VI. **Safety Assay of 3c, 1e and 2e on AD293 .** 10
VII. **Singlet Oxygen Quantum yield Studies.** 11
VIII. **HPLC Purities of 1-3 Compounds.** 15
IX. **NMR Characterization of all synthesized compounds** 17

I. Molecular Docking Results.

NH$_2$-Caging		OH-Caging	
Ligand	Docking Score	Ligand	Docking Score
Resiquimod	-9.506		
Ra	-	Ra`	-8.888
Rb	-	Rb`	-
Rc	-	Rc`	-
Rd	-4.948	Rd`	-9.426
Re	-	Re`	-9.531
Rf	-6.110	Rf`	-7.149
Rg	-5.899	Rg`	-9.618
Rh	-	Rh`	-

Table S1. Summary of the docking scores, indicative of binding energies (kcal mol^{-1}), for Amino-Caged Resiquimods (Ra-h) and Hydroxy-Caged Resiquimods (Ra`-h`) in association with TLR7 (PDB ID: 5GMH).

II. Photophysical and Photochemical Properties.

A. Chemical actinometer for quantum yield measurement.

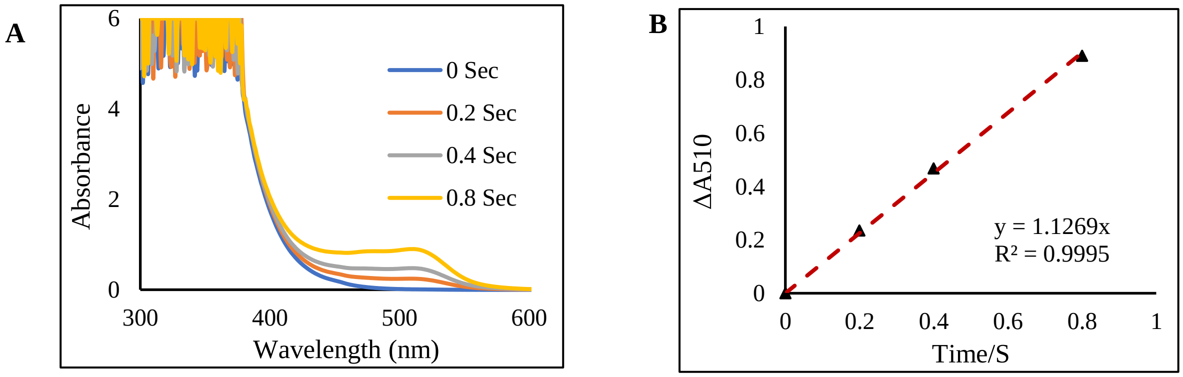

Figure S1. A: Time profile of photoreaction of $Fe(C_2O_4)_3^{3+}$ after irradiation at 365 nm. **B**; Plot of absorbance change at 510 nm of $Fe(C_2O_4)_3^{3+}$ after irradiation at 365 nm.

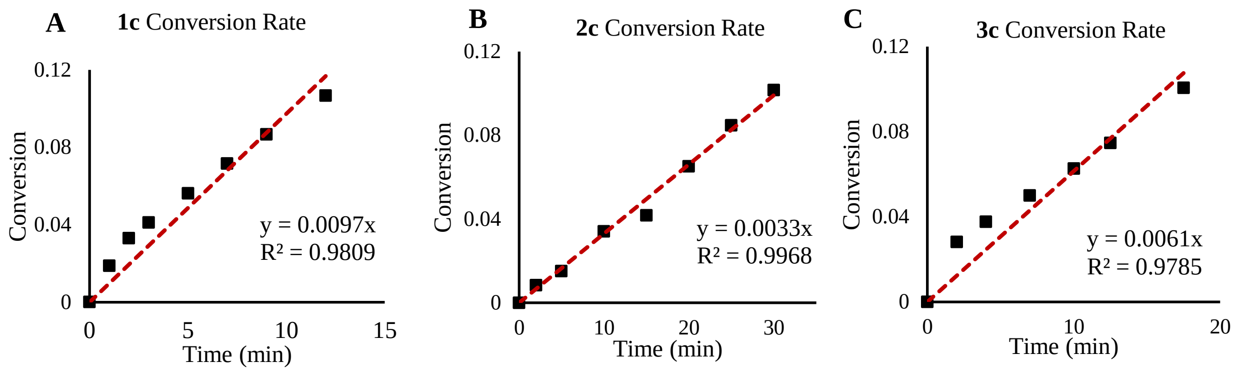

Figure S2. **A**, **B** and **C** show the rate of 10% conversion of **1c**, **2c** and **3c**, respectively, when irradiated with a 365nm LED lamp (Awill lamp).

B. Thermal Stability of 1-3 compounds at 37.5

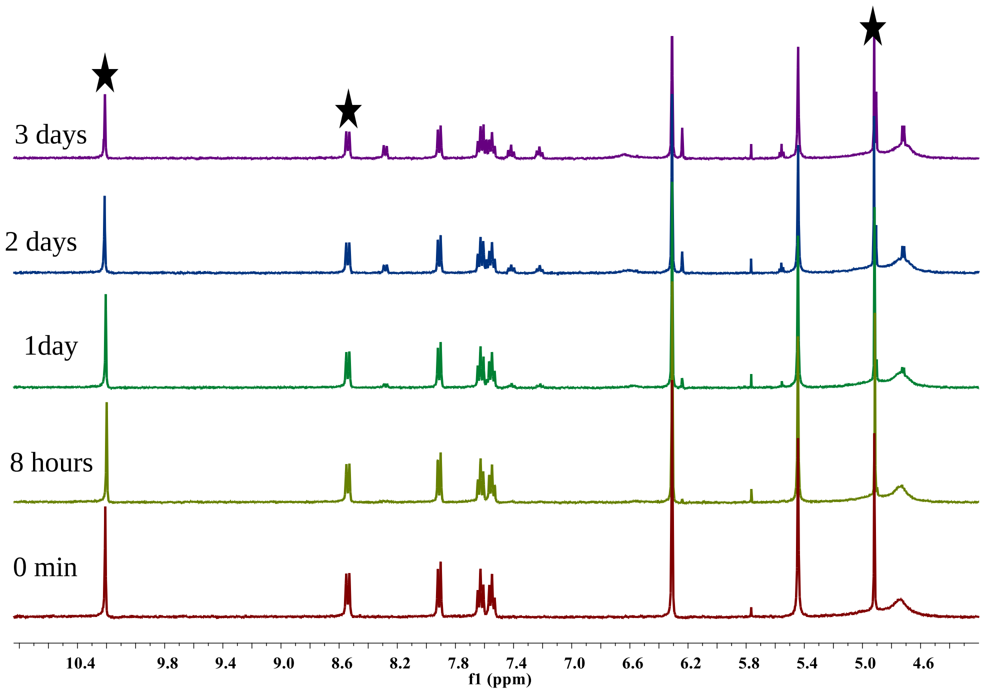

Figure S3. Thermal Stability of compound 1c. ^{1}H NMR spectra for 1c measured periodically while incubation in dark at 37.5 °C in DMSO-d_6. The decomposition was calculated based on three different signals, one signal from the resiquimod part, one from the BODIPY part, and the NH signal as a linkage part (assigned by black stars).

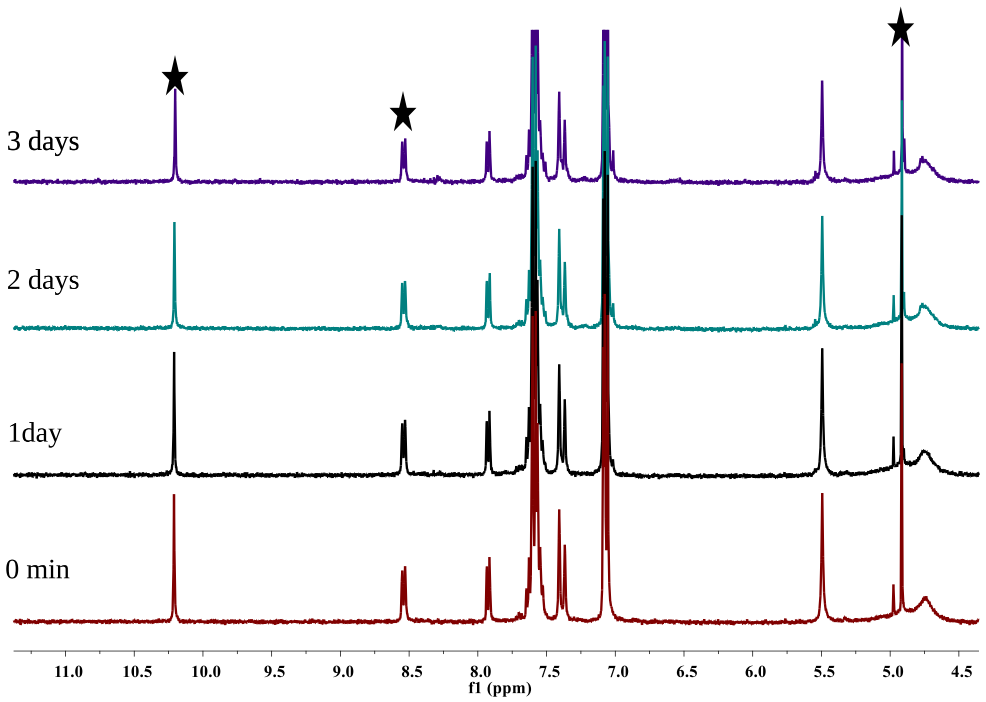

Figure S4. Thermal Stability of compound 2c. ^{1}H NMR spectra for 1c measured periodically while incubation in dark at 37.5 °C in DMSO-d_6. The decomposition was calculated based on three different signals, one signal from the resiquimod part, one from the BODIPY part, and the NH signal as a linkage part (assigned by black stars)

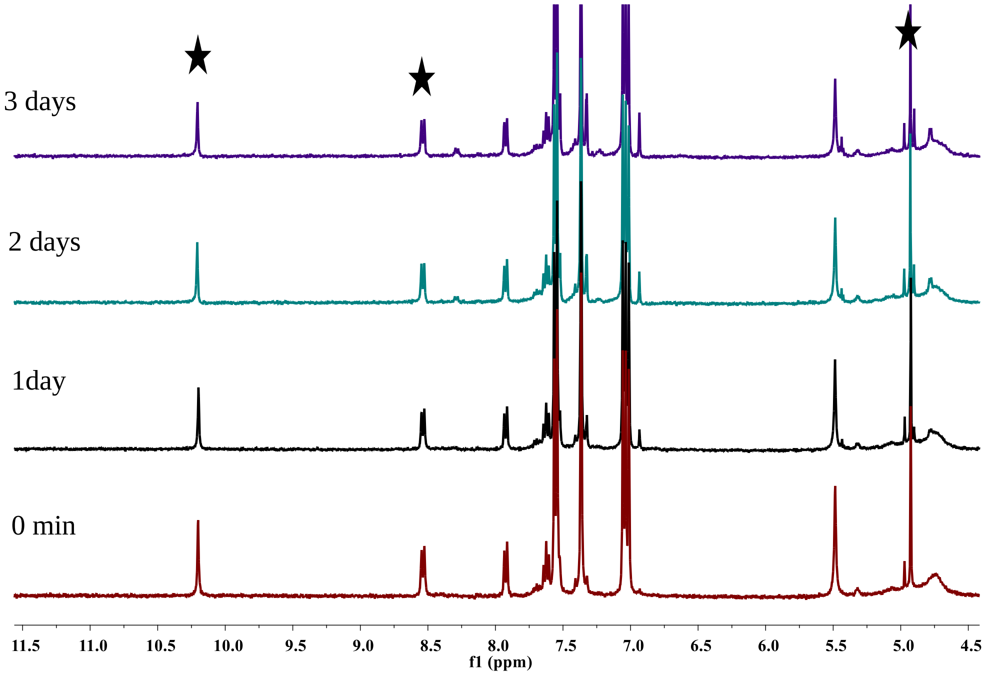

Figure S5. Thermal Stability of compound 3c. ^{1}H NMR spectra for 1c measured periodically while incubation in dark at 37.5 °C in DMSO-d_6. The decomposition was calculated based on three different signals, one signal from the resiquimod part, one from the BODIPY part, and the NH signal as a linkage part (assigned by black stars).

III. Photoreaction of compound 1c.

A. HERMS-ESI of 1c photolysate

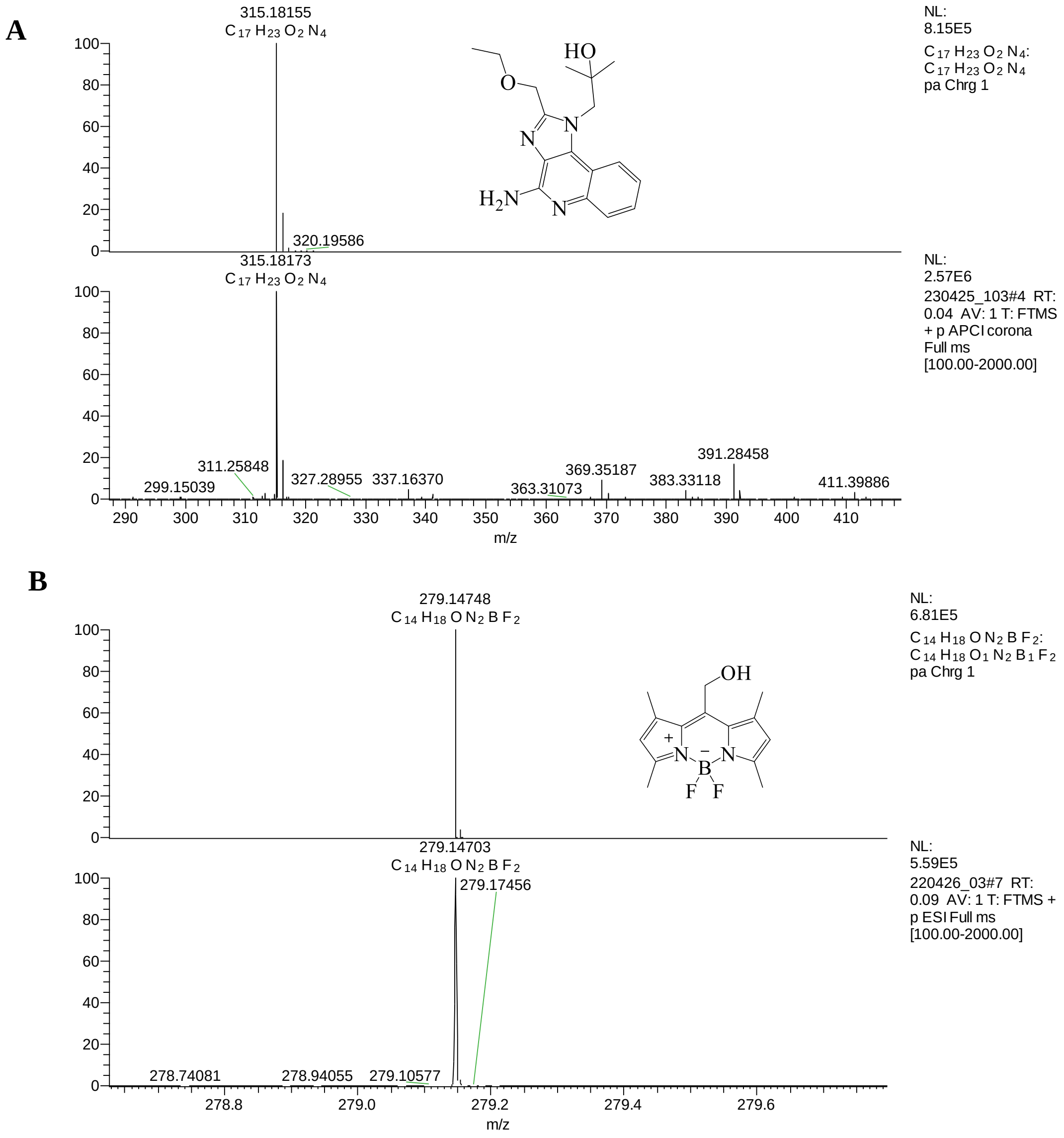

Figure S6. HERMS-ESI measurements of 1c photolysate. A: Shows resiquimod HRMS-ESI calcd for $C_{17}H_{23}N_4O_2$ $[M+H]^+$ 315.18155, found 315.18173.; **B** shows **1b** HRMS-ESI calcd for $C_{14}H_{18}BF_2N_2O$ $[M+H]^+$ 279.14748, found 279.14703.

B. Resiquimod calibration curve

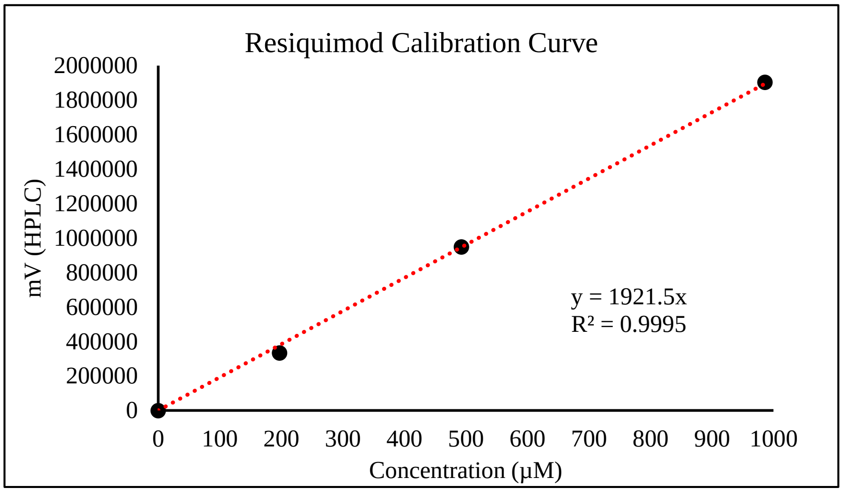

Figure S7. Resiquimod Calibration Curve.

IV. Photoreaction of Compound 2c.

A

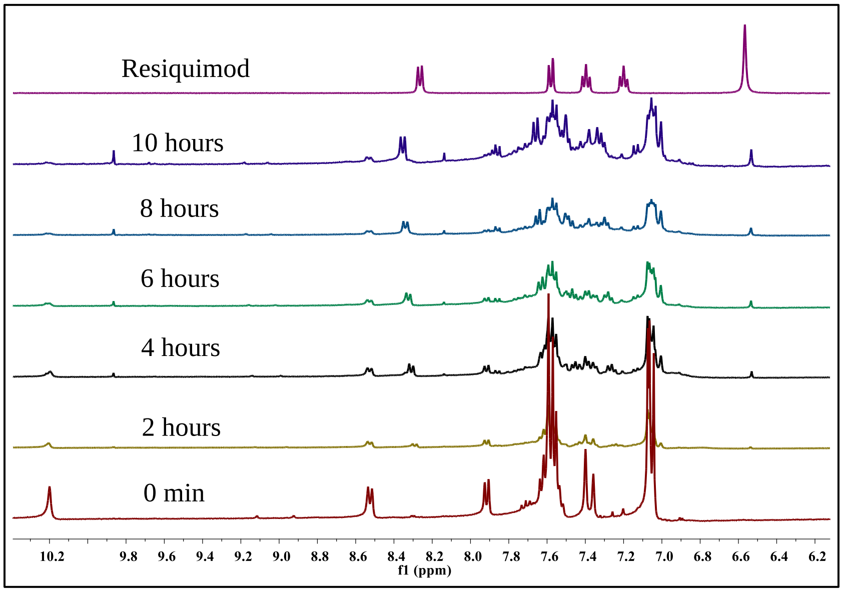

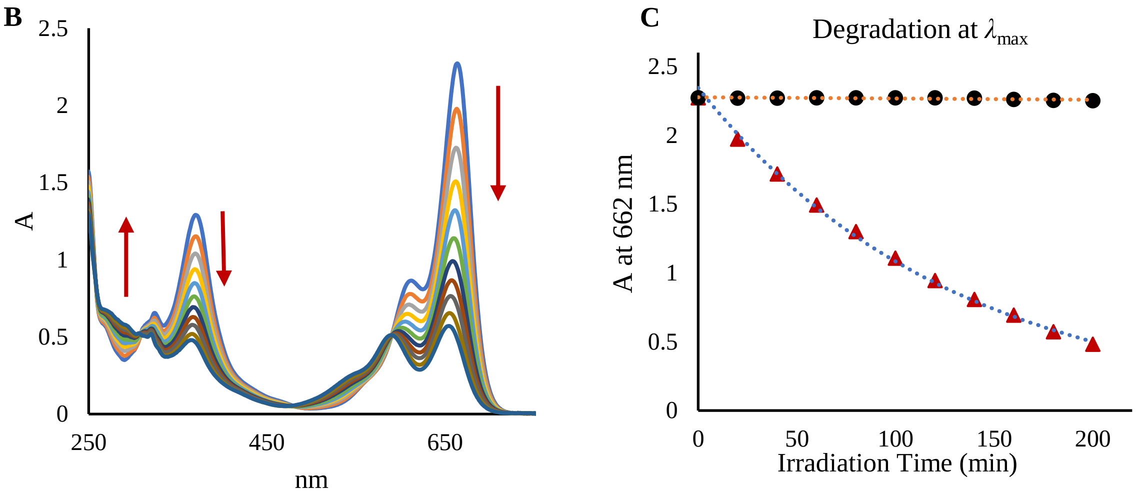

Figure S8. A; ^{1}H NMR spectra (400 MHz, 6.2–10.2 ppm) acquired during the photolysis of compound **2c** using a 660 nm LED lamp in DMSO-d_6. The spectra are presented sequentially, illustrating compound **2c** prior to irradiation, after 2, 4, 6, 8 and 10 of irradiation combined with ^{1}H NMR spectrum of resiquimod in DMSO-d^6. **B:** Photolysis of compound **2c** in a solvent mixture of 50% acetonitrile and 50% water was monitored at 20-minute intervals. **C;** Temporal decline in the **2c** absorption maximum at 662 nm.

V. Photoreaction of compound 3c.

A

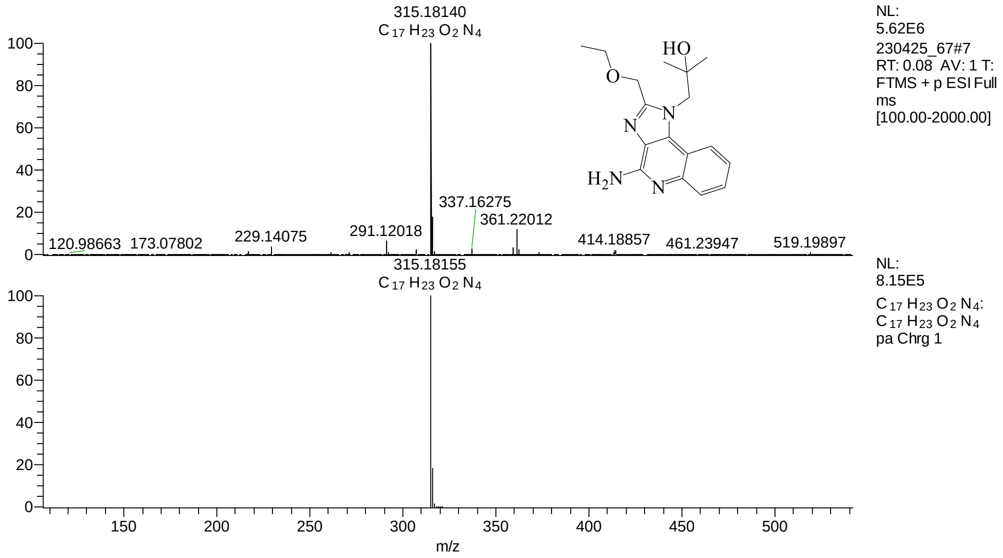

B

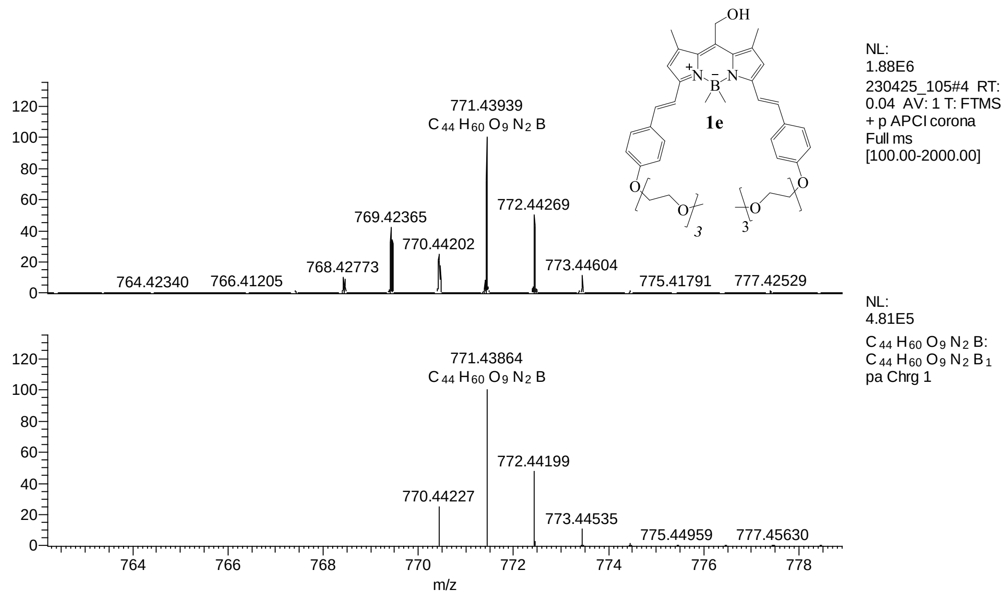

C

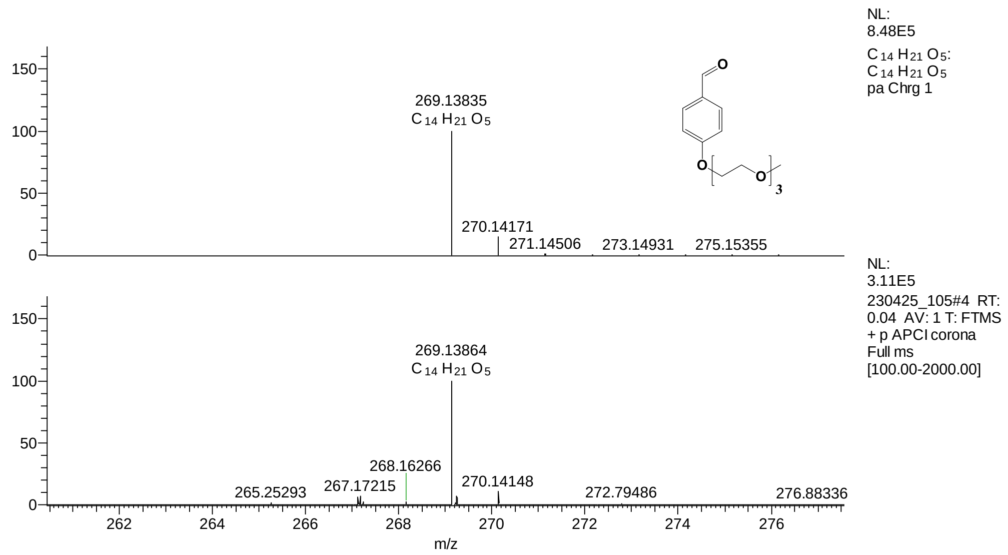

Figure S9. HERMS-ESI measurements of 3c photolysate. A: Shows resiquimod HRMS-ESI calcd for $C_{17}H_{23}N_4O_2$ $[M+H]^+$ 315.18155, found 315.18140. **B;** shows **1e** HRMS-ESI calcd for $C_{44}H_{60}O_9N_2B$ $[M+H]^+$ 771.43939, found 771.43864. **C;** shows **2e** HRMS-ESI calcd for $C_{14}H_{21}O_5$ $[M+H]^+$ 268.13835, found 268.13864.

VI. Safety Assay of 3c, 1e, and 2e on AD293.

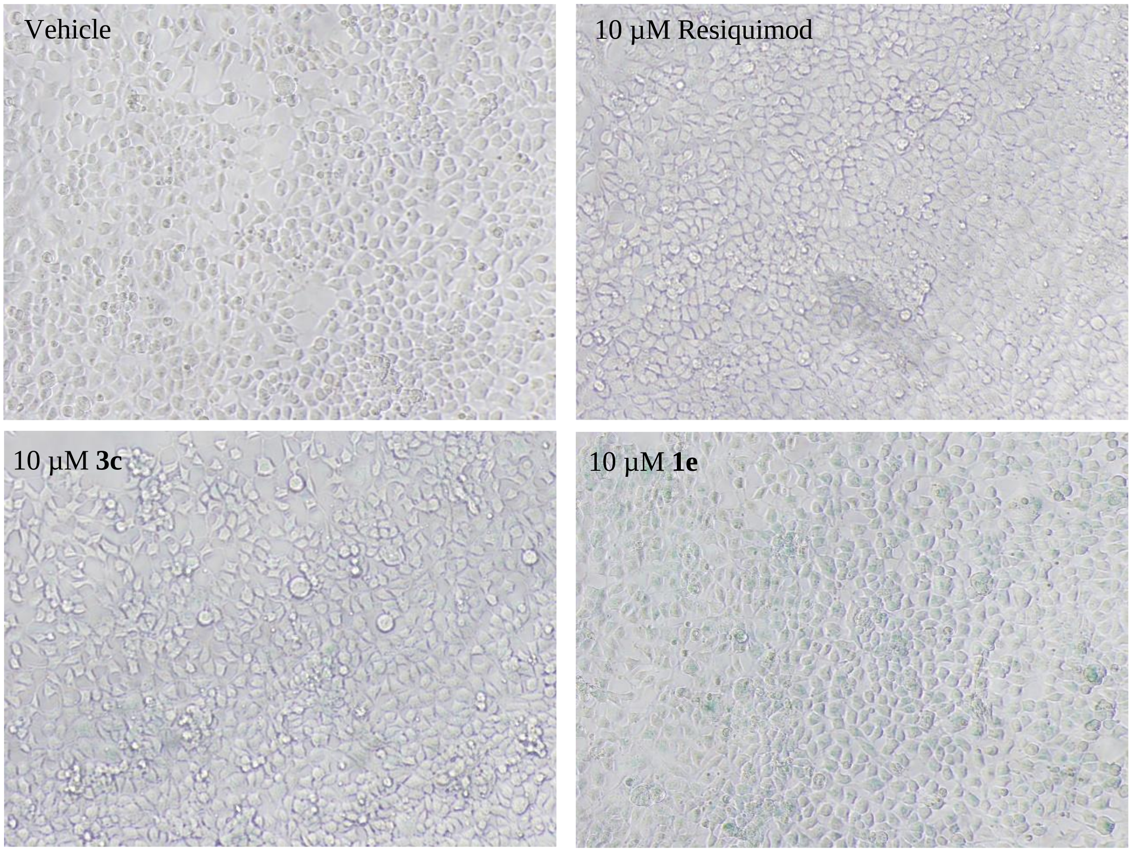

Figure S10. Evaluation of the effects of compounds **3c** and **1e** on AD293 cell morphology withought red light irradiation

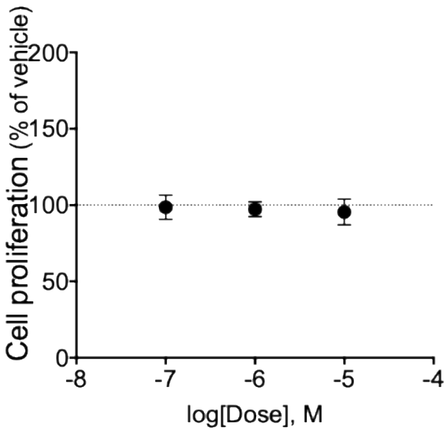

Figure S11. Impact of compound **2e** on AD293 cell viability.

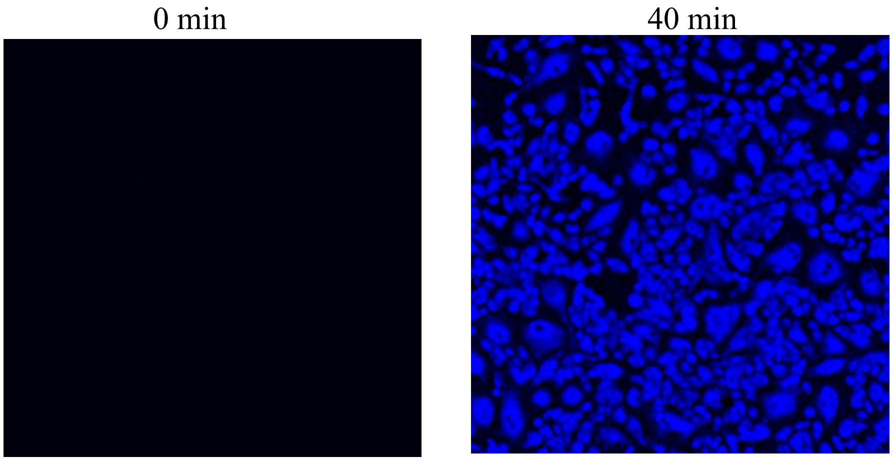

Figure S12: Time-Dependent Intracellular Fluorescence of Compound 1e in A549 Cells.

VII. Singlet Oxygen Quantum yield Studies.

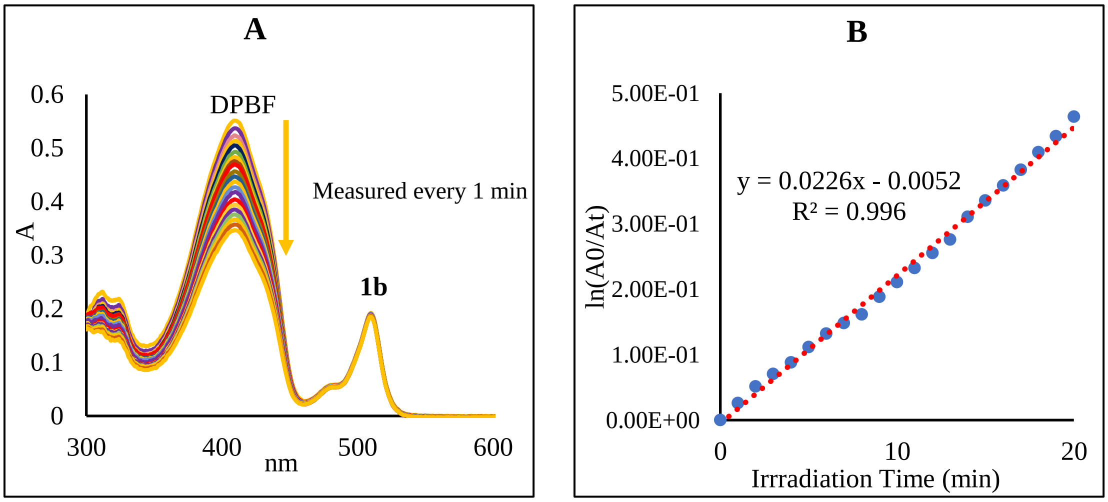

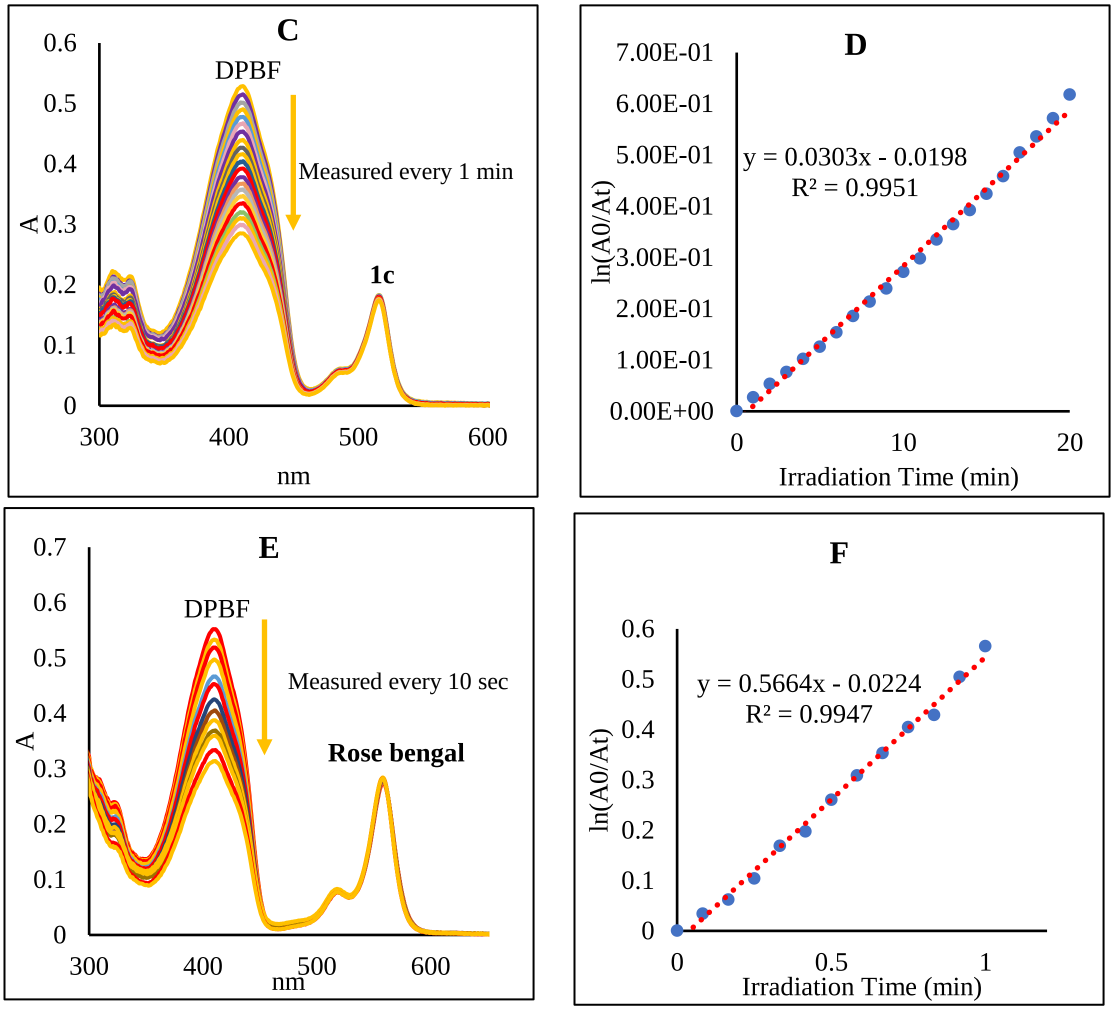

Figure S13. **A**, **C**, and **E** illustrate the changes in the absorption spectra of DPBF upon irradiation in the presence of compound **1b**, compound **1c**, and rose bengal, respectively, at various time intervals. Correspondingly, panels **B**, **D**, and **F** depict the variation in absorbance values (A_0 and A_t) over the course of irradiation for compound **1b**, compound **1c**, and rose bengal in acetonitrile. Here, A_0 represents the absorbance at the initial irradiation time, and A_t denotes the absorbance at a given irradiation time, both measured at 410 nm.

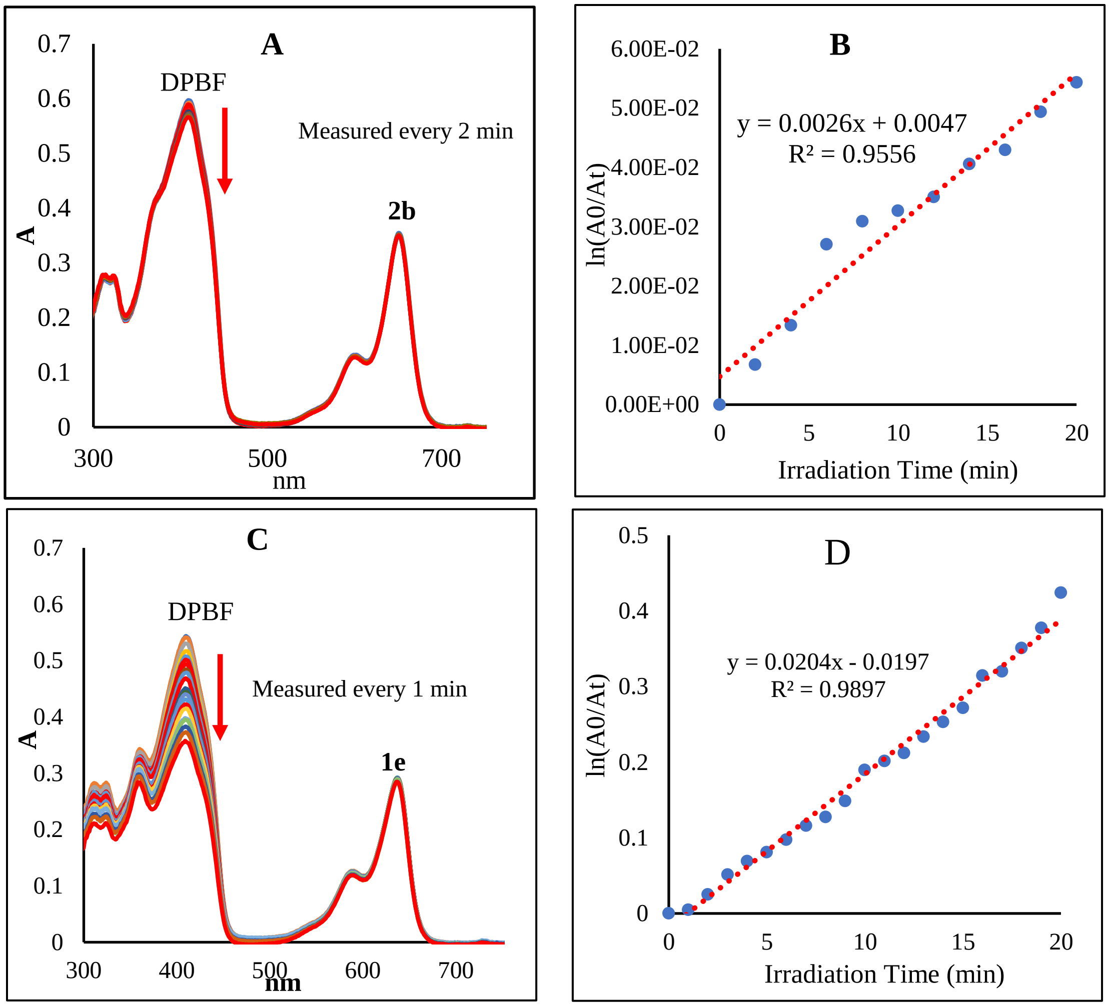
A
DPBF
Measured every 2 min
2b
A
nm
0.7
0.6
0.5
0.4
0.3
0.2
0.1
0
300
500
700
B
y = 0.0026x + 0.0047
R² = 0.9556
ln(A0/At)
Irradiation Time (min)
6.00E-02
5.00E-02
4.00E-02
3.00E-02
2.00E-02
1.00E-02
0.00E+00
0
5
10
15
20
C
DPBF
Measured every 1 min
1e
A
nm
300
400
500
600
700
D
y = 0.0204x - 0.0197
R² = 0.9897
ln(A0/At)
Irradiation Time (min)
0.5
0.4
0.3
0.2
0.1
0

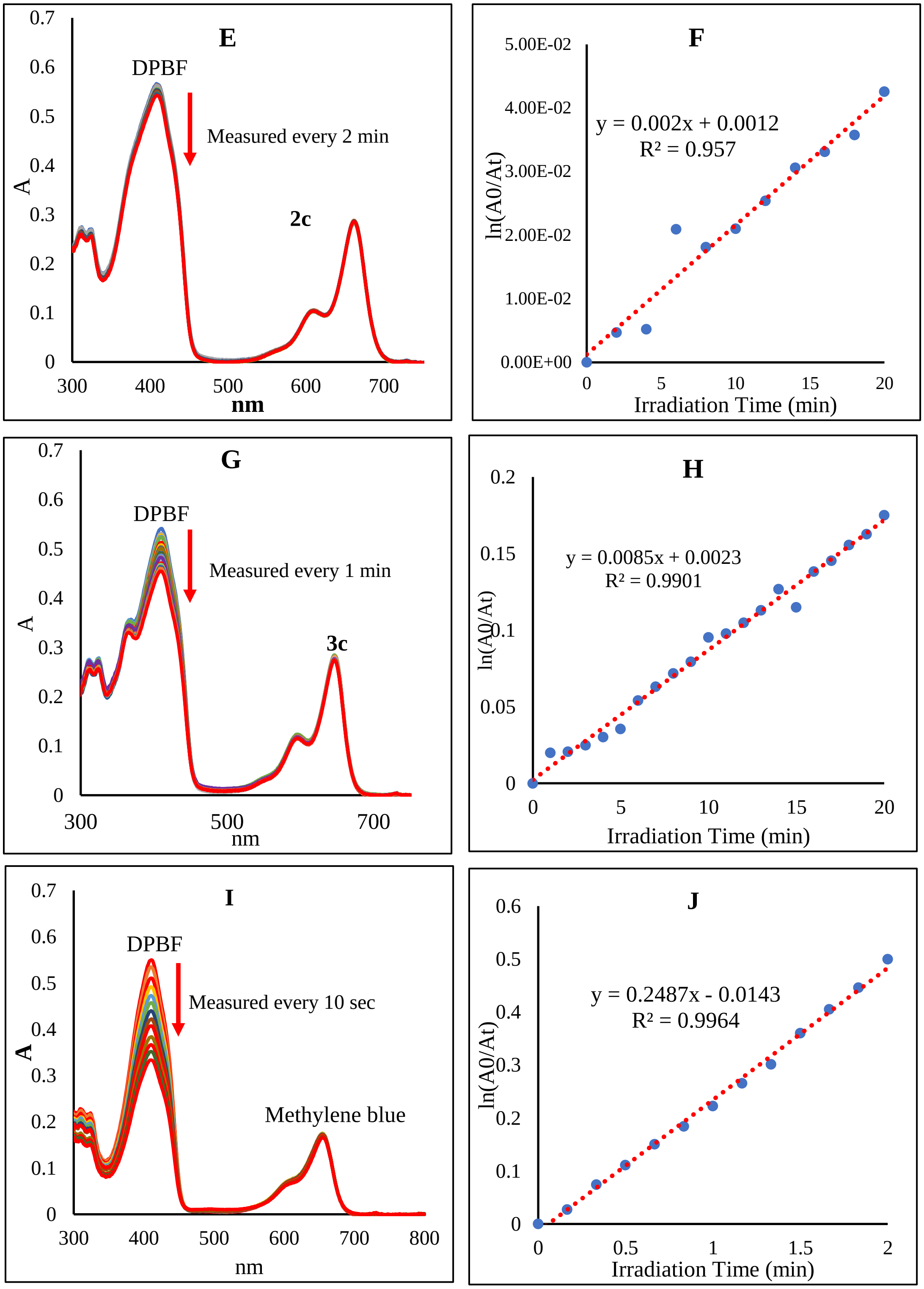

Figure S14. A, C, E, G and I illustrate the changes in the absorption spectra of DPBF upon irradiation in the presence of compounds **2b**, **1e**, **2c**, **3c** and methylene blue, respectively, at various time intervals. Correspondingly, panels B, D, F, H and J depict the variation in absorbance values (A_0 and A_t) over the course

of irradiation for compounds **2b**, **1e**, **2c**, **3c** and methylene blue in acetonitrile. Here, A_0 represents the absorbance at the initial irradiation time, and A_t denotes the absorbance at a given irradiation time, both measured at 410 nm.

VIII. HPLC Purities of 1-3 Compounds.

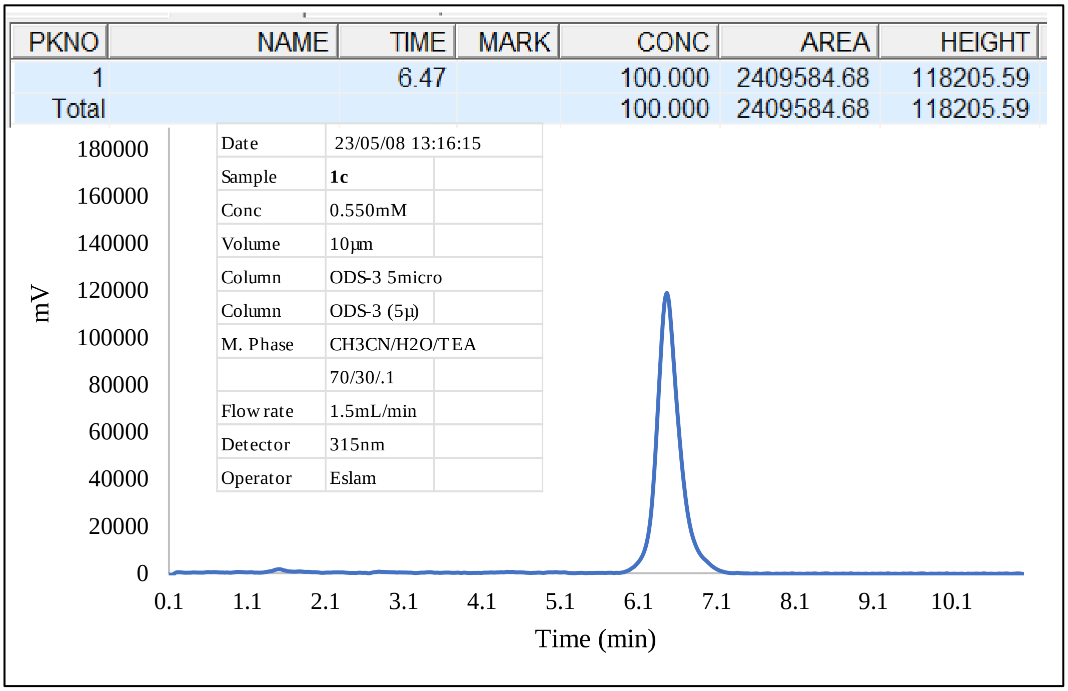

Figure S15. HPLC Spectrum of compound **1c**

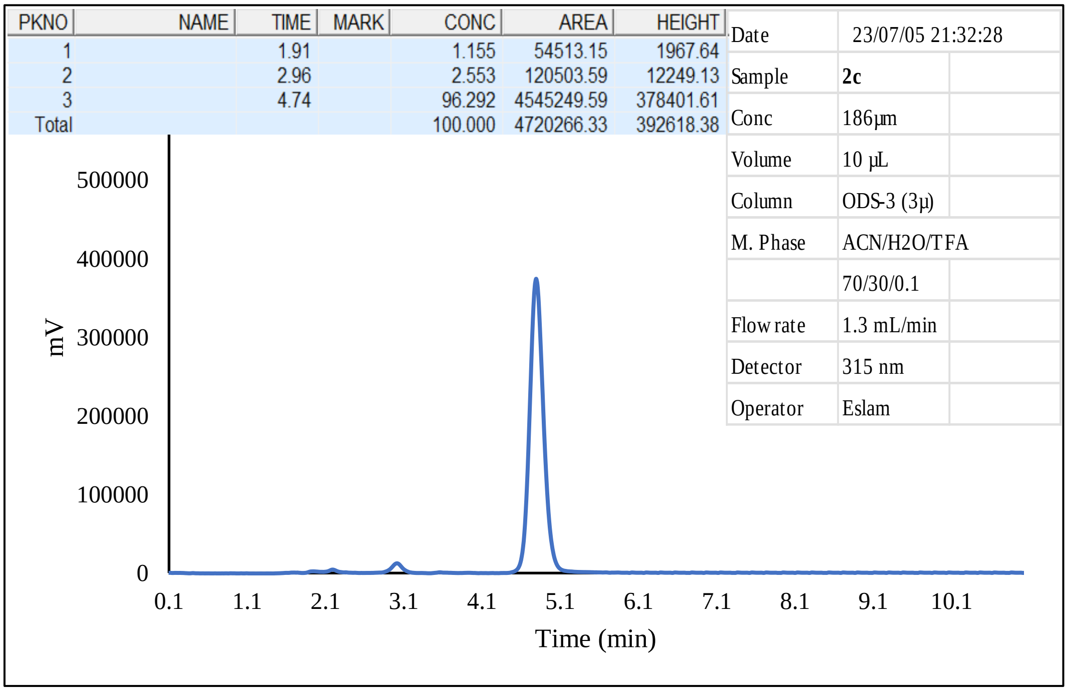

Figure S16. HPLC Spectrum of compound **2c**

PKNO	NAME	TIME	MARK	CONC	AREA	HEIGHT
1		1.88		0.276	7409.30	1081.39
2		7.78		99.724	2678753.66	172211.95
Total				100.000	2686162.96	173293.34

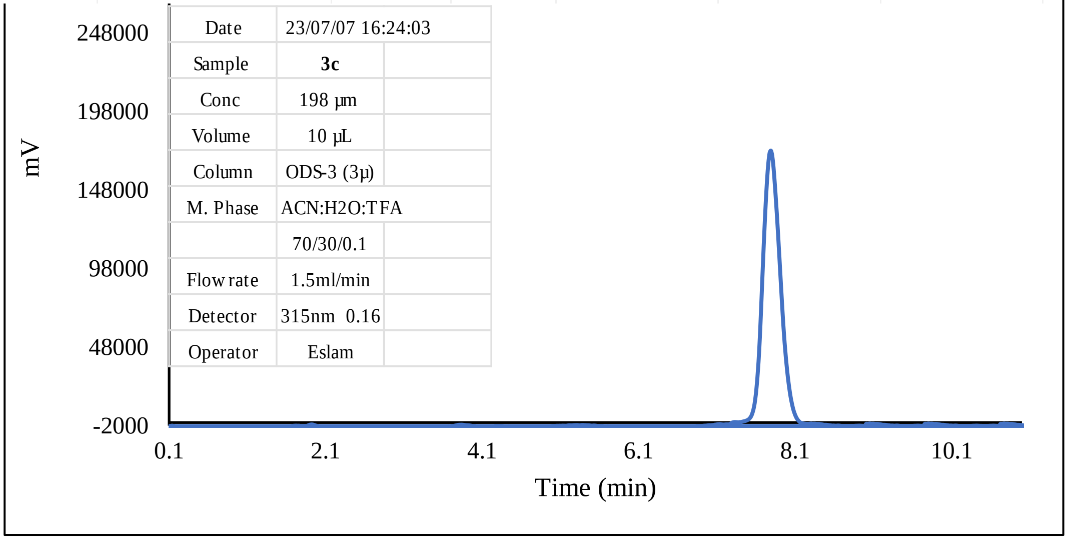

Figure S17. HPLC Spectrum of compound **2**

IX. NMR Characterization of all synthesized compounds

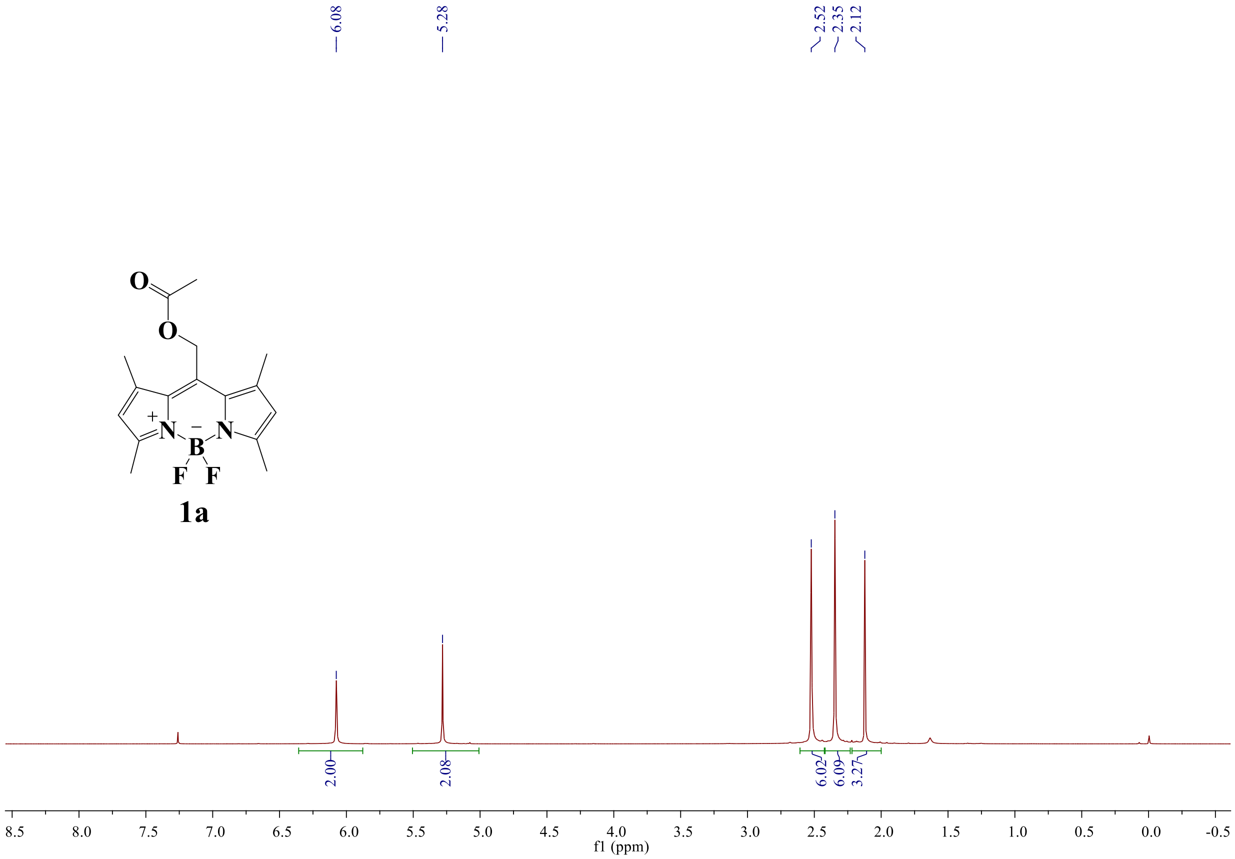

Figure S18. ^{1}H NMR spectrum of compound **1a** (400 MHz, $CDCl_3$).

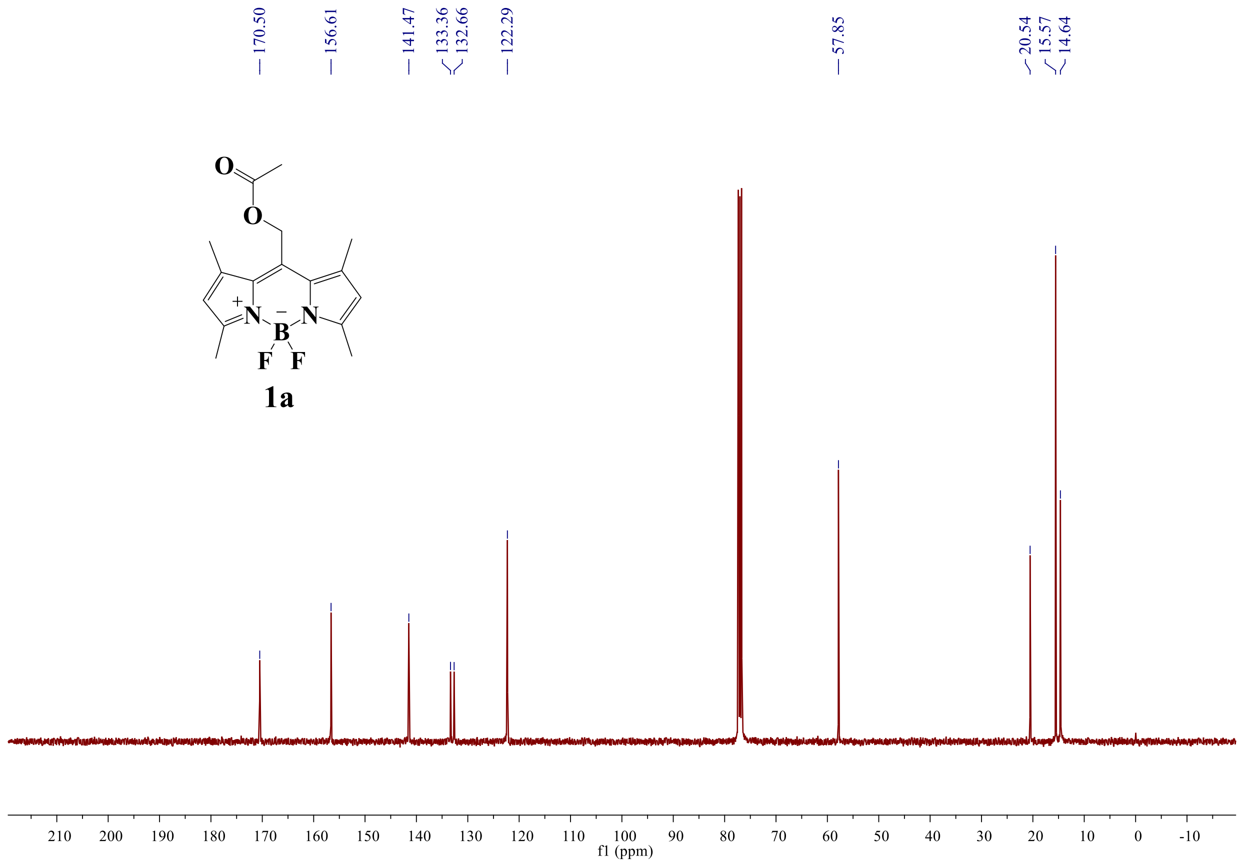

Figure S19. ^{13}C NMR spectrum of compound **1a** (100 MHz, $CDCl_3$).

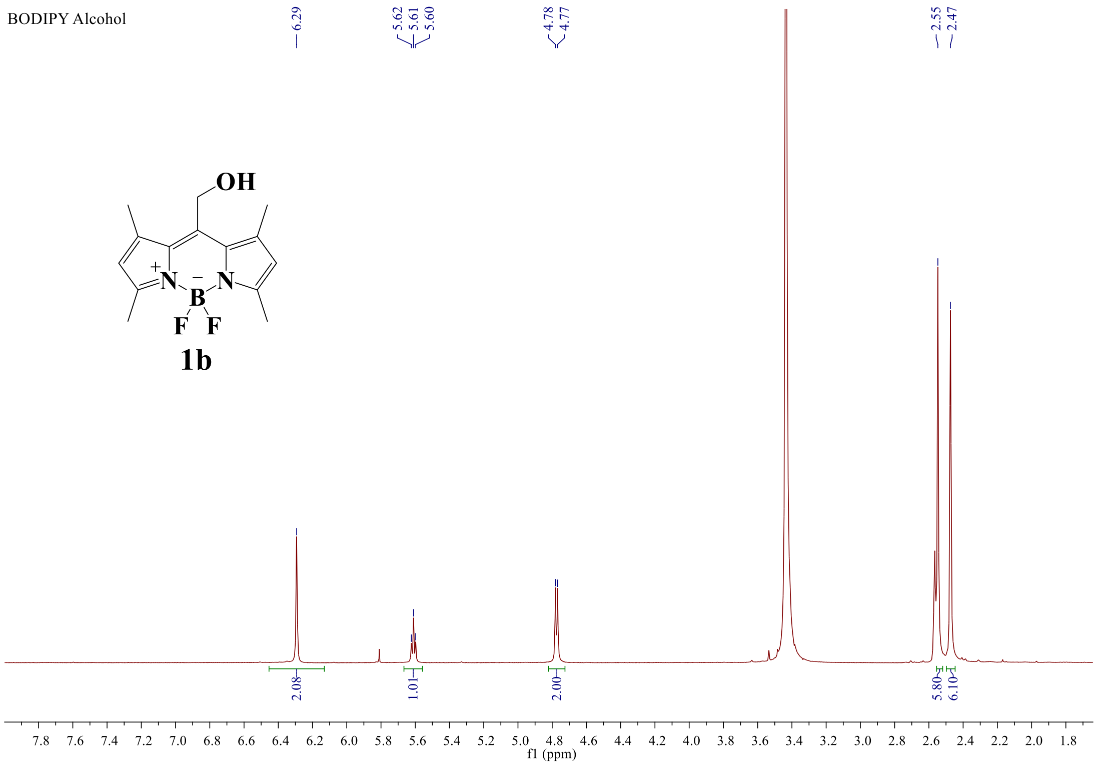

Figure S20. ^{1}H NMR spectrum of compound **1b** (400 MHz, DMSO-d_6).

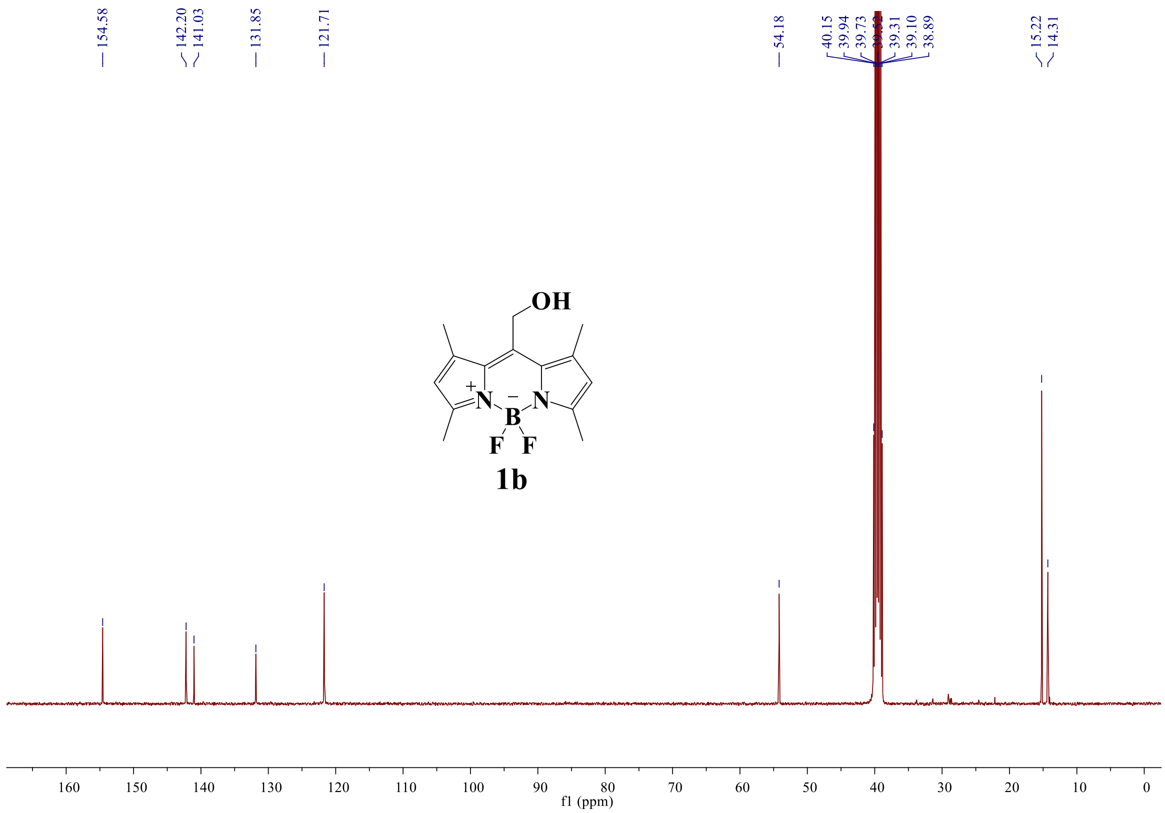

Figure S21. ^{13}C NMR spectrum of compound **1b** (100 MHz, DMSO-d_6).

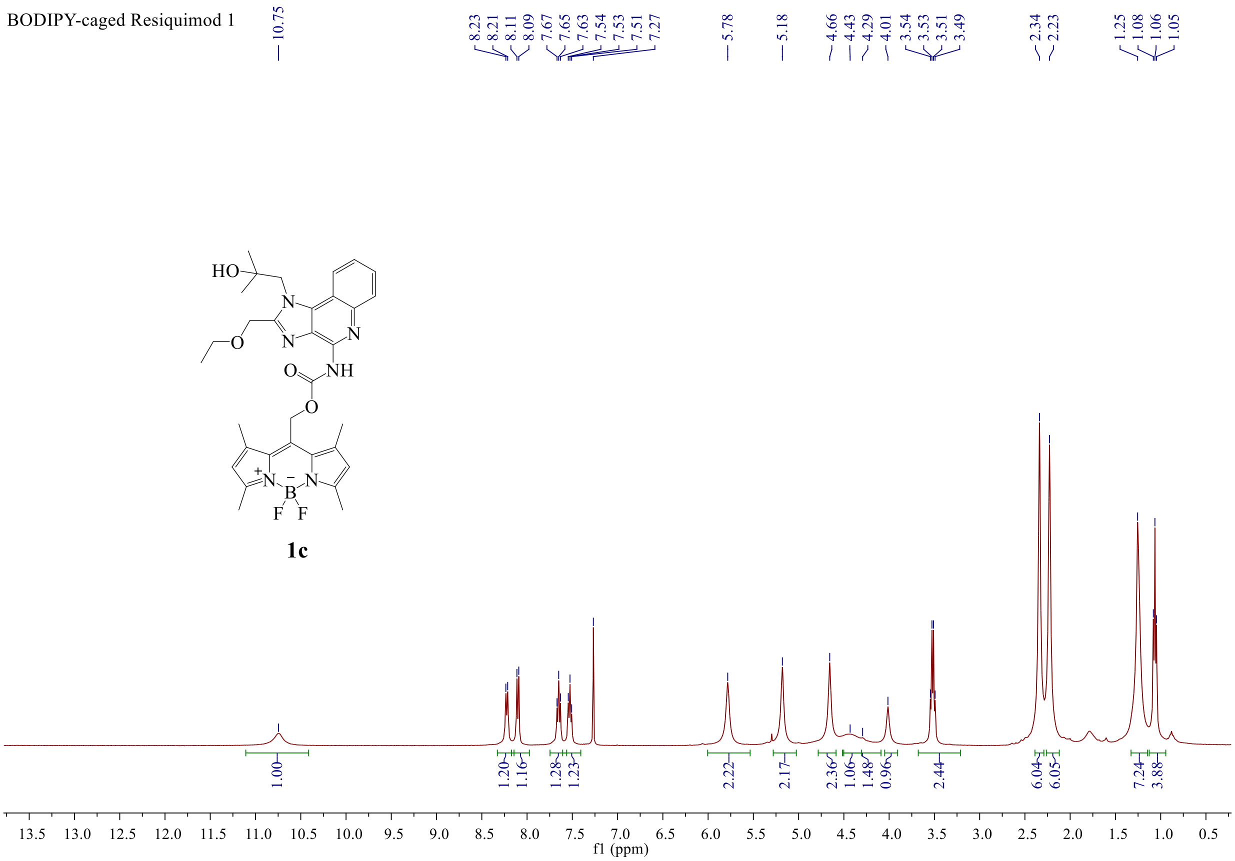

Figure S22. ^{1}H NMR spectrum of compound **1c** (400 MHz, $CDCl_3$).

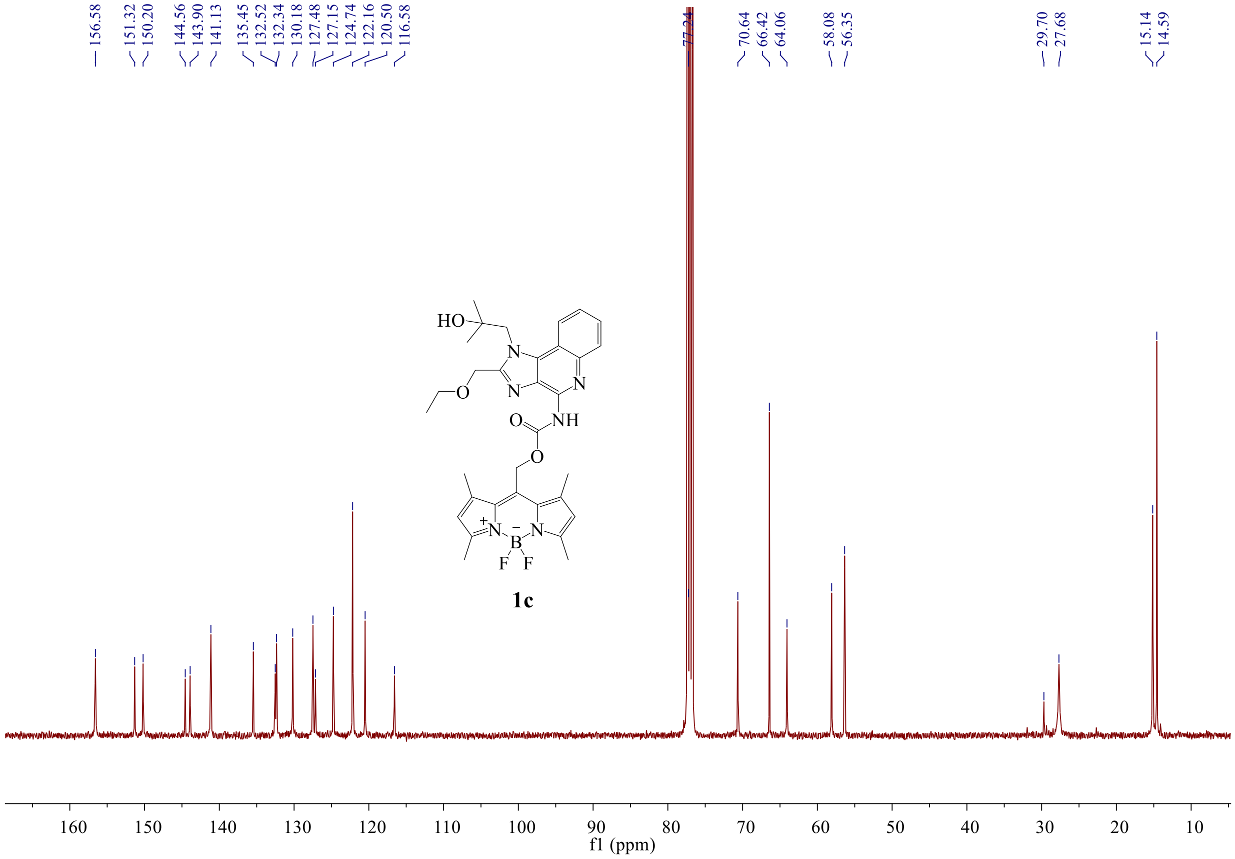

Figure S23. ^{13}C NMR spectrum of compound **1c** (100 MHz, $CDCl_3$).

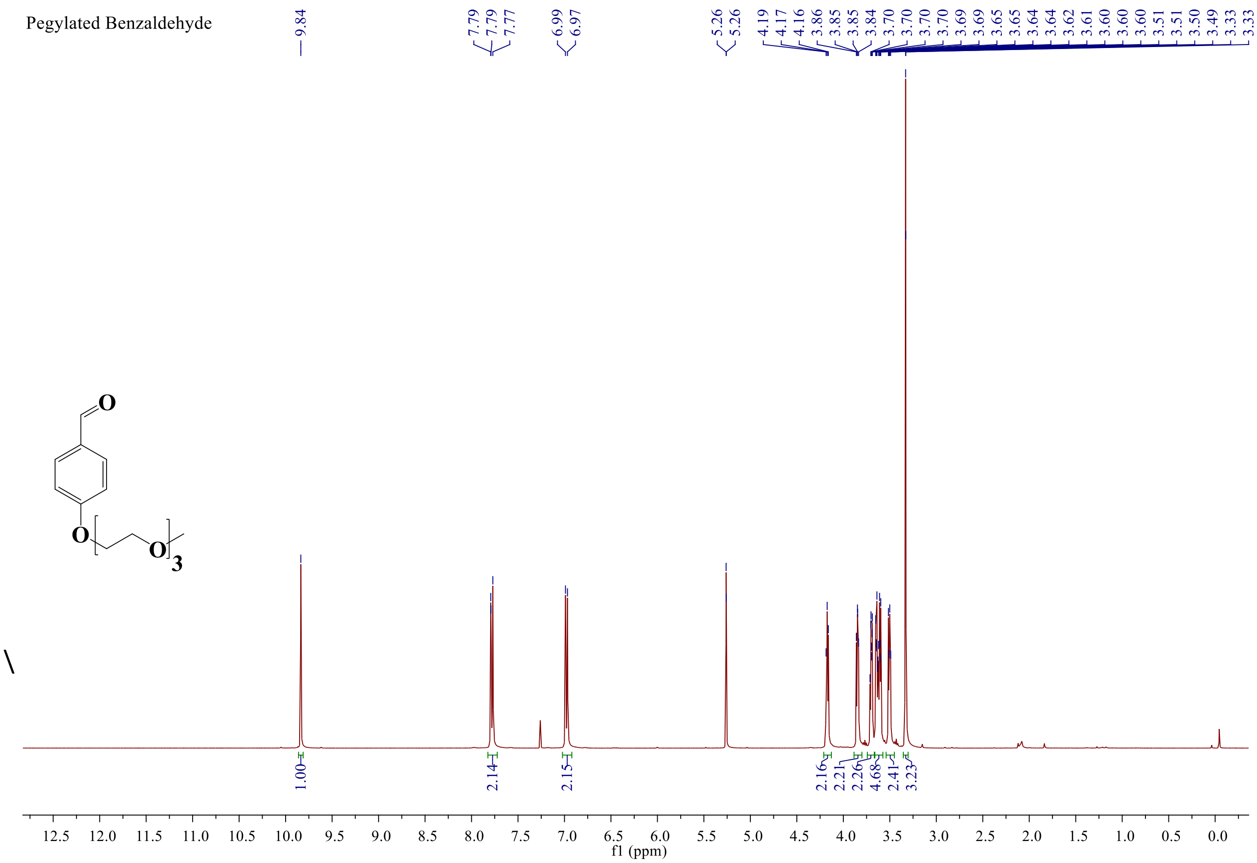

Figure S24. ^{1}H NMR spectrum of compound **2e** (400 MHz, $CDCl_3$).

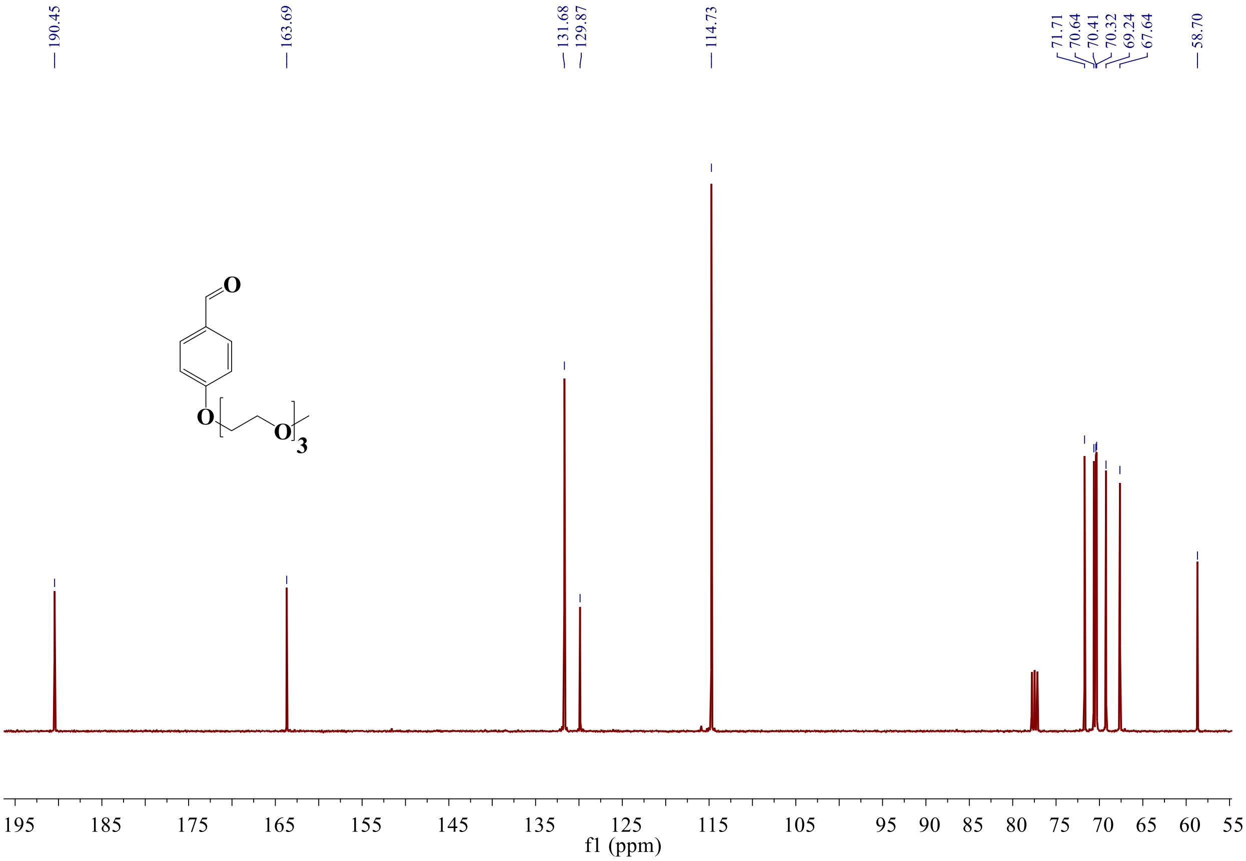

Figure S25. ^{1}H NMR spectrum of compound **2e** (400 MHz, $CDCl_3$).

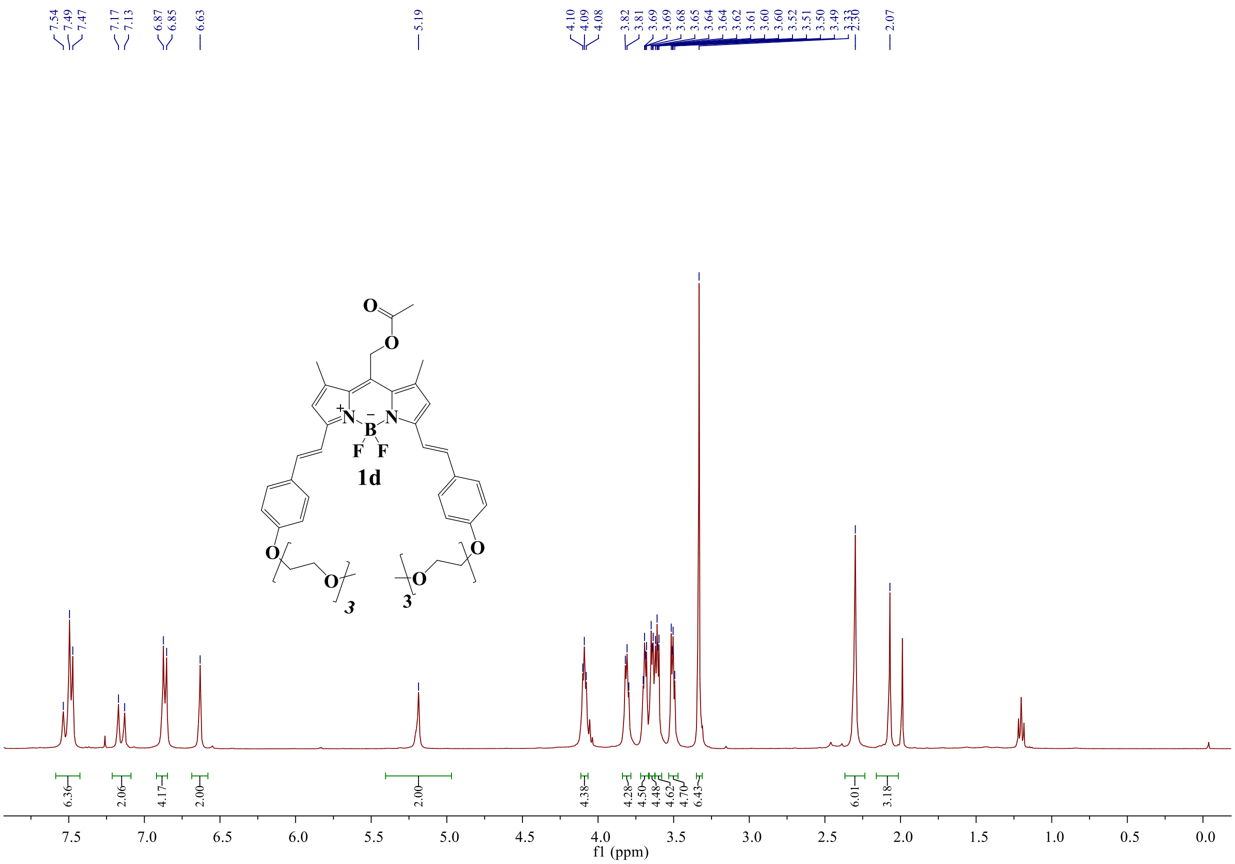

Figure S26. ^{1}H NMR spectrum of compound **1d** (400 MHz, $CDCl_3$).

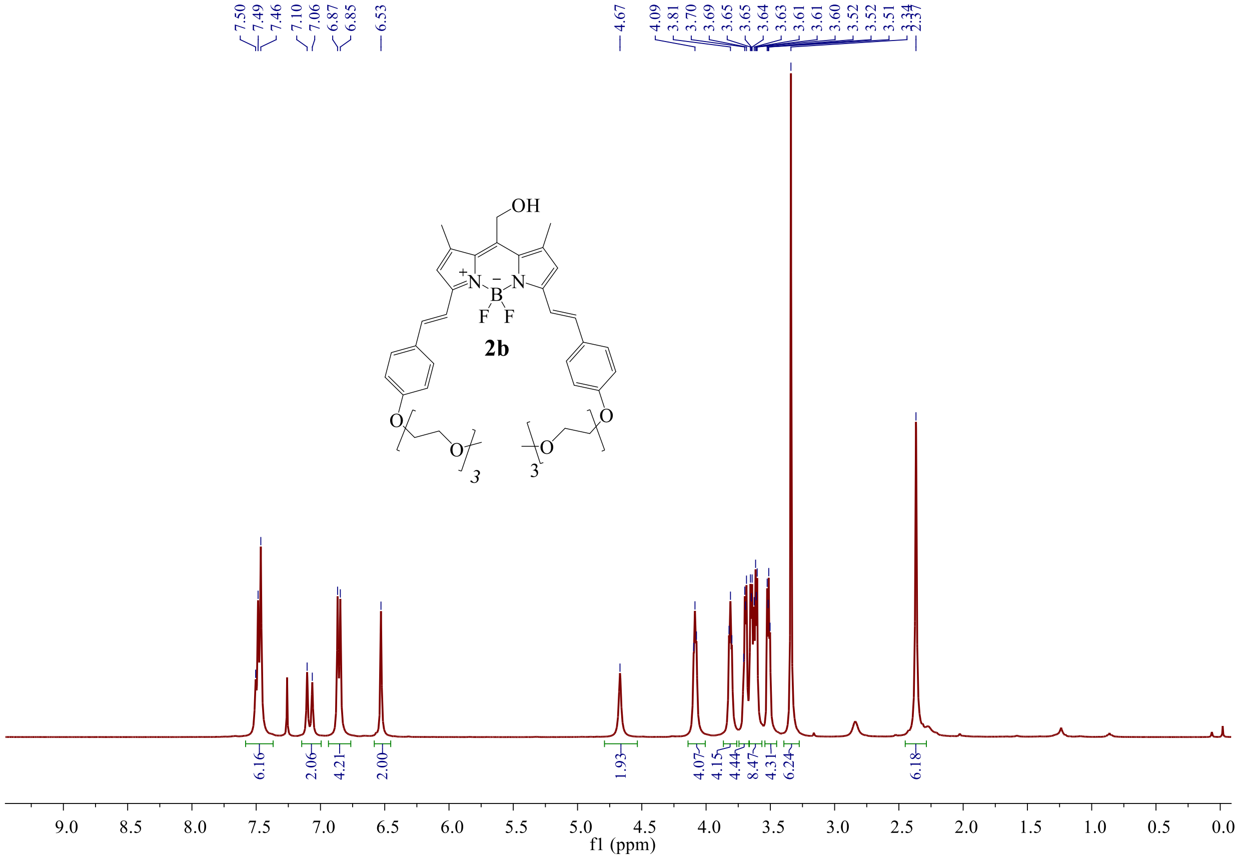

Figure S27. ^{1}H NMR spectrum of compound **2b** (400 MHz, CDCl3).

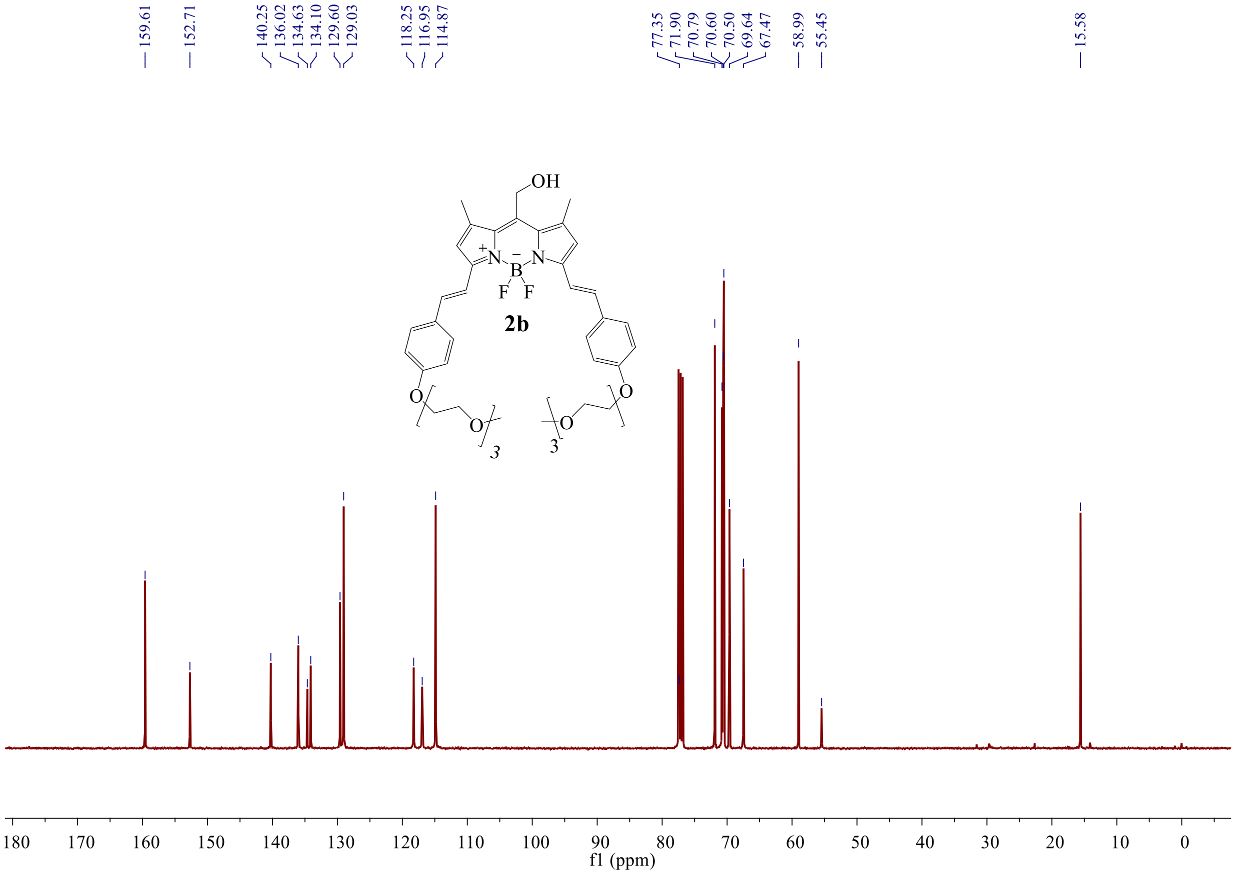

Figure S28. ^{13}C NMR spectrum of compound **2b** (100 MHz, $CDCl_3$).

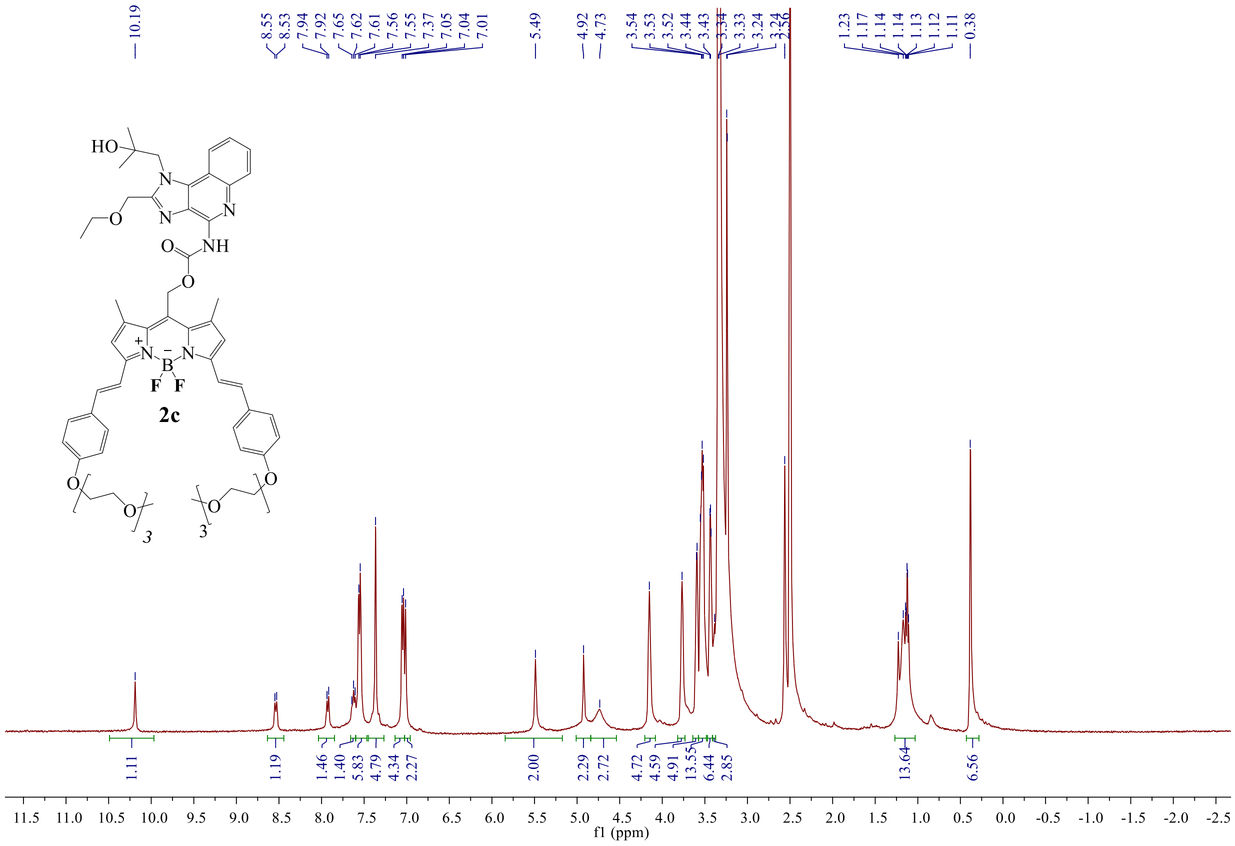

Figure S29. ^{1}H NMR spectrum of compound **2c** (400 MHz, $CDCl_3$).

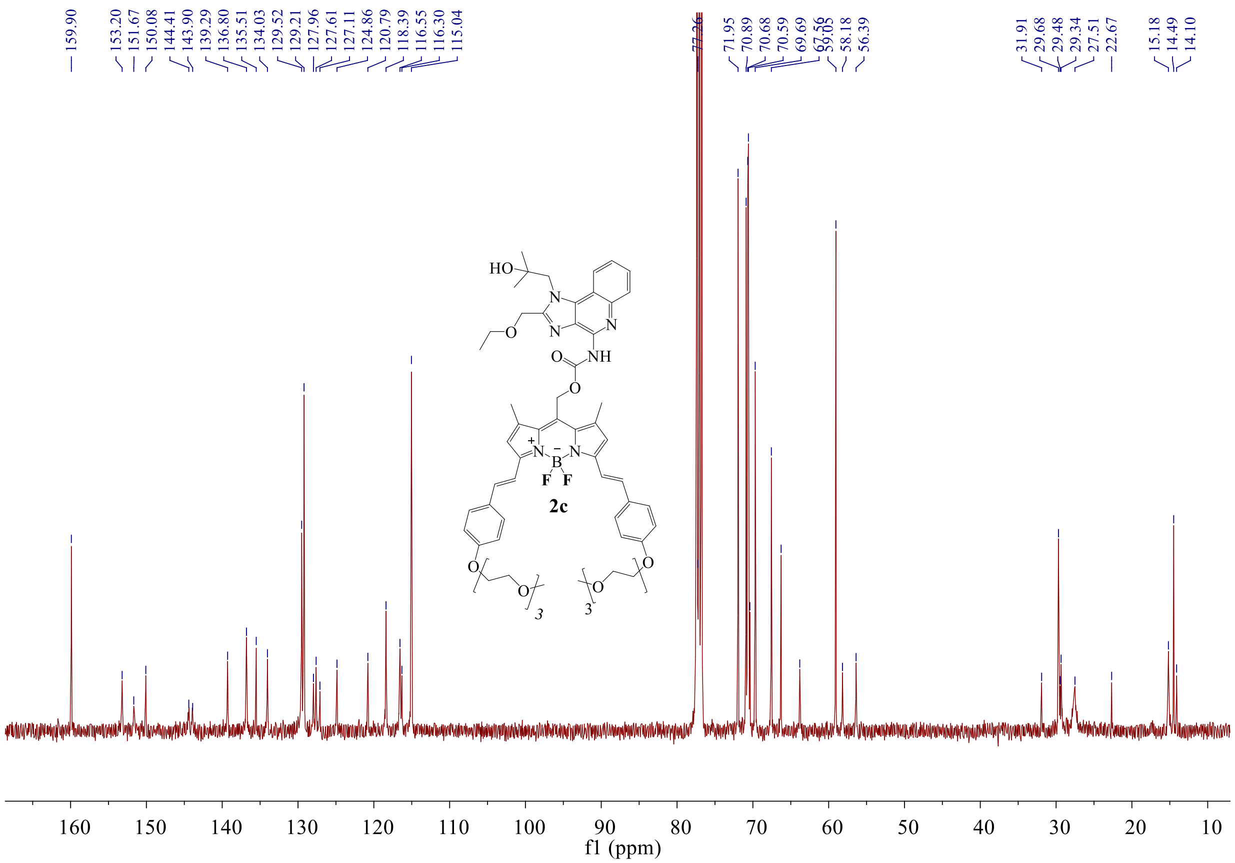

Figure S30. ^{13}C NMR spectrum of compound **2c** (100 MHz, $CDCl_3$).

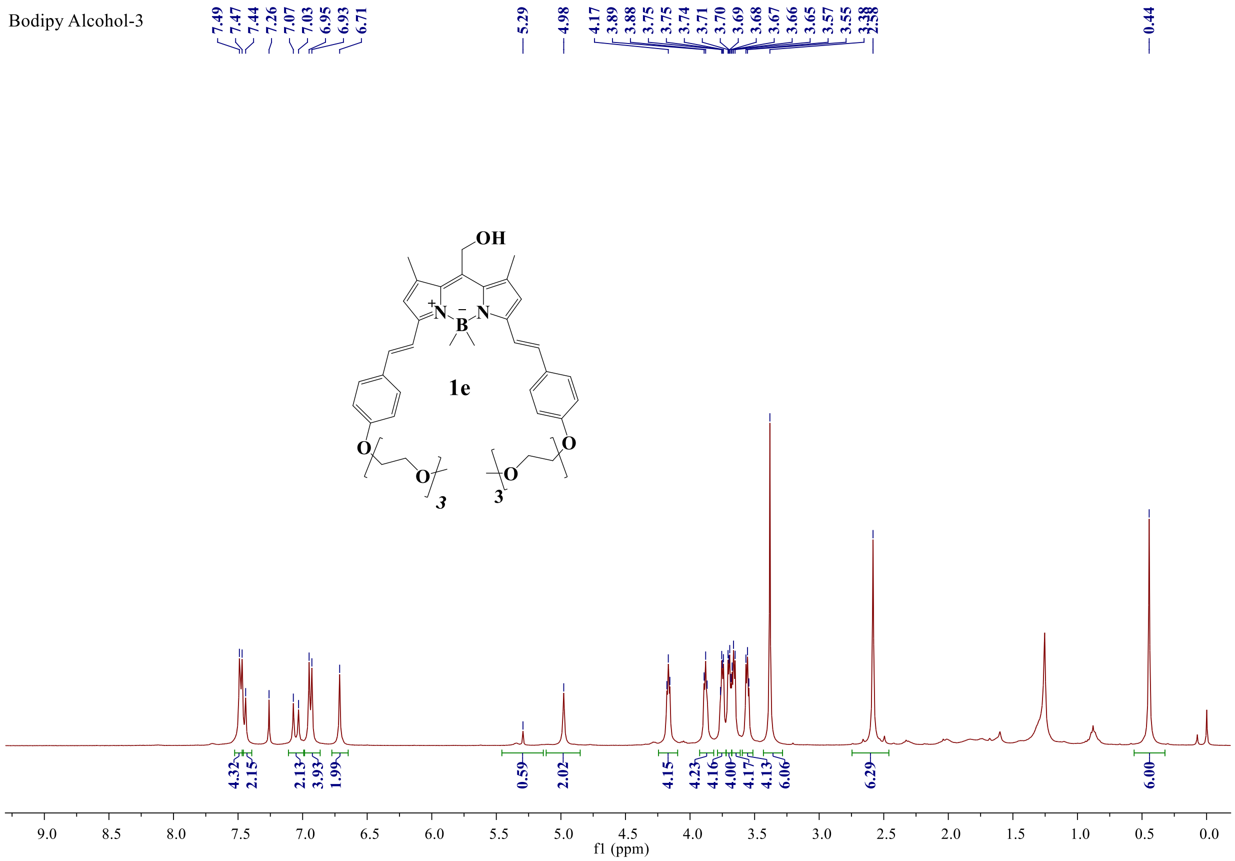

Figure S31. ^{1}H NMR spectrum of compound **1e** (400 MHz, $CDCl_3$).

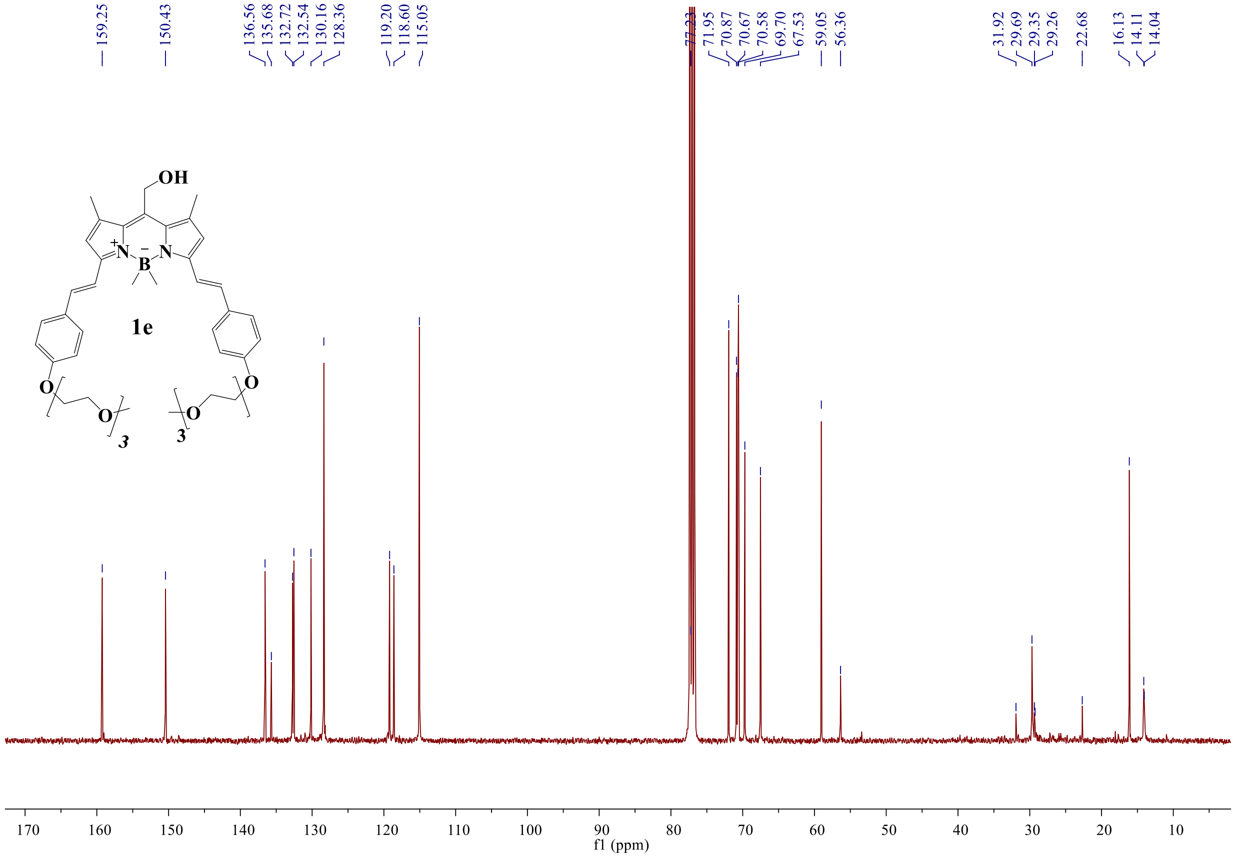

Figure S32. ^{13}C NMR spectrum of compound **1e** (100 MHz, $CDCl_3$).

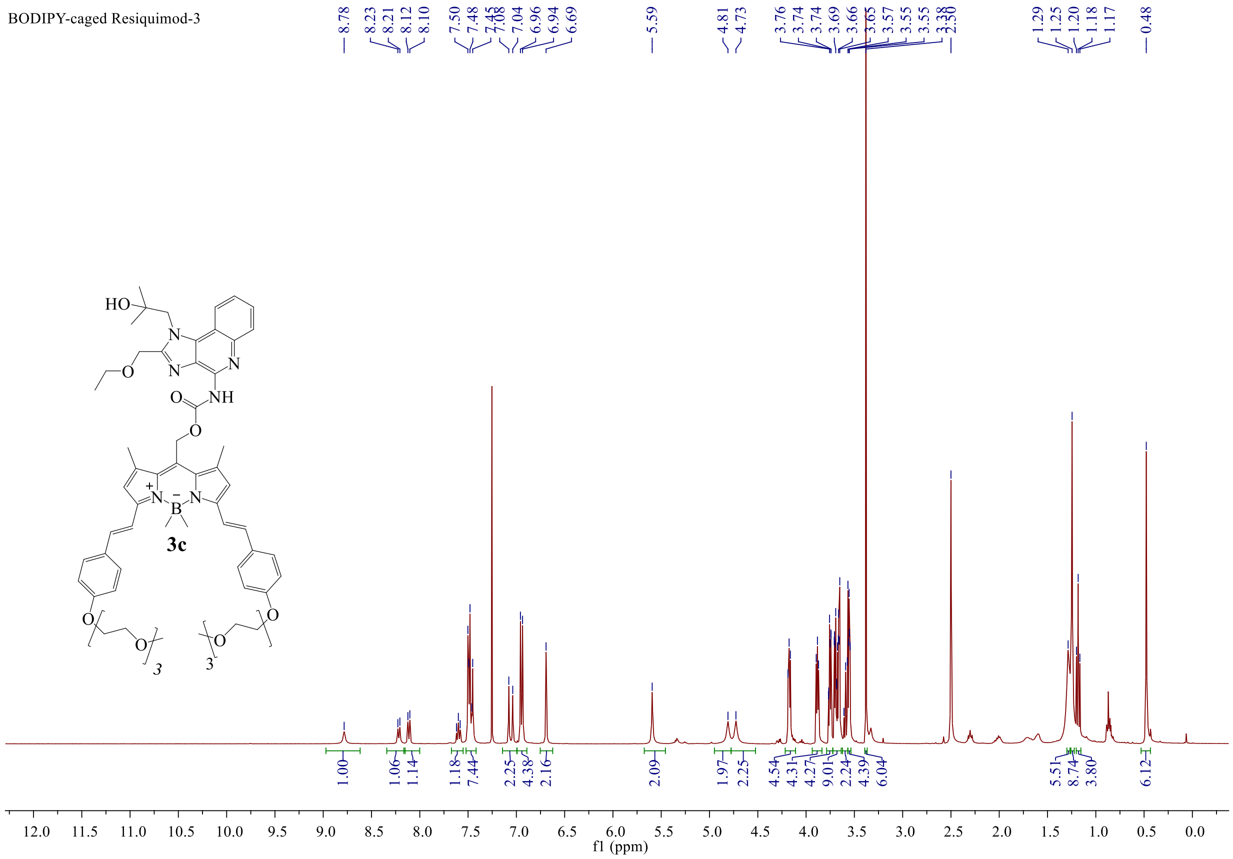

Figure S33. ^{1}H NMR spectrum of compound **3c** (400 MHz, $CDCl_3$).

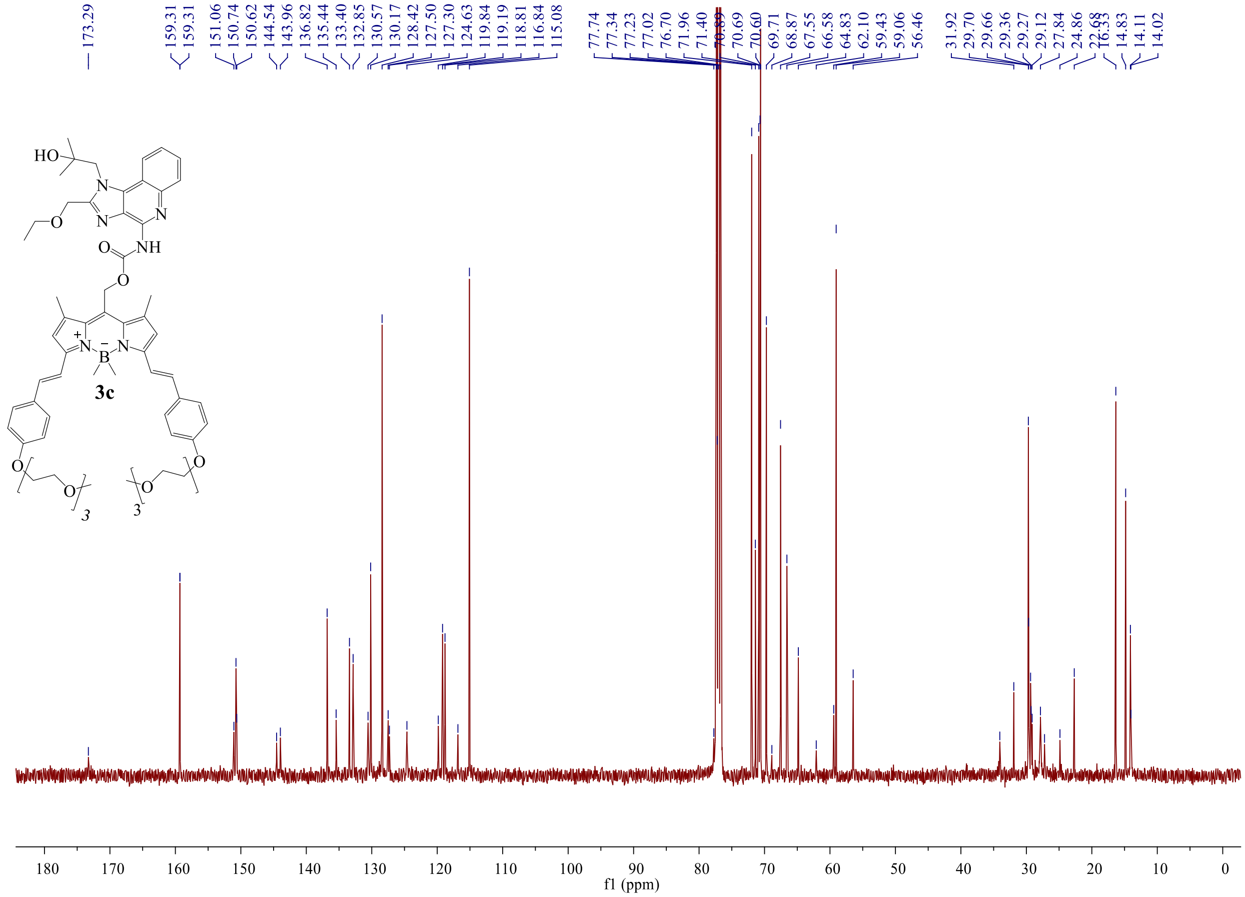

Figure S34. ^{13}C NMR spectrum of compound **3c** (100 MHz, $CDCl_3$).
